# A concise synthesis of alkyl, aryl hydropersulfides

**DOI:** 10.1039/d6ra03773a

**Published:** 2026-07-06

**Authors:** Shishir Bhowmik, Jun Yong Kang

**Affiliations:** a Department of Chemistry and Biochemistry, University of Nevada Las Vegas 4505 S. Maryland Parkway Las Vegas Nevada 89154-4003 USA junyong.kang@unlv.edu

## Abstract

A concise synthesis of alkyl- and aryl-hydropersulfides has been developed. This process involves *N*-thioacetyl succinimide-mediated synthesis of acetyl persulfides and their subsequent deacetylation. This step economical transformation demonstrated a rapid access to both alkyl and aryl hydropersulfides under catalyst-free, additive-free, and sensitive reagent-free conditions.

## Abstract 2

A concise synthesis of alkyl- and aryl-hydropersulfides has been developed.

## Graphical abstract

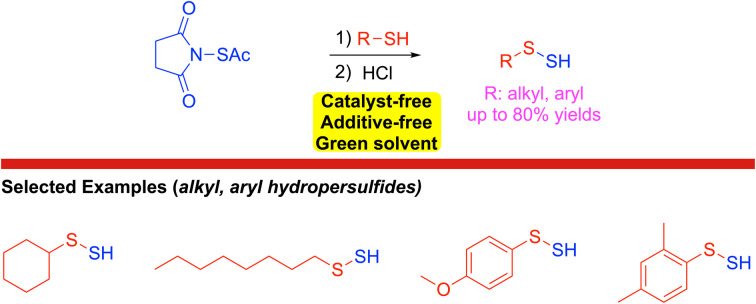

## Introduction

**Scheme 1:**
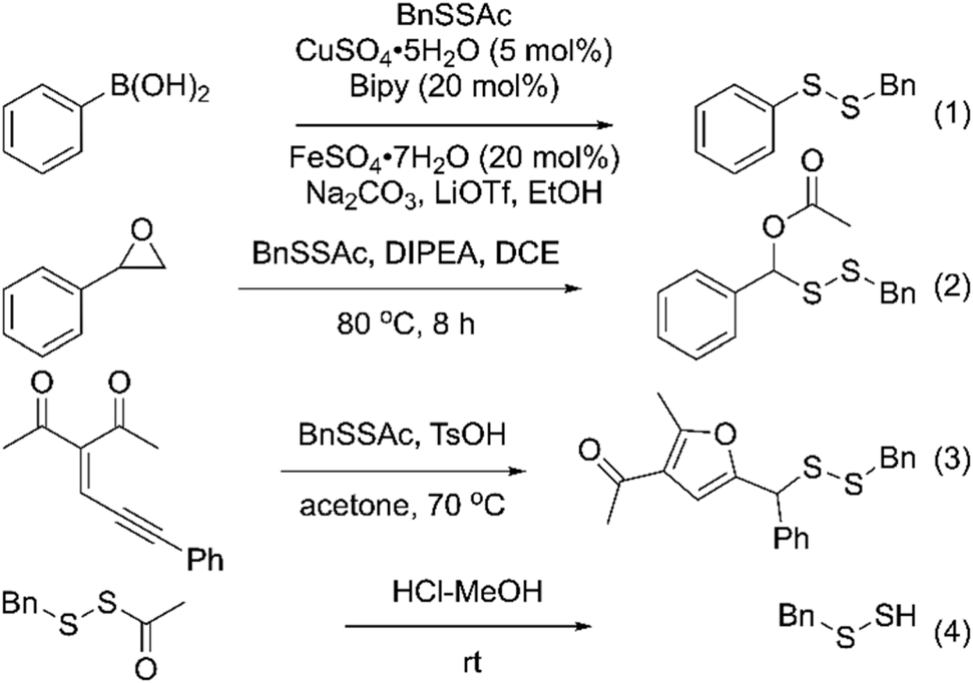
Examples of acetyl persulfide application.

Acyl persulfides are emerging reagents in organosulfur chemistry.^1–3^ For example, benzyl acetyl persulfide (BnSSAc) has found a wide range of applications. It has been utilized in metal-catalyzed cross-coupling reactions, base-mediated sulfurization reactions, and acid-promoted cyclization (Scheme 1).^4–6^ BnSSAc undergoes a Cu-catalyzed coupling reaction with boronic acid to generate unsymmetrical disulfides (Scheme 1 and eqn (1)).^4,7^ It also generates anionic BnSS nucleophile under basic conditions to form β-acetoxy disulfide (Scheme 1 and eqn (2)).^5^ Under acidic conditions, BnSSAc participates in a tandem cyclization process to form furyl methyl disulfides (Scheme 1 and eqn (3)).^6^ These BnSSAc-mediated reactions install disulfide functionality on their final products using acetyl-masked disulfide nucleophiles. On the other hand, the synthesis of an acid analog (hydropersulfide) from BnSSAc and its application in methodology remains underexplored (Scheme 1 and eqn (4)).^1^ Although deacetylation of BnSSAc under alcoholic HCl to generate BnSSH has been described, this protocol is limited mainly to alkyl acyl persulfides.^1,8^ Thus, the development of a reliable method to generate hydropersulfides is an unmet need to explore their potential application in synthetic methodology and study in physiological systems.^9–30^

Hydropersulfide exhibits unique properties such as the alpha effect and receives significant interest in physiological research.^24,26^ The alpha effect of hydropersulfides induces more nucleophilicity than the corresponding thiols.^25^ This reactivity has been used to harness green, efficient transformations.^18^ The Pluth group and Pratt group pioneered the synthetic utility of hydropersulfides and derivatives.^18,28,29^ Hydropersulfides are also found in cells and biosynthesized by sulfur-transferring enzymes.^31,32^ For example, cystine hydropersulfides have received significant attention due to their antioxidant effect, cytoprotective role, and redox signaling properties.^23,26^ Despite these important roles of hydropersulfides, access to hydropersulfides remains challenging, and some of them are partially characterized.^22,33^ Hydropersulfide is generally accessed through the deacetylation of acetyl persulfides.

**Scheme 2:**
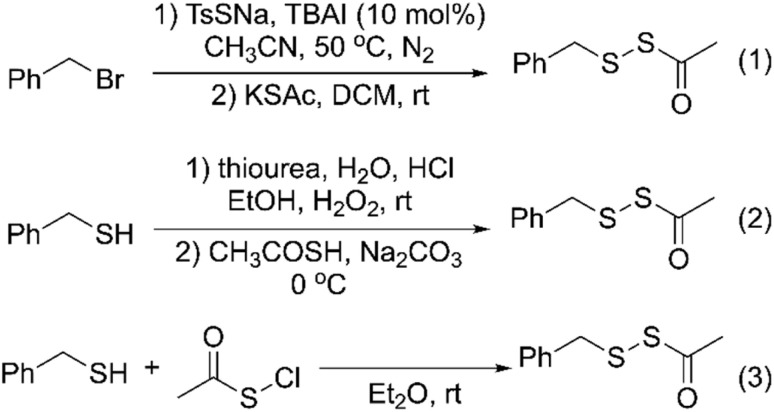
Representatives of BnSSAc synthesis.

Acetyl persulfides, a precursor of hydropersulfide, are generally accessed in a one or two-step synthesis (Scheme 2). Alkyl acetyl persulfides are synthesized through a two-step process from alkyl bromides through tosylation and acetylation (Scheme 2 and eqn (1)).^1,8^ This method, however, is limited to only alkyl halides: no aryl halides are allowed. They are also synthesized from thiols using thiourea in two steps (Scheme 2 and eqn (2)).^34^ One-step protocol to convert thiols to acetyl persulfides, however, requires a corrosive, moisture-sensitive reagent, acetylsulfenyl chloride (Scheme 2 and eqn (3)).^29,33,35–37^

**Scheme 3:**
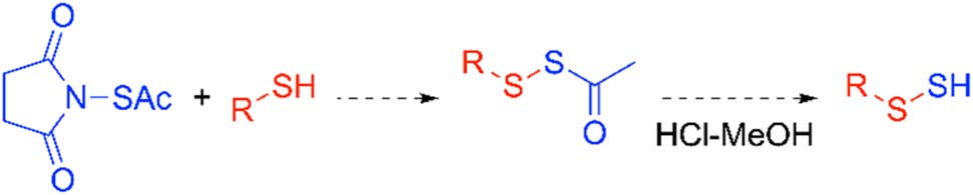
Proposed synthesis of hydropersulfide *via* acetyl persulfide.

To address the current limitations of multistep synthesis, substrate restriction, corrosive reagents, and lack of characterization, especially aryl hydropersulfides, we envisioned a direct access to both alkyl and aryl acyl persulfides, using electrophilic sulfur donors. The acyl persulfides are then converted to the corresponding hydropersulfides *via* deacylation reaction (Scheme 3). To the best of our knowledge, there is no streamlined synthesis of both alkyl and aryl hydropersulfides with full characterization.

## Result and discussion

To test our hypothesis, electrophilic sulfur donors, such as *N*-thioacetyl succinimides, were evaluated.^38–40^*N*-Thioacetyl succinimide 1a (1 equiv.) was treated with benzyl mercaptan 2a (1 equiv.) to optimize the reaction conditions (Table 1). First, optimization of reaction conditions with various solvents provided methanol as the optimal solvent. Solvent screening revealed that protic solvents (MeOH, *i*PrOH) are favored for this transformation. Presumably, in protic solvents, the N atom of *N*-thioacetyl succinimide is activated *via* a hydrogen bond, thereby polarizing the N–S bond. This polarization facilitates the nucleophilic attack of thiol to the S atom, inducing the N–S bond cleavage to form the desired acetyl persulfide product. Next, the ratio of *N*-thioacetyl succinimide 1a and benzyl mercaptan 2a was tested. A slight excess of thiol nucleophile 2a (1.5 equiv.) is needed for high product yield (Table 1, entry 9). However, a further increment of the nucleophile to a 2.0 equivalent did not improve the product yield (Table 1, entry 10). It is noteworthy that a large excess of thiol nucleophile exacerbates the formation of the disulfide byproduct,^38^ because excess thiols can further react with the acetyl persulfide product. Lastly, to evaluate a temperature factor for the reaction conditions, reactions were run at 0 °C and 60 °C (Table 1, entries 11 and 12), respectively. The temperature variation on the reaction conditions did not have a positive effect on the product yields, providing 45% and 69% yields, respectively. The high temperature condition presumably diminishes the stability of *N*-thioacetyl succinimide and thus aggravates the decomposition to succinimide. Also, higher temperature resulted in a greater extent of disulfide formation. This optimization process provided the desired product 3a with 80% yield at room temperature under a slight excess of thiol nucleophile (1.5 equiv.).

**Table 1:** Optimization of alkyl acetyl persulfide synthesis^a^

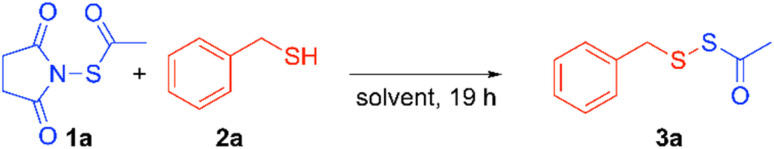				
Entry	1a : 2a	Solvent	Temperature	3a Yield (%)^b^
1	1.0 : 1.0	ACN	rt	11
2	1.0 : 1.0	Acetone	rt	20
3	1.0 : 1.0	DCM	rt	0
4	1.0 : 1.0	MeOH	rt	61
5	1.0 : 1.0	EtOH	rt	13
6	1.0 : 1.0	*i*PrOH	rt	54
7	1.0 : 1.0	*t*BuOH	rt	10
8	1.0 : 1.0	HFIP	rt	9
**9**	**1.0** : **1.5**	**MeOH**	**rt**	**80**
10	1.0 : 2.0	MeOH	rt	72
11	1.0 : 1.5	MeOH	0 °C	45
12	1.0 : 1.5	MeOH	60 °C	69

a Reaction conditions: 1a (2 mmol) and 2a (3 mmol) in solvent (10 mL) for 19 h.

b Isolated yield.

**Scheme 4:**
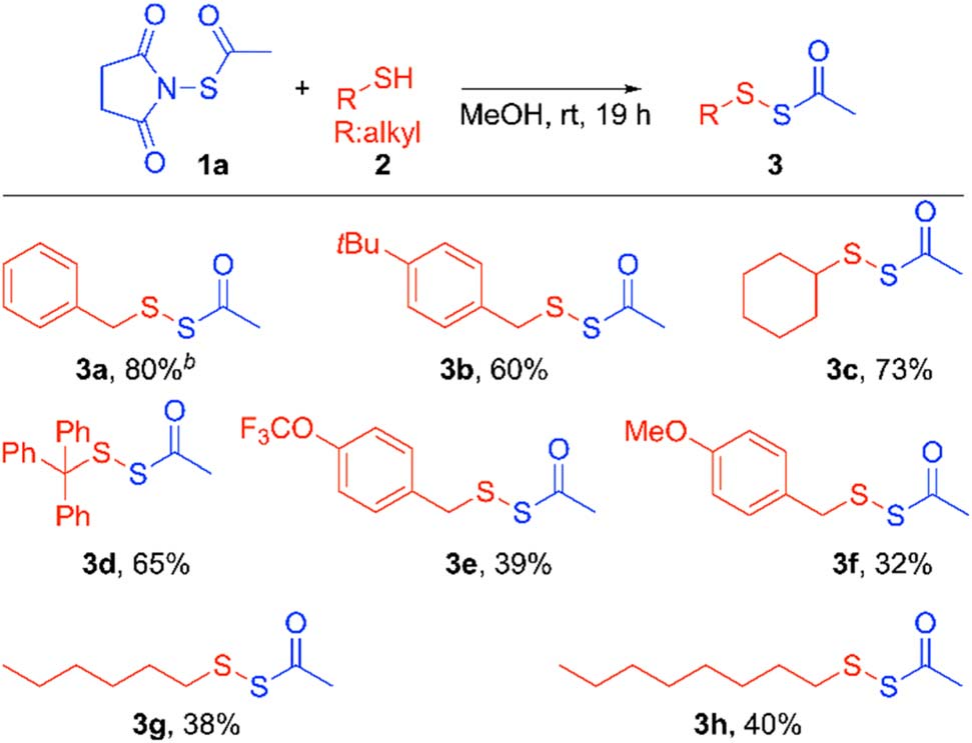
Substrate scope of alkylthiols^*a*^. ^*a*^Reaction conditions: 1a (2 mmol) and 2 (3 mmol) in MeOH (10 mL) for 19 h. ^*b*^Isolated yield.

With optimized reaction conditions in hand, the scope of alkyl thiols was evaluated to study the steric and electronic effects on the reaction outcome (Scheme 4). First, the steric effect was evaluated by testing *para*-tertbutyl benzyl mercaptan 2b, cyclohexyl thiol 2c, and trityl mercaptan 2d. Both primary and secondary thiols were well tolerated to give the corresponding products 3b and 3c with 60% and 73% yields, respectively. Moreover, a tertiary thiol 2d also provided the desired product 3d with 65% yield. Next, electron-withdrawing and donating groups (2e and 2f) on a para position of benzyl mercaptan provided the corresponding products 3e and 3f with 39% and 32% yields, respectively. Long alkyl chain thiols, such as hexyl and octyl thiols, 2g and 2h, were also tolerated to give the desired products with 38% and 40%, respectively. It is noteworthy that a large-scale reaction (10 mmol) provided the desired product with 62% yield (1.23 g). Overall, various substrates, including tertiary and alkyl chain thiols, were demonstrated to generate the target alkyl acetyl persulfides in moderate to high yields.

**Scheme 5:**
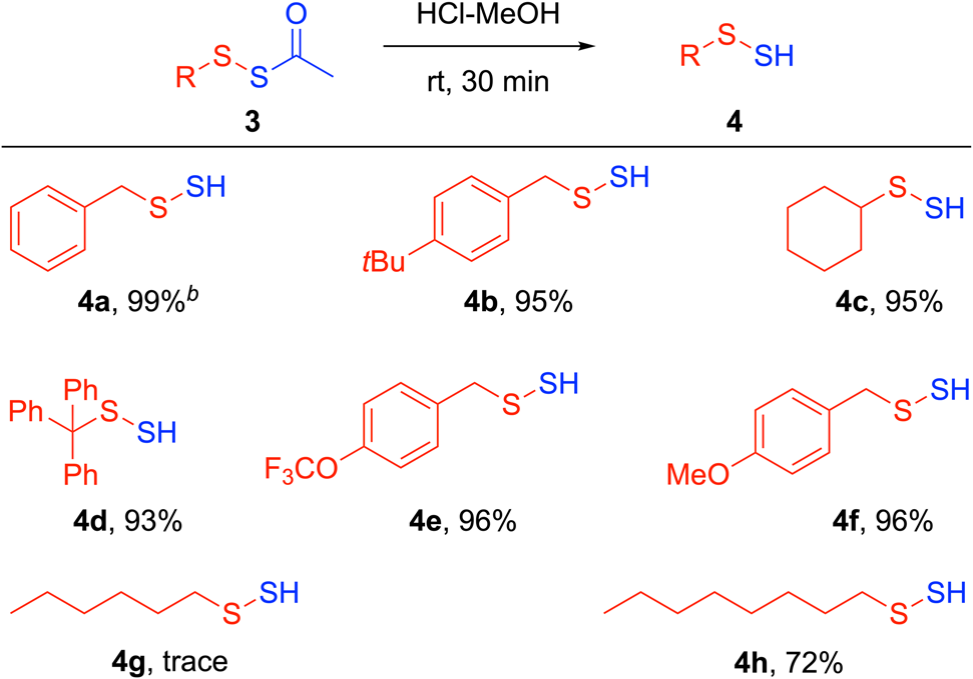
Substrate scope of alkyl acetyl persulfides^*a*^. ^*a*^Reaction conditions: 3 (0.5 mmol) and HCl (2.5 mmol) in MeOH (1 mL) for 30 min. ^*b*^Isolated yield.

These alkyl acetyl persulfides were subjected to a deacetylation reaction to generate hydropersulfides (Scheme 5). They were smoothly converted to the corresponding hydropersulfides under HCl–MeOH conditions.^1^ Primary, secondary, and tertiary acetyl persulfides provided the desired products in excellent yields (93–99%). The alkyl chain acetyl persulfides 3g and 3h were sluggish to afford the target hydropersulfides 4g and 4h with a trace amount and a 72% yield, respectively. In addition, the practicality of this transformation was demonstrated with a 2.5 mmol scale reaction of 3a, providing 99% yield of 4a. It is noteworthy to mention that hydropersulfides are gradually oxidized to form polysulfides at room temperature, although the stability is extended at lower temperature.

Next, we were intrigued by the synthesis of aryl hydropersulfides. Compared to alkyl hydropersulfides, aryl hydropersulfides were understudied due to the challenges of the synthesis and the lack of characterization data.^22^ A series of parameters was optimized (Table 2). The solvent study provided EtOH as the optimal solvent among other protic and aprotic solvents (Table 2, entry 5). No excess amount of thiol nucleophile benefited for the optimization of reaction conditions (Table 2, entries 8 and 9), presumably due to the formation of disulfide byproducts with excess nucleophiles. The temperature variation did not improve yields (Table 2, entries 10 and 11). Overall, the optimized conditions need an equal ratio of reactants, EtOH solvent, and room temperature.

**Table 2:** Reaction optimization of aryl acetyl persulfide synthesis^a^

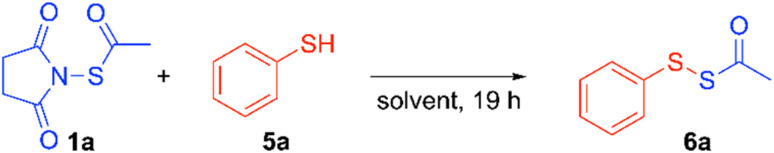				
Entry	1a : 5a	Solvent	Temperature	6a Yield (%)^b^
1	1.0 : 1.0	ACN	rt	21
2	1.0 : 1.0	Acetone	rt	22
3	1.0 : 1.0	DCM	rt	0
4	1.0 : 1.0	MeOH	rt	30
**5**	**1.0** : **1.0**	**EtOH**	**rt**	**58**
6	1.0 : 1.0	*i*PrOH	rt	0
7	1.0 : 1.0	*t*BuOH	rt	0
8	1.0 : 1.5	EtOH	rt	40
9	1.0 : 2.0	EtOH	rt	31
10	1.0 : 1.5	EtOH	0 °C	43
11	1.0 : 1.5	EtOH	60 °C	30

a Reaction conditions: 1a (2 mmol) and 5a (2 mmol) in solvent (10 mL) for 19 h.

b Isolated yield.

**Scheme 6:**
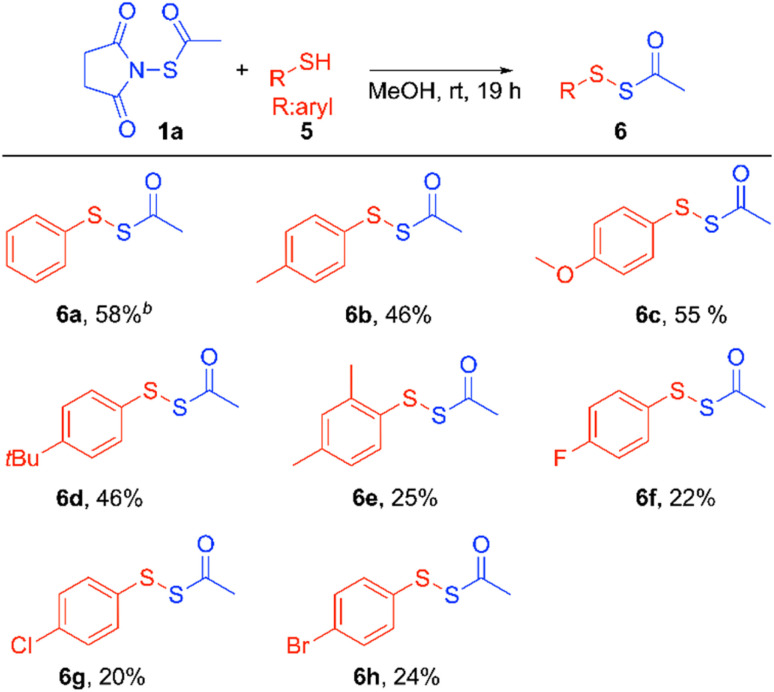
Substrate scope of arylthiols^*a*^. ^*a*^Reaction conditions: 1a (2 mmol) and 5 (2 mmol) in MeOH (10 mL) for 19 h. ^*b*^Isolated yield.

Having the optimized reaction conditions in hand, aryl thiols with electronic variation were evaluated for the synthesis of aryl acetyl persulfides (Scheme 6). The parent thiophenol 5a and electron-donating groups at the para position (5b–5d) provided the corresponding products 6a–6d with moderate yields (46–58%). Sterically hindered 2,4-dimethyl thiophenol 5e reduced the product yield (6e, 25%). Halogen-substituted thiols 5f–5h generated the desired products 6f–6h with lower yields (20–24%). In this case, a mixture of acetyl sulfide, acetyl persulfide, and polysulfide was produced, presumably due to the inductive effect induced by halogen substituents. To improve the product selectivity, a reaction at 0 °C was tested, but no positive effect on the product yield was observed. Generally, the synthesis of aryl acyl persulfides is more challenging than that of the alkyl acyl persulfides, especially with electron-deficient thiols.

**Scheme 7:**
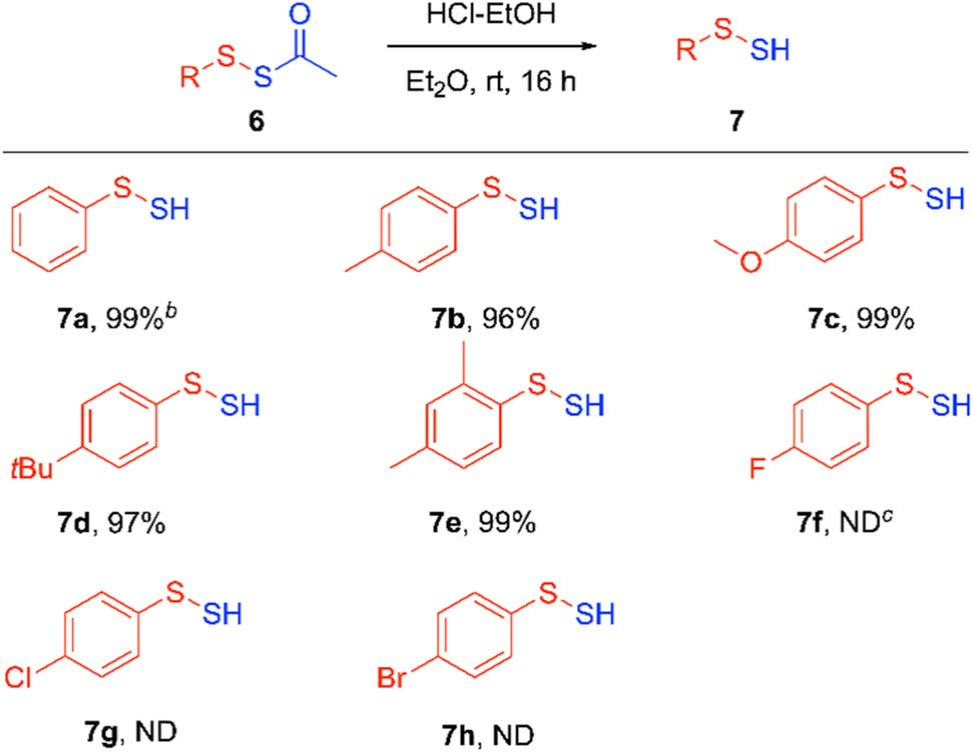
Substrate scope of aryl acetyl persulfides^*a*^. ^*a*^Reaction conditions: 6 (0.5 mmol) and HCl–EtOH (1.5 mmol) in Et_2_O (1 mL) for 16 h. ^*b*^Isolated yield. ^*c*^ND: not determined.

With the successful synthesis of different aryl acetyl persulfides, the deacetylation reaction to generate aryl hydropersulfides was examined (Scheme 7). The parent phenyl acetyl persulfide 6a and the electron-rich aryl acetyl persulfides 6b–6e were tested under HCl–EtOH, and they all provided the corresponding aryl hydropersulfide products 7a–7e with high to excellent yields (96–99%). However, deacetylation of halogen-substituted aryl acetyl persulfides 6f–6h was challenging in forming the desired aryl hydropersulfides 7f–7h with high purity. In this case, a mixture of the target hydropersulfides, decomposed thiols, and disulfide byproducts was generated. The inductive effect by halogens on the aryl acetyl persulfides 6f–6h could contribute to the formation of these mixtures in the deacetylation reaction. NMR analysis of the reaction mixtures revealed that the hydropersulfides are the major product. However, further purification using column chromatography was unsuccessful due to the decomposition caused by silica gel. Overall, our study demonstrated a practical synthesis of aryl hydropersulfides with the electron-rich thiols and provided full characterization of aryl hydropersulfides for the first time.

## Conclusions

In summary, we have developed an efficient method to synthesize hydropersulfides using *N*-thioacetyl succinimide as an electrophilic sulfur donor. This method enables step economical access to both alkyl and aryl hydropersulfides *via* deacetylation of acetyl persulfide intermediates. It also addresses the challenges in hydropersulfide synthesis: access to aryl hydropersulfides and their characterization. Although there are still some challenges for the synthesis of electron-deficient aryl hydropersulfides, such as halogenated hydropersulfides, this method demonstrated a rapid access to both alkyl and aryl hydropersulfides, which can contribute to the study of physiological systems and synthetic methodology. Further studies to understand the fundamental reactivity of these hydropersulfide compounds and their application to methodology development are under investigation, and they will be reported elsewhere.

## Conflicts of interest

There are no conflicts.

## Supplementary Material

RA-OLF-D6RA03773A-s001

RA-OLF-D6RA03773A-s002

## Data Availability

The data supporting this article have been included as part of the supplementary information (SI). Supplementary information: experimental procedures, chracterization data, and spectroscopic data. See DOI: https://doi.org/10.1039/d6ra03773a.
