# Supplementary material for: A concise synthesis of alkyl, aryl hydropersulfides
Source: RSC Adv. 2026 Jul 6;16(36):37522–6. doi: 10.1039/d6ra03773a (PMC13334445; doi:10.1039/d6ra03773a)

Supplementary Information (SI) for RSC Advances.
This journal is © The Royal Society of Chemistry 2026

A Concise Synthesis of Alkyl, Aryl Hydropersulfides

Shishir Bhowmik, Jun Yong Kang*

junyong.kang@unlv.edu

Department of Chemistry and Biochemistry, University of Nevada Las Vegas,
4505 S. Maryland Parkway, Las Vegas, NV 89154-4003

Supporting Information

^{1}H and ^{13}C NMR Spectra……………………………………………………………S-2

^{1}H NMR (400 MHz) in $CDCl_3$

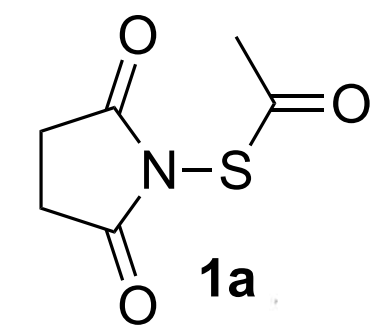

1a

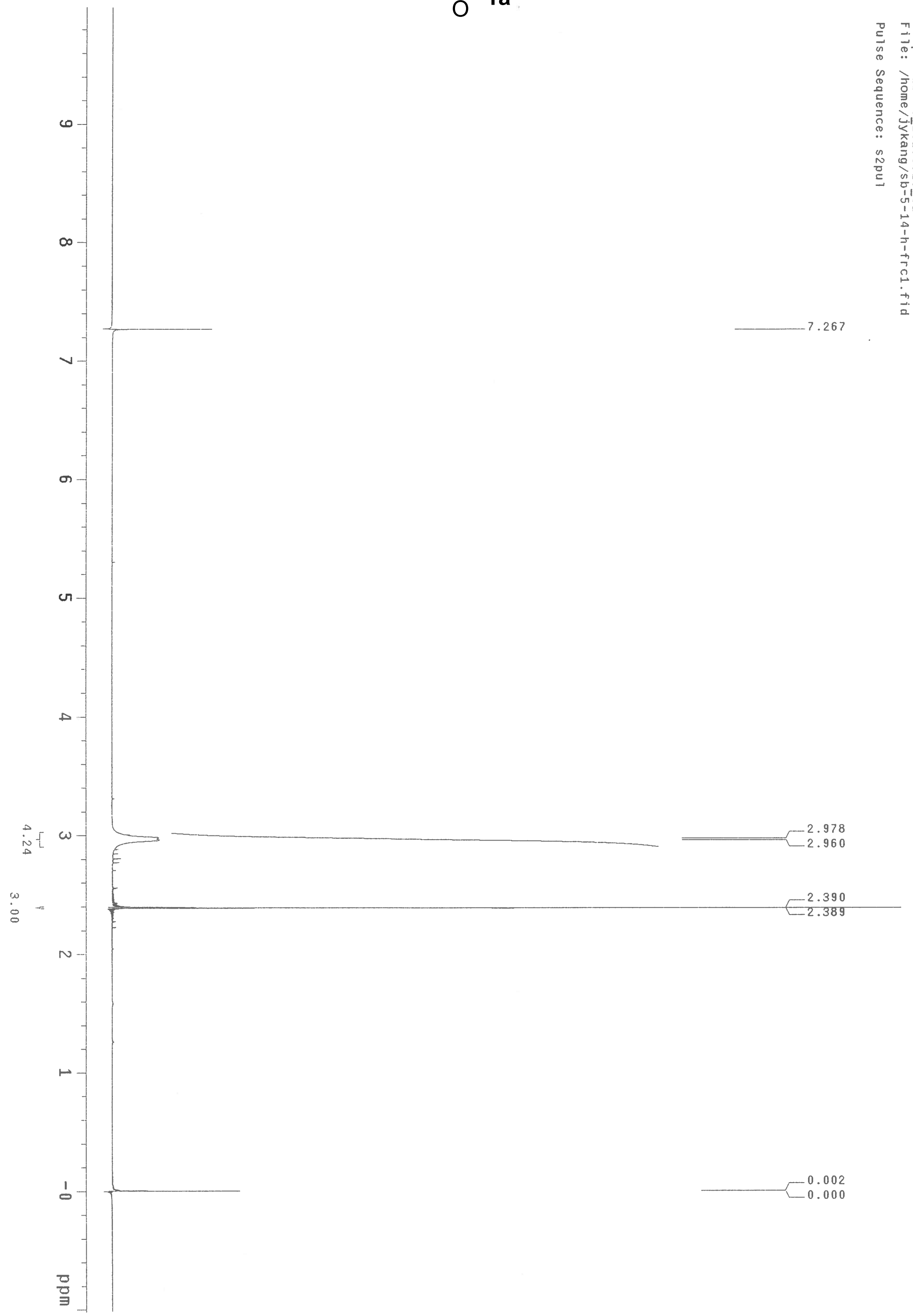

^{13}C NMR (100.5 MHz) in $CDCl_3$

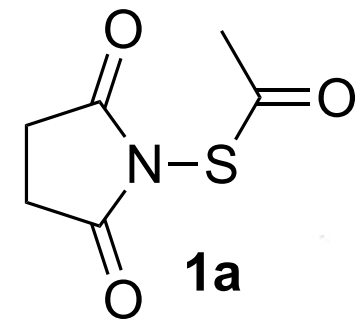

1a

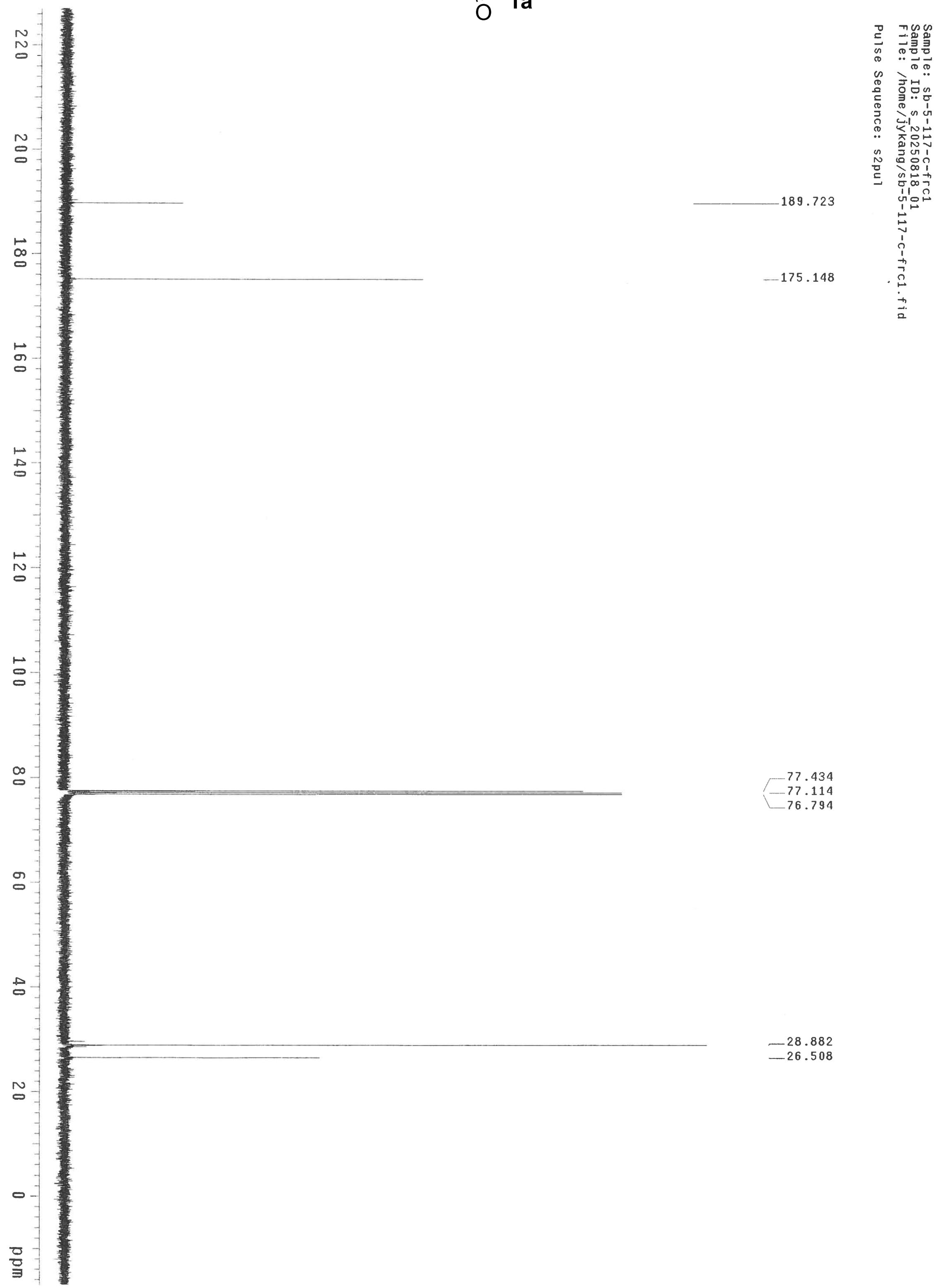

^{1}H NMR (400 MHz) in $CDCl_3$

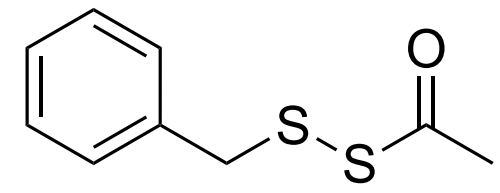

3a

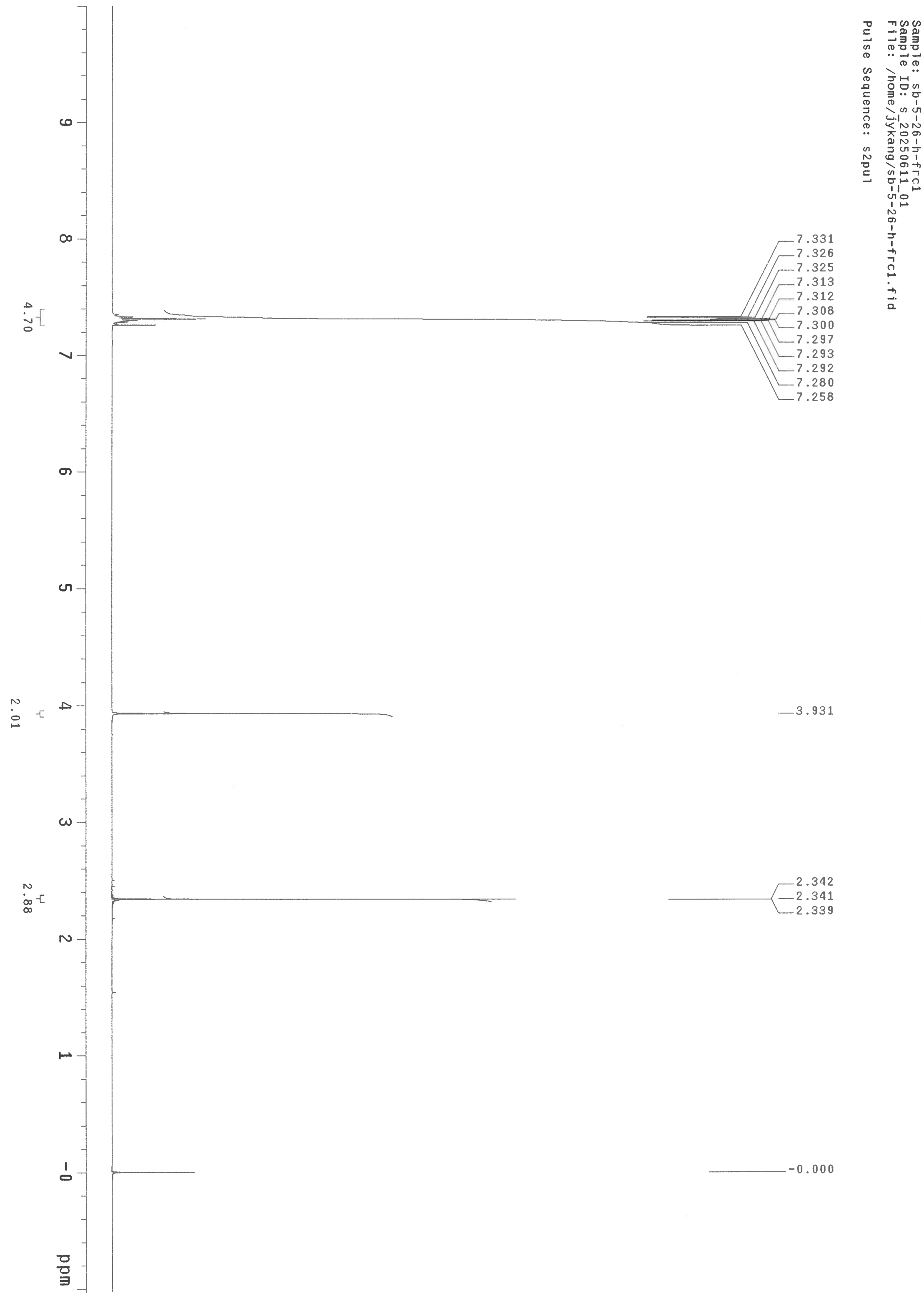

^{13}C NMR (100.5 MHz) in CDCl$_3$

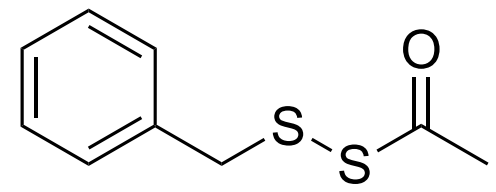

3a

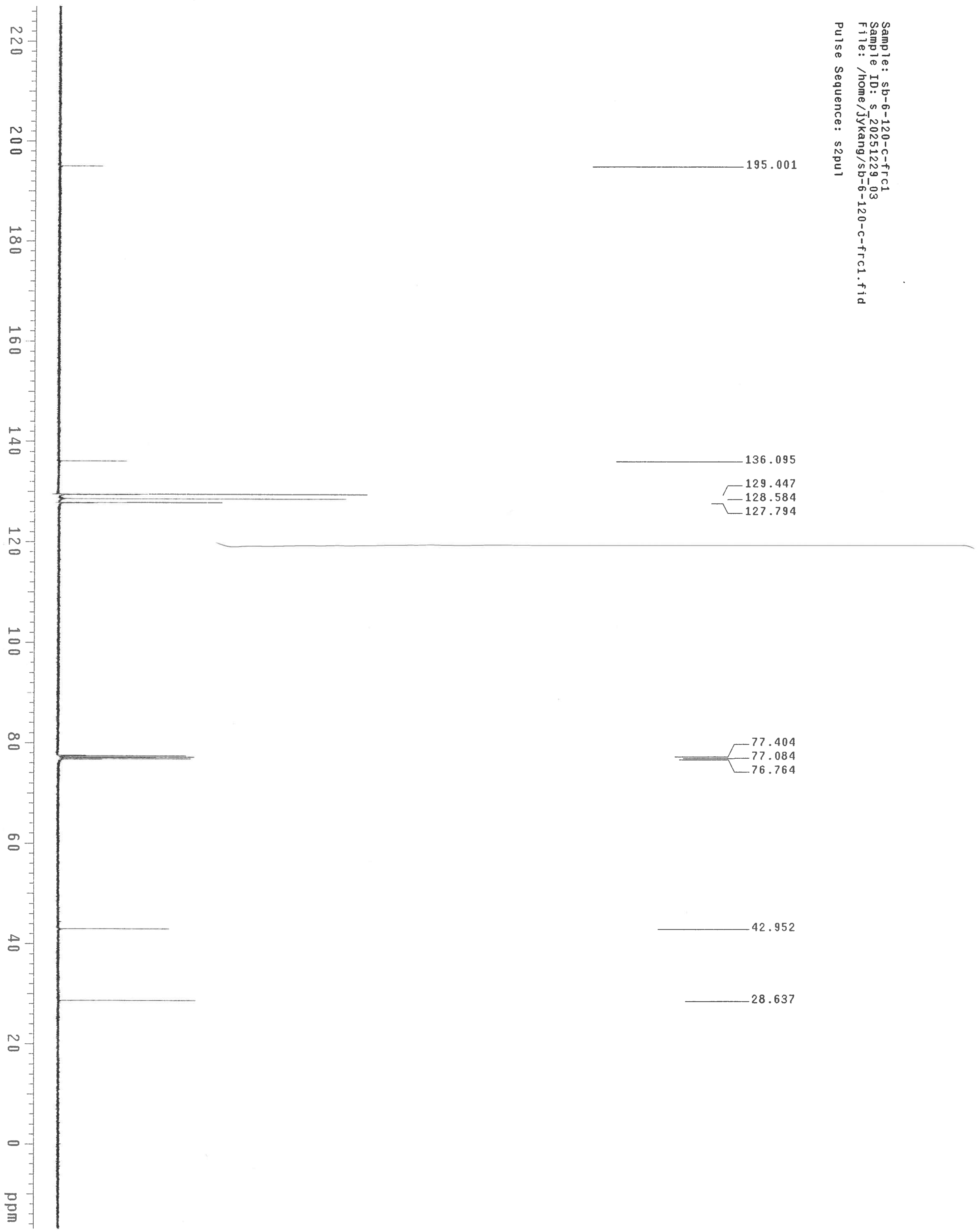

^{1}H NMR (400 MHz) in $CDCl_3$

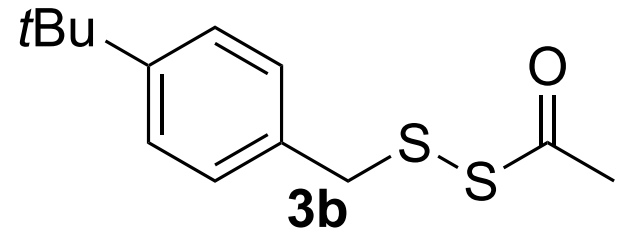

3b

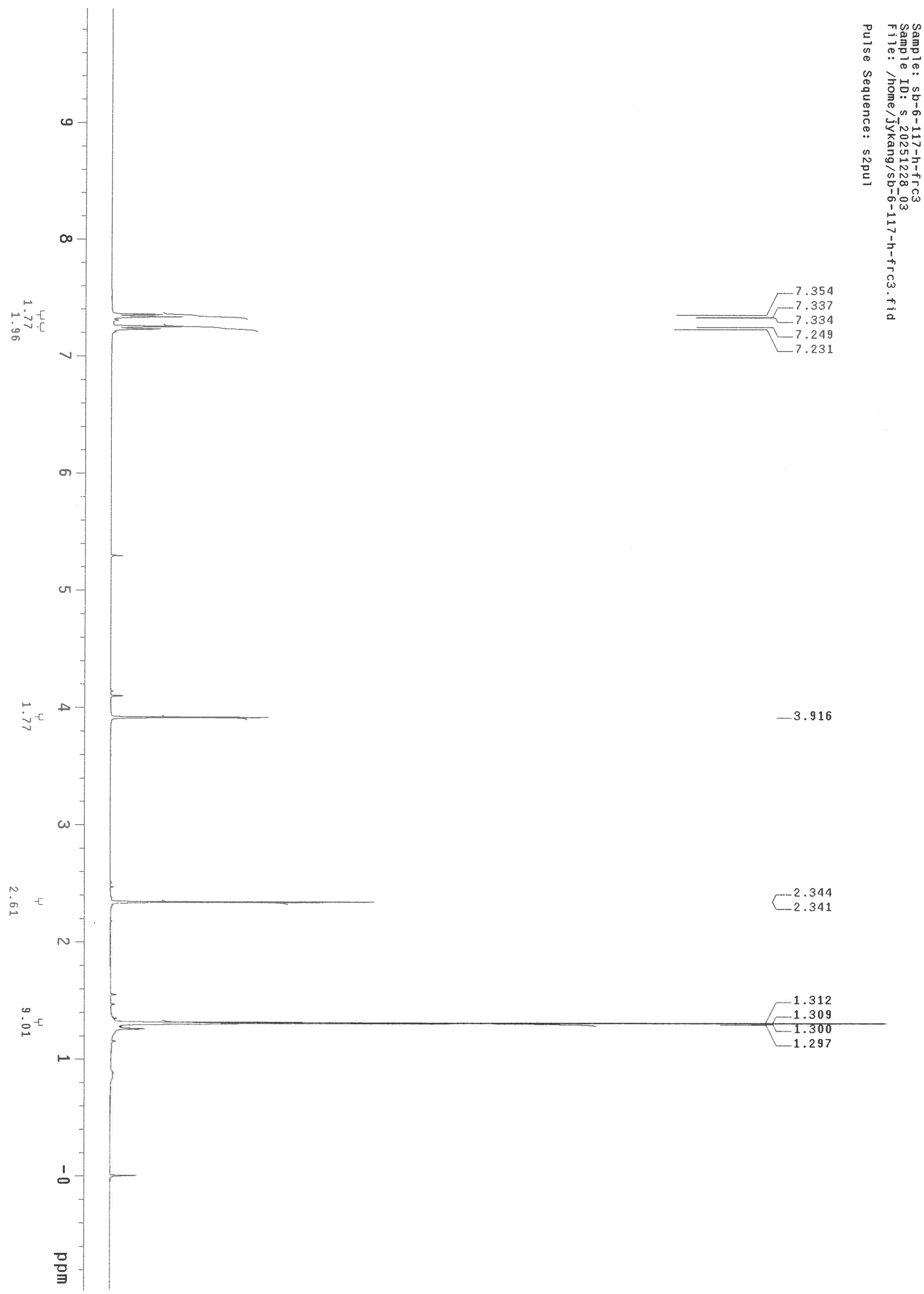

^{13}C NMR (100.5 MHz) in $CDCl_3$

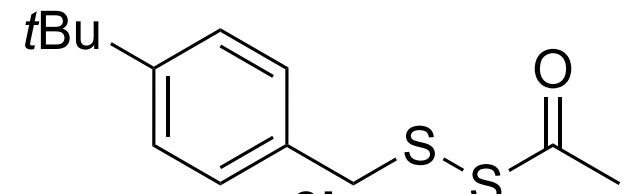

3b

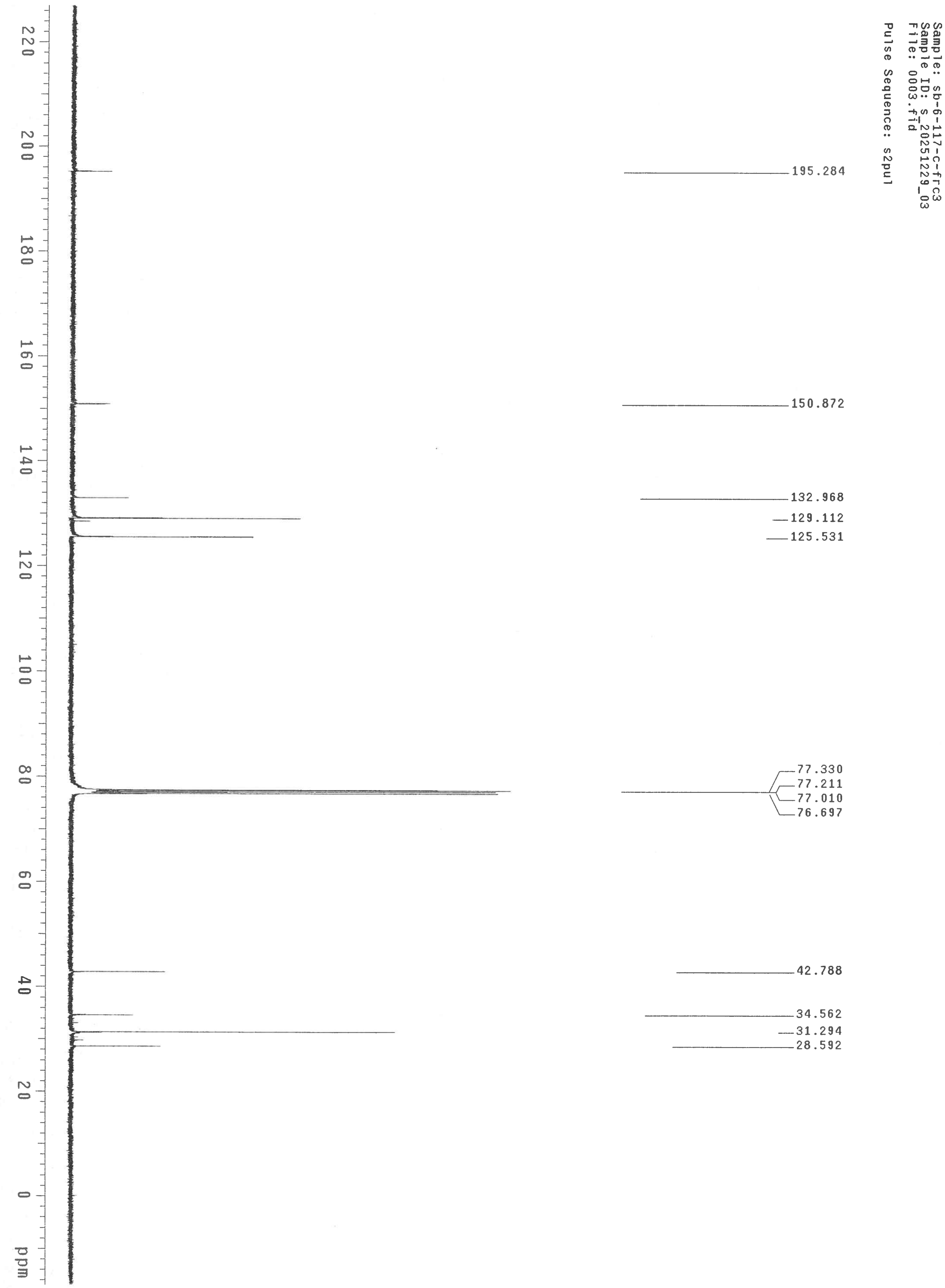

^{1}H NMR (400 MHz) in $CDCl_3$

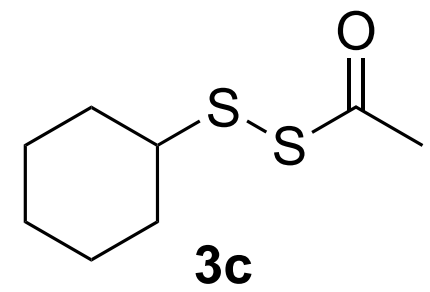

3c

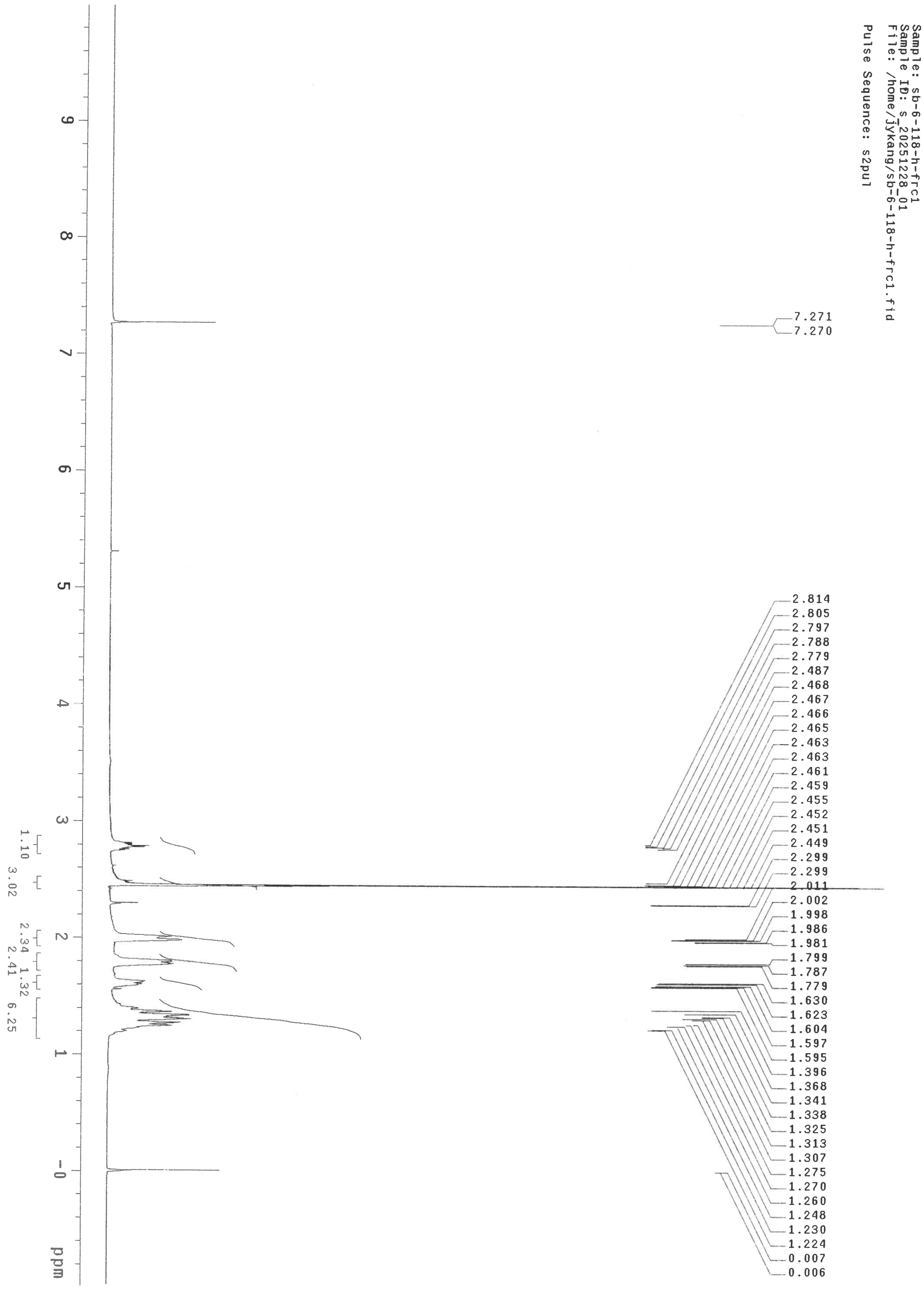

^{13}C NMR (100.5 MHz) in $CDCl_3$

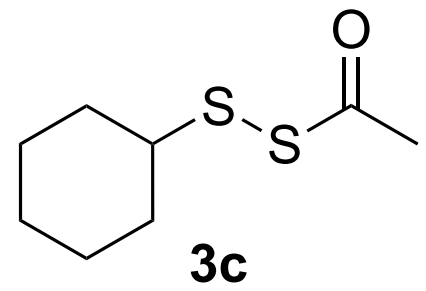

3c

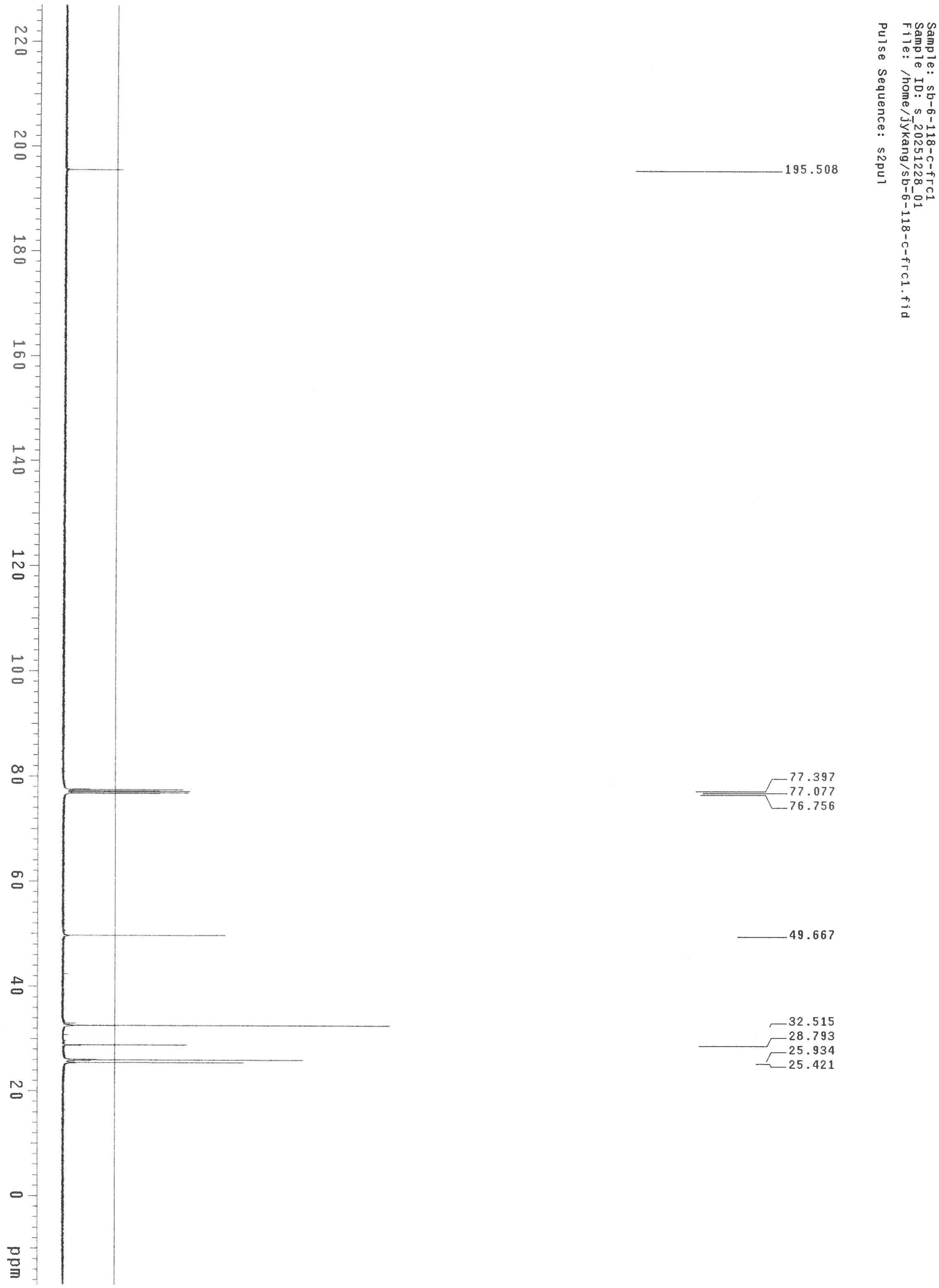

^{1}H NMR (400 MHz) in $CDCl_3$

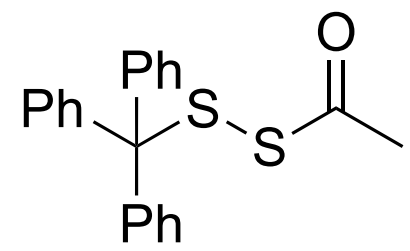

3d

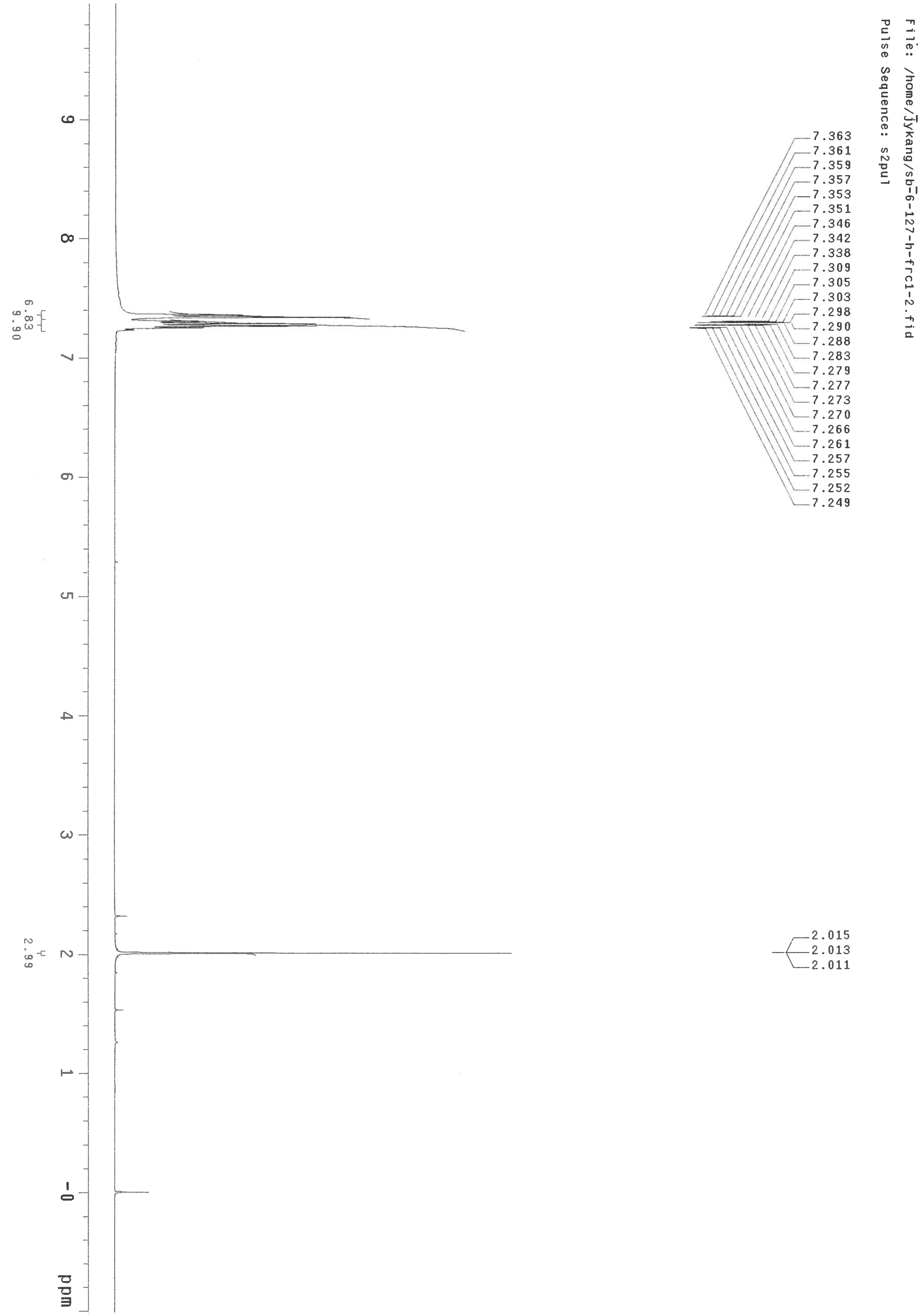

^{13}C NMR (100.5 MHz) in $CDCl_3$

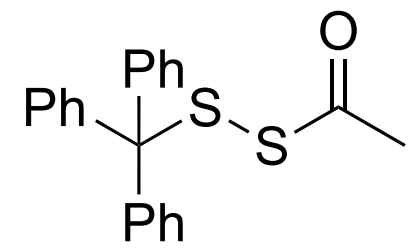

3d

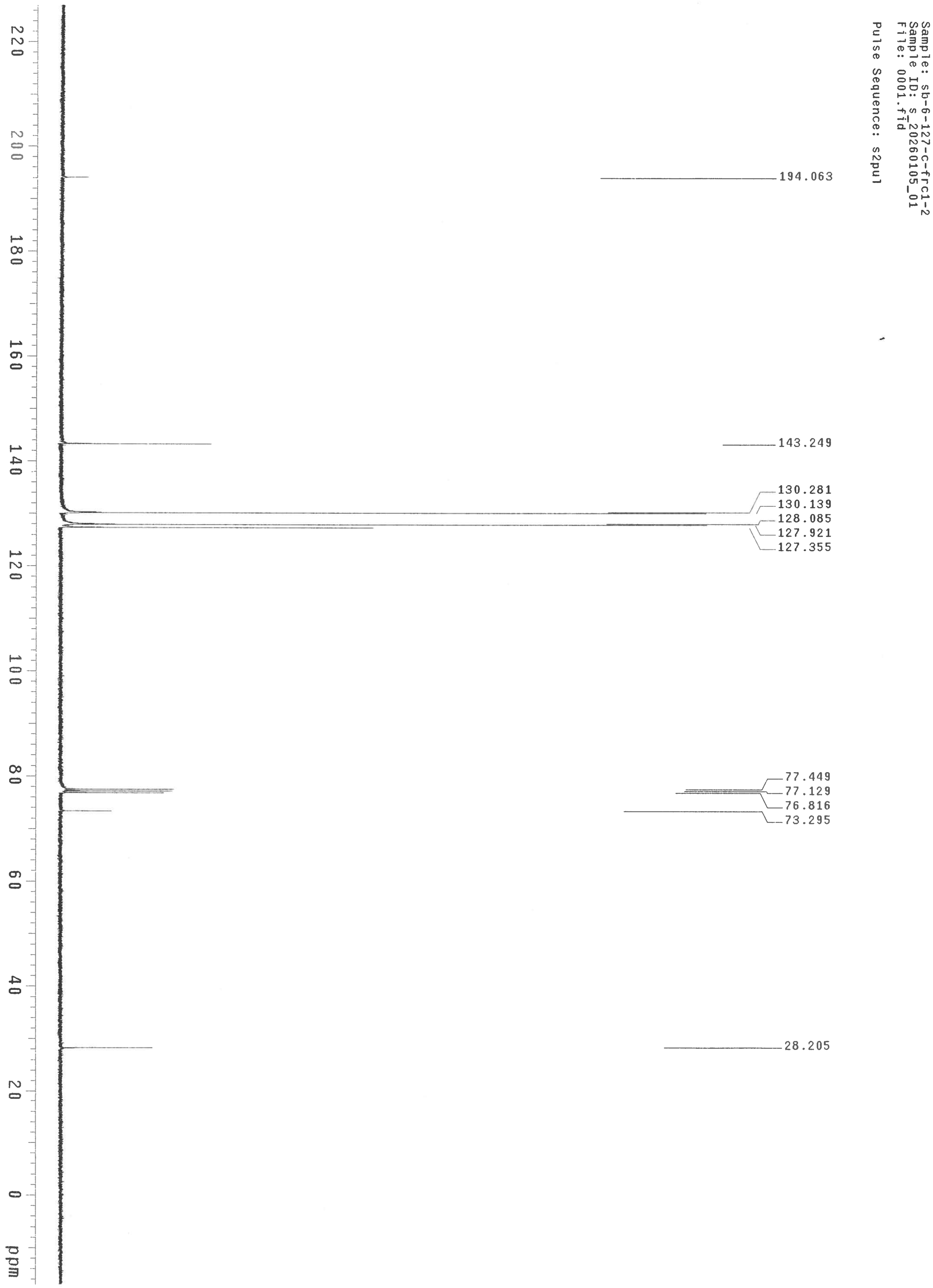

^{1}H NMR (400 MHz) in $CDCl_3$

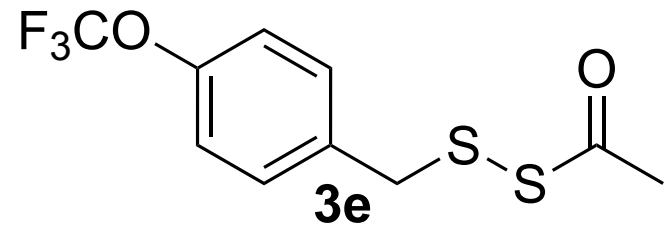

3e

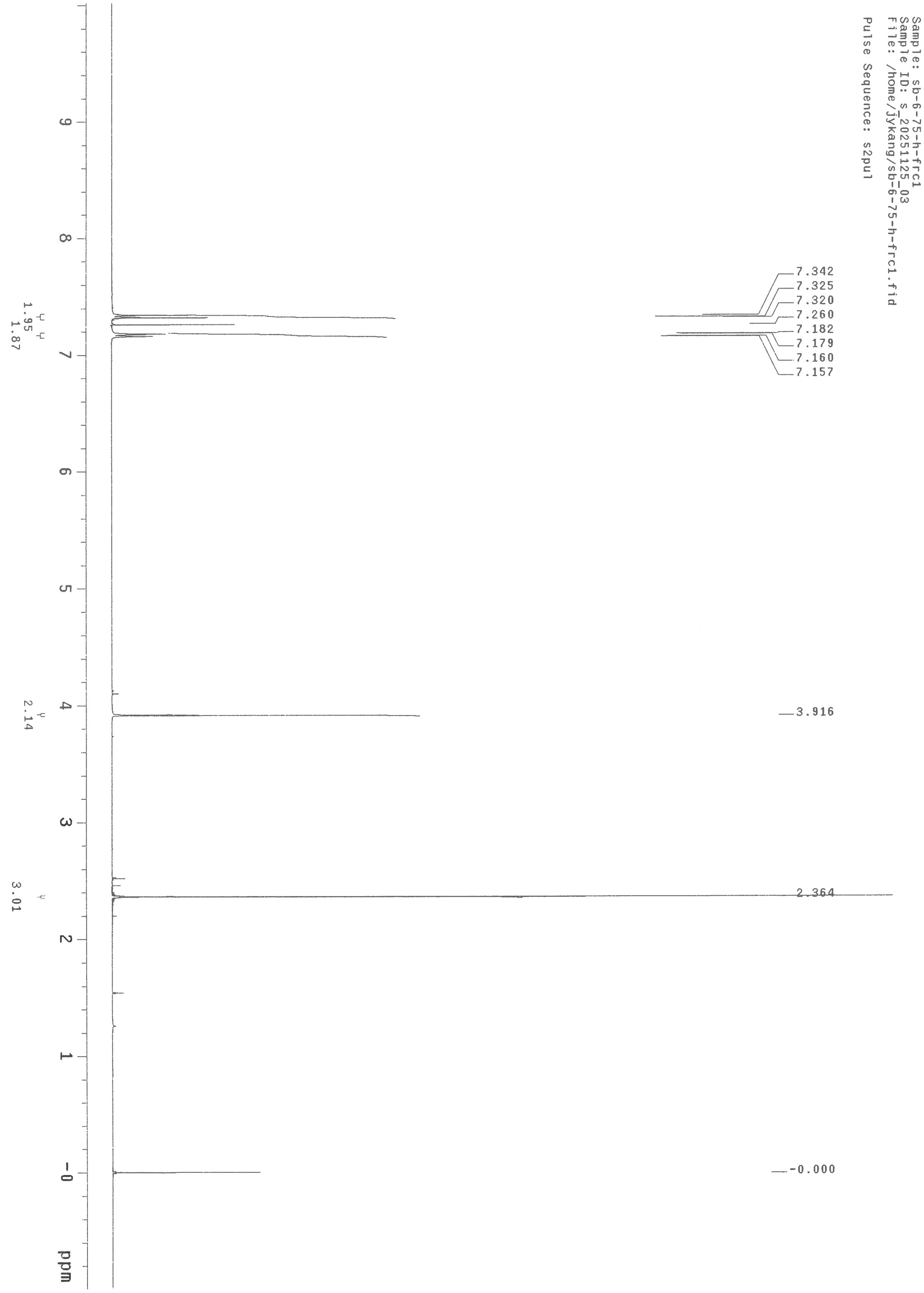

^{13}C NMR (100.5 MHz) in $CDCl_3$

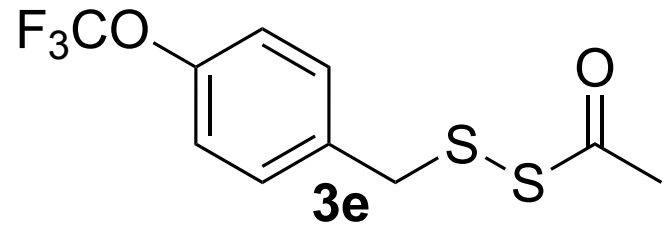

3e

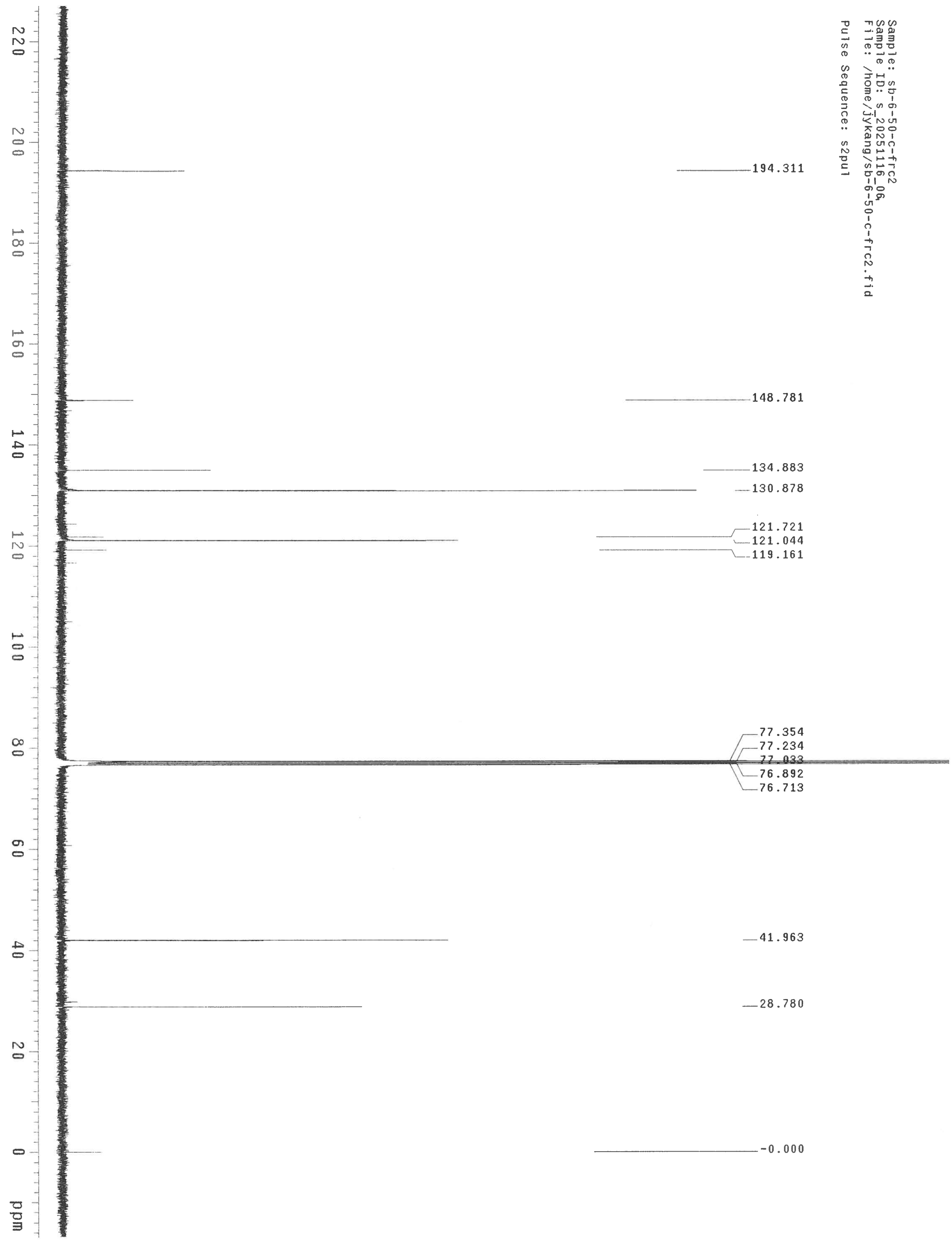

^{19}F NMR (375.9 MHz) in $CDCl_3$

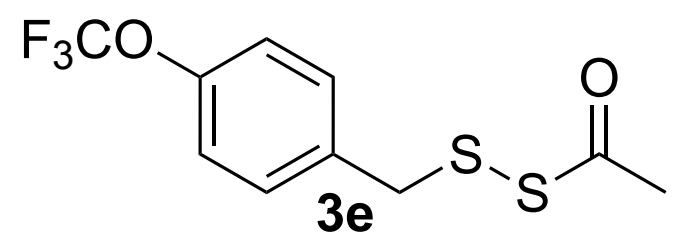

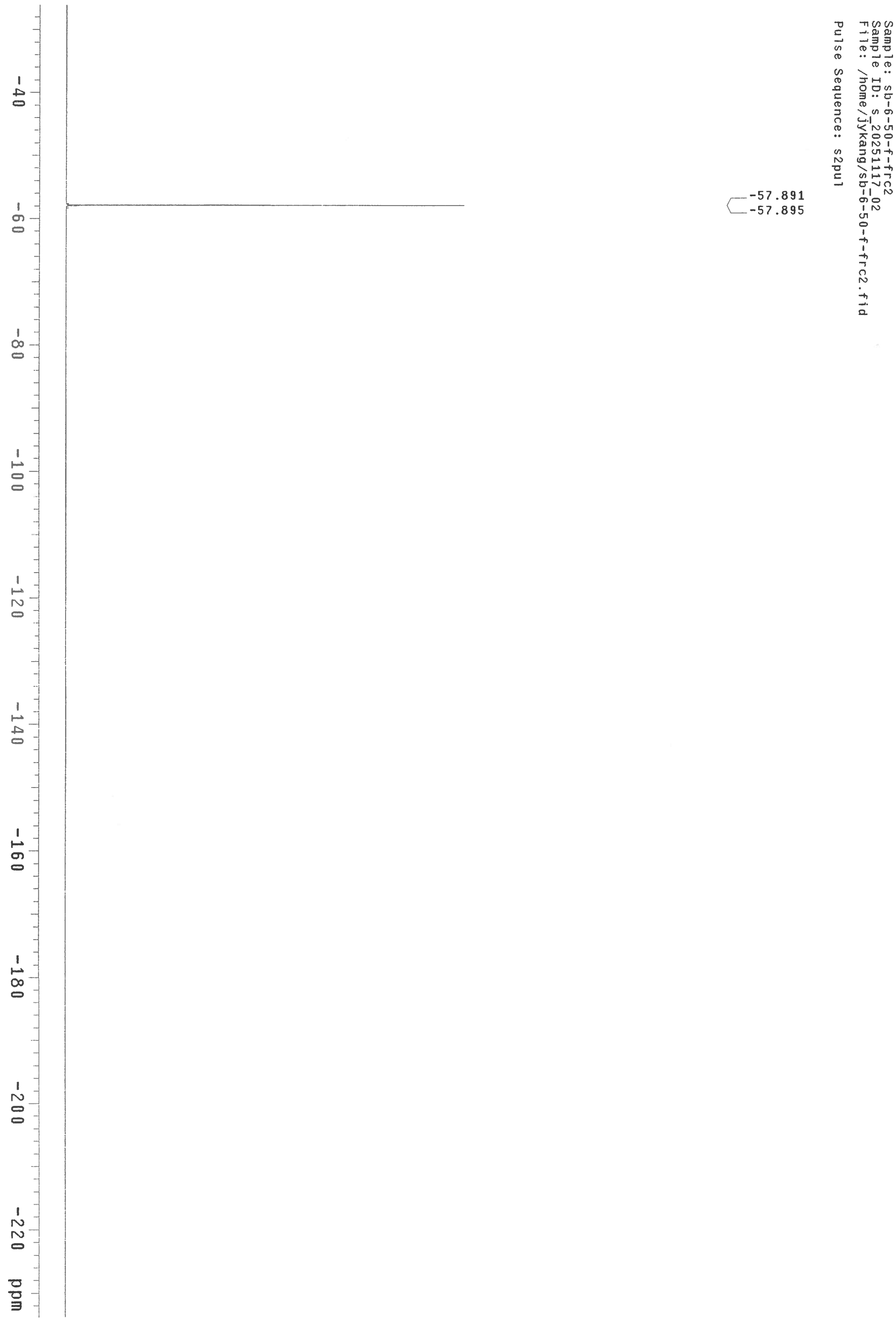

^{1}H NMR (400 MHz) in $CDCl_3$

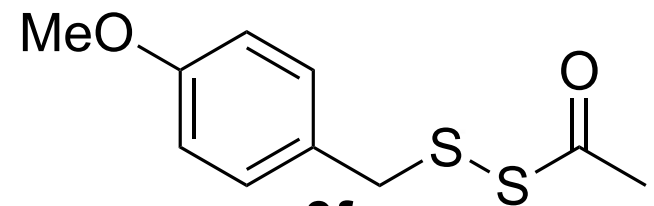

3f

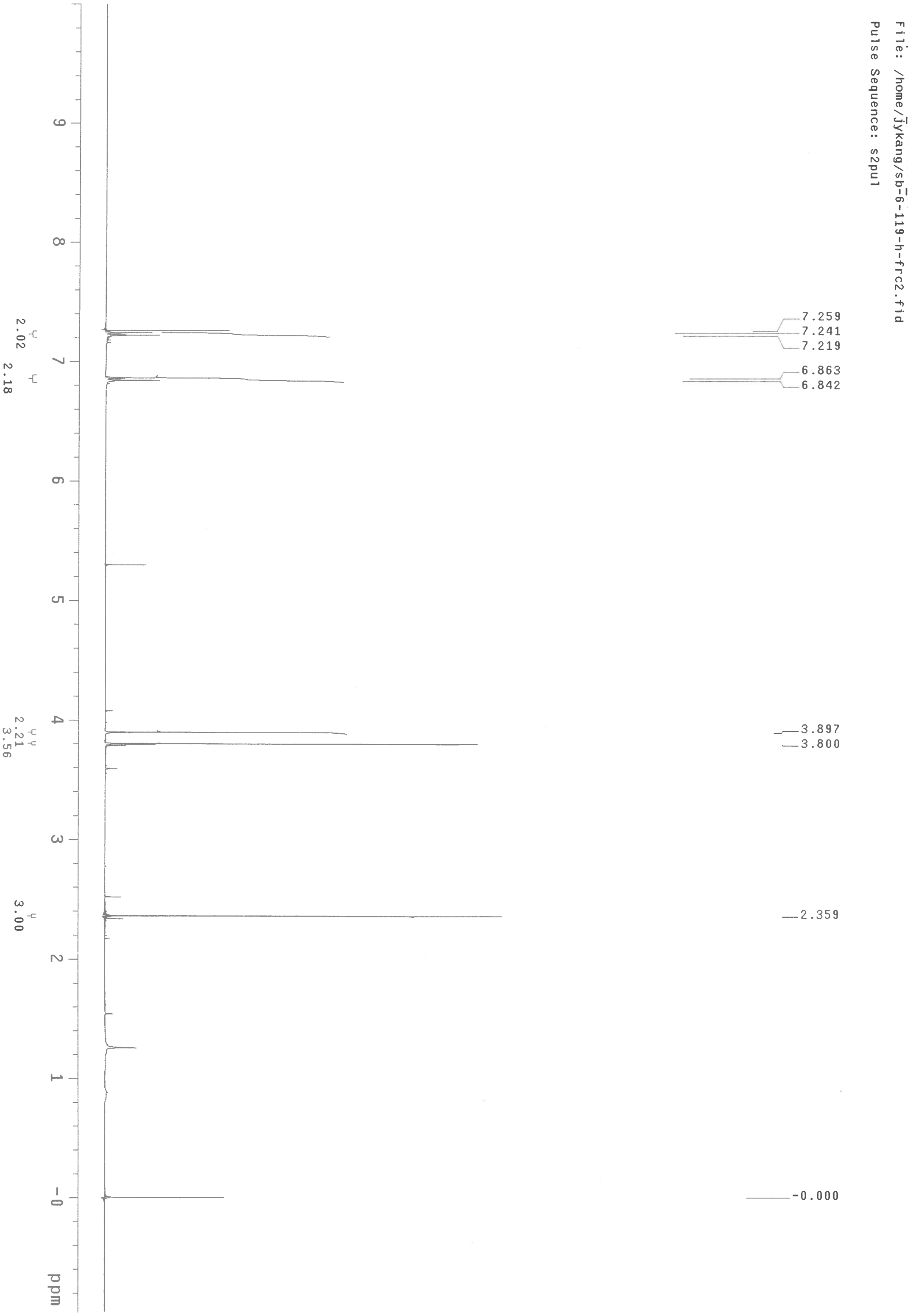

^{13}C NMR (100.5 MHz) in $CDCl_3$

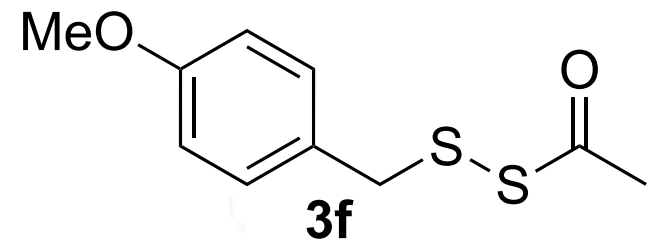

3f

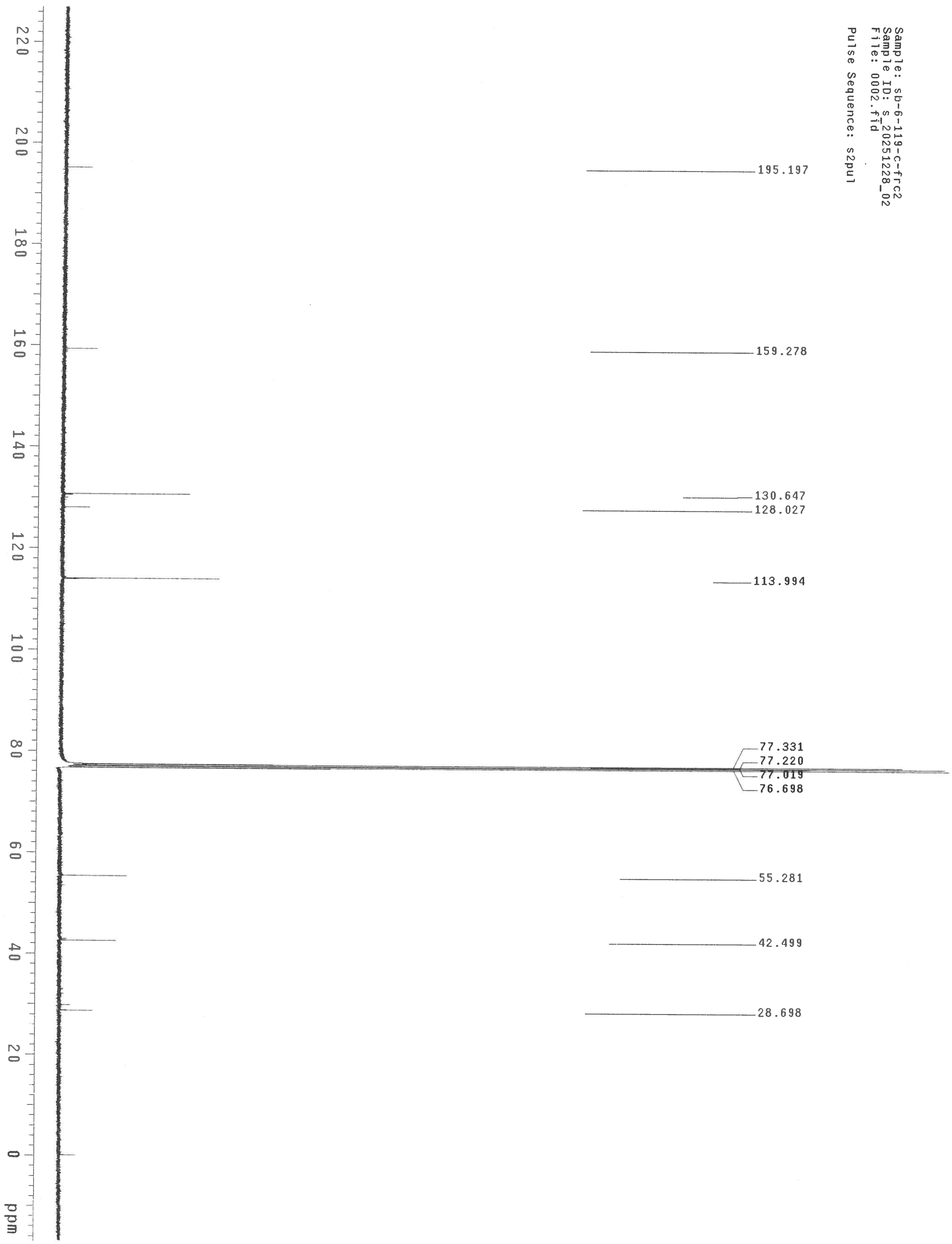

^{1}H NMR (400 MHz) in $CDCl_3$

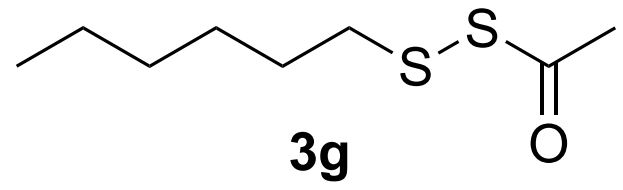

3g

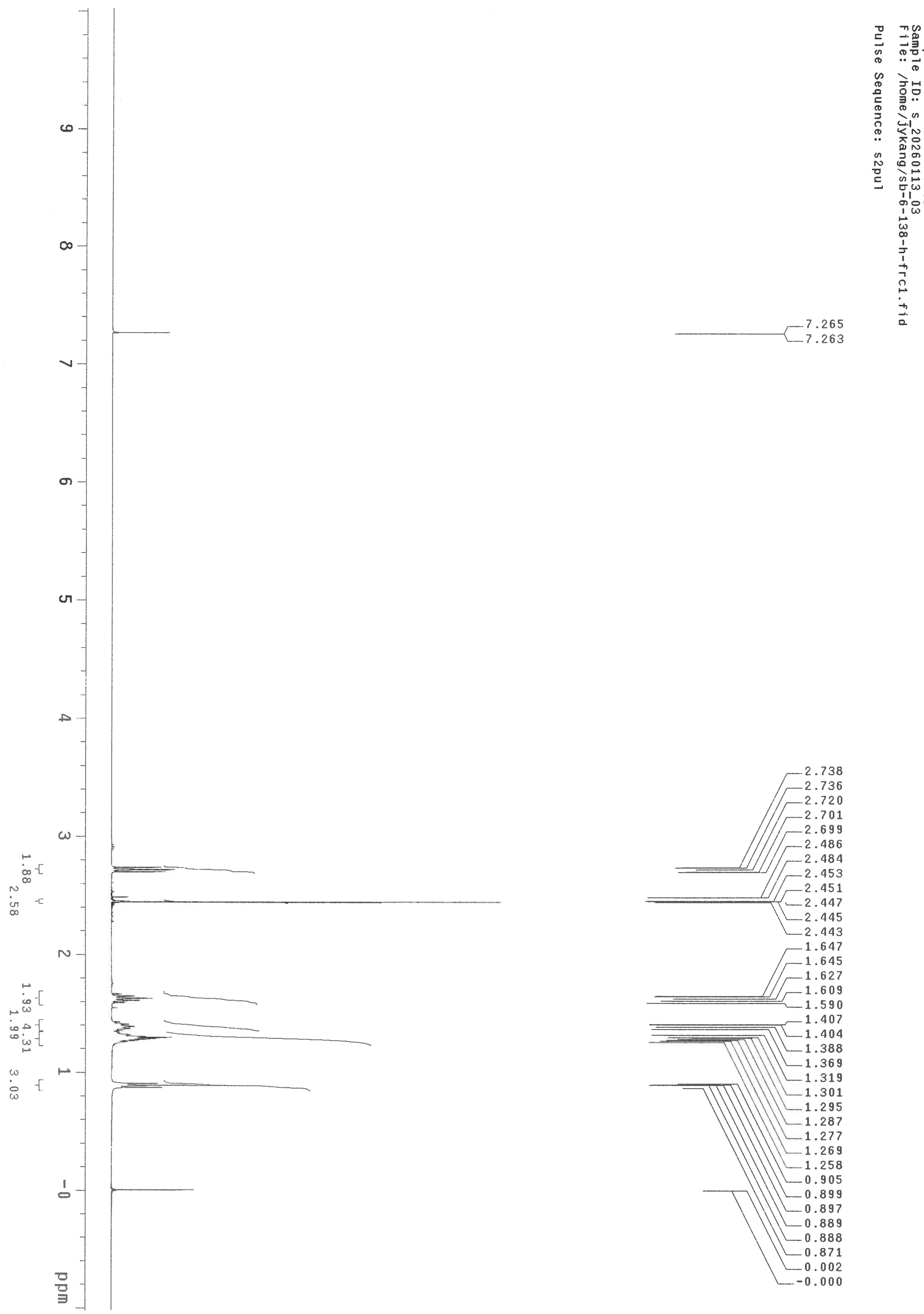

^{13}C NMR (100.5 MHz) in $CDCl_3$

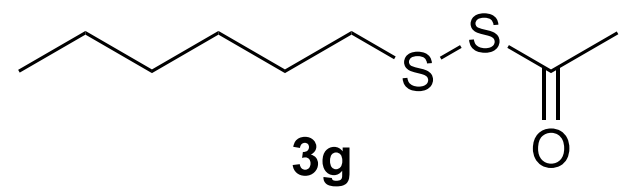

3g

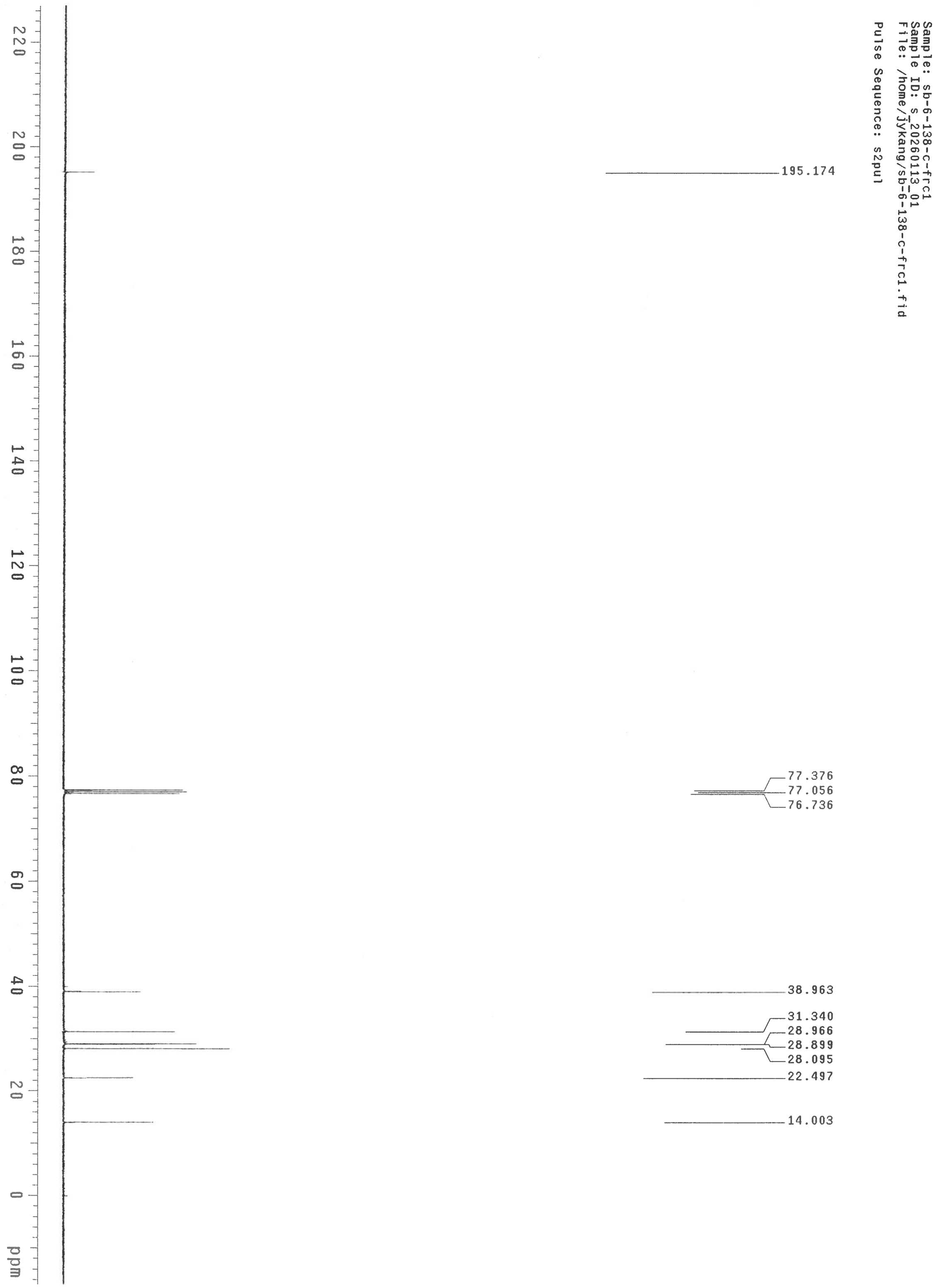

^{1}H NMR (400 MHz) in $CDCl_3$

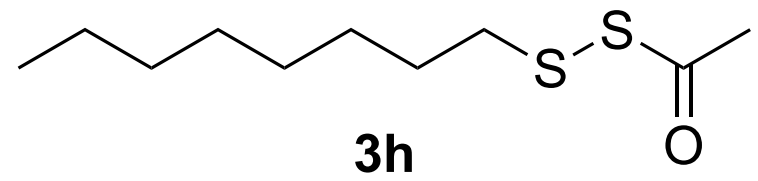

3h

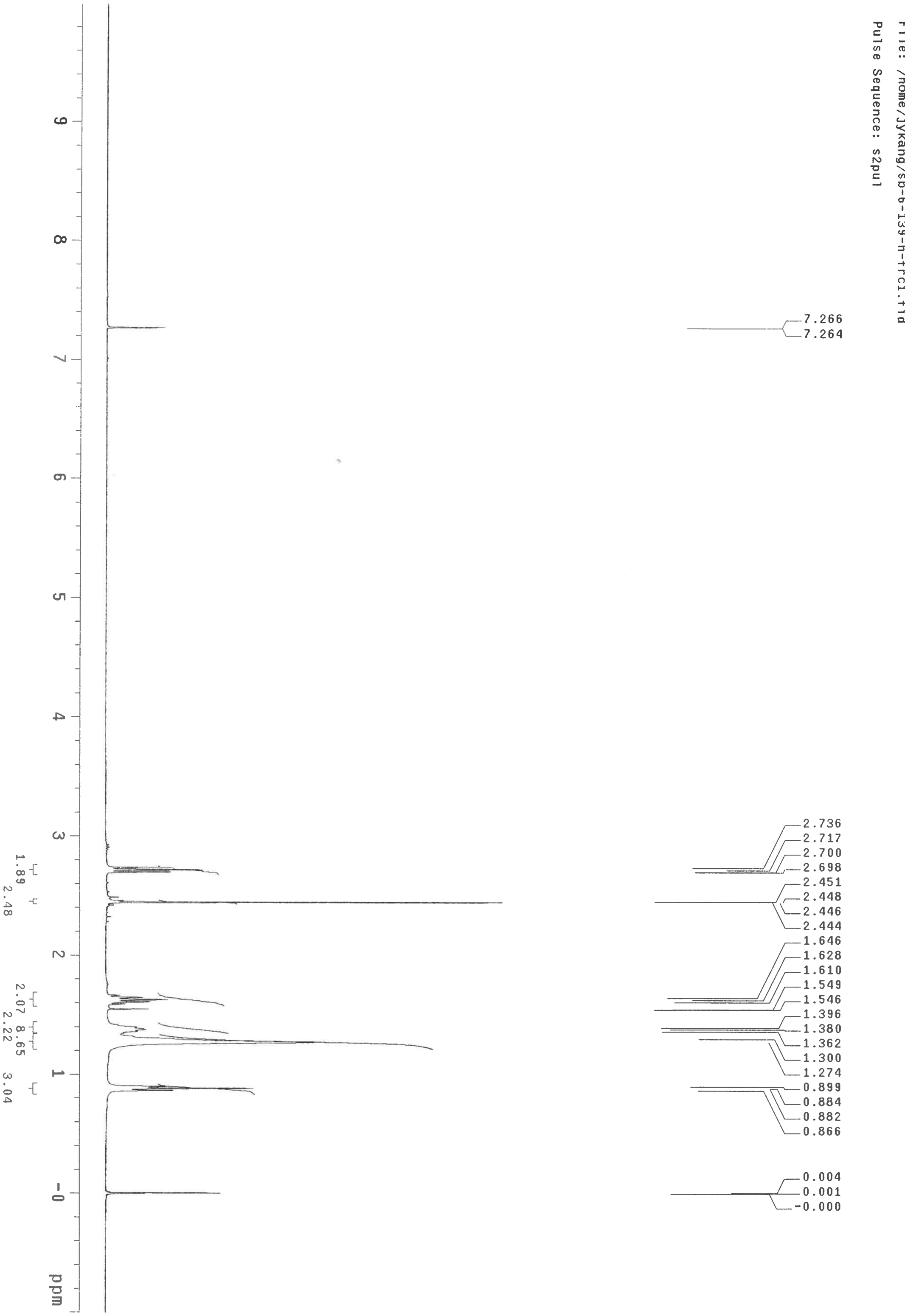

^{13}C NMR (100.5 MHz) in $CDCl_3$

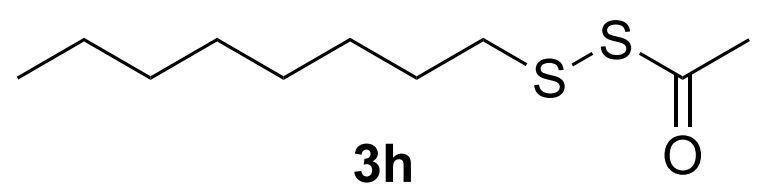

3h

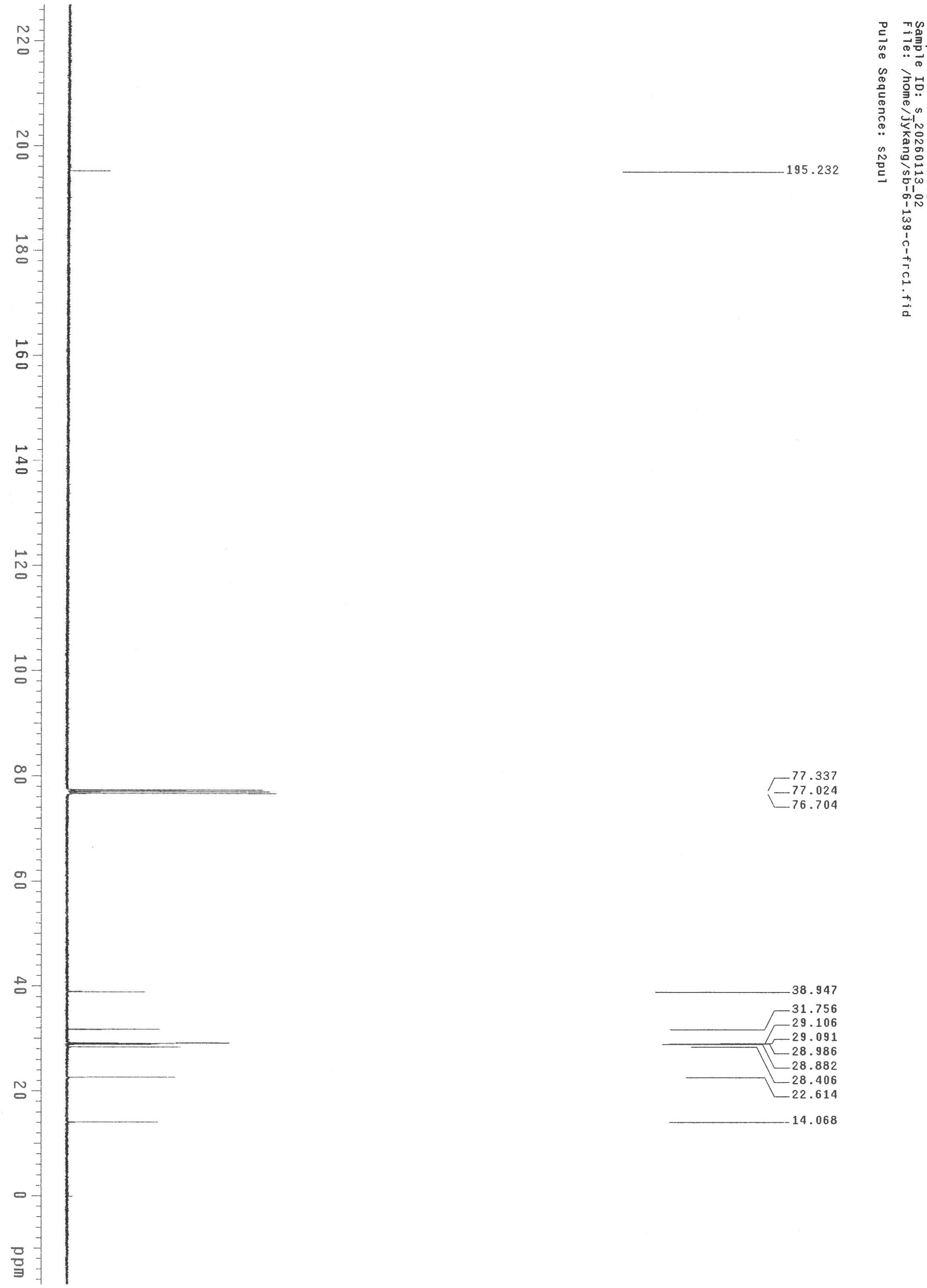

^{1}H NMR (400 MHz) in $CDCl_3$

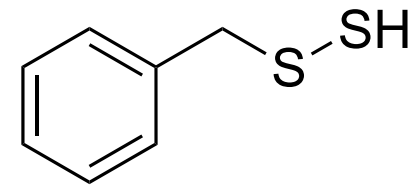

4a

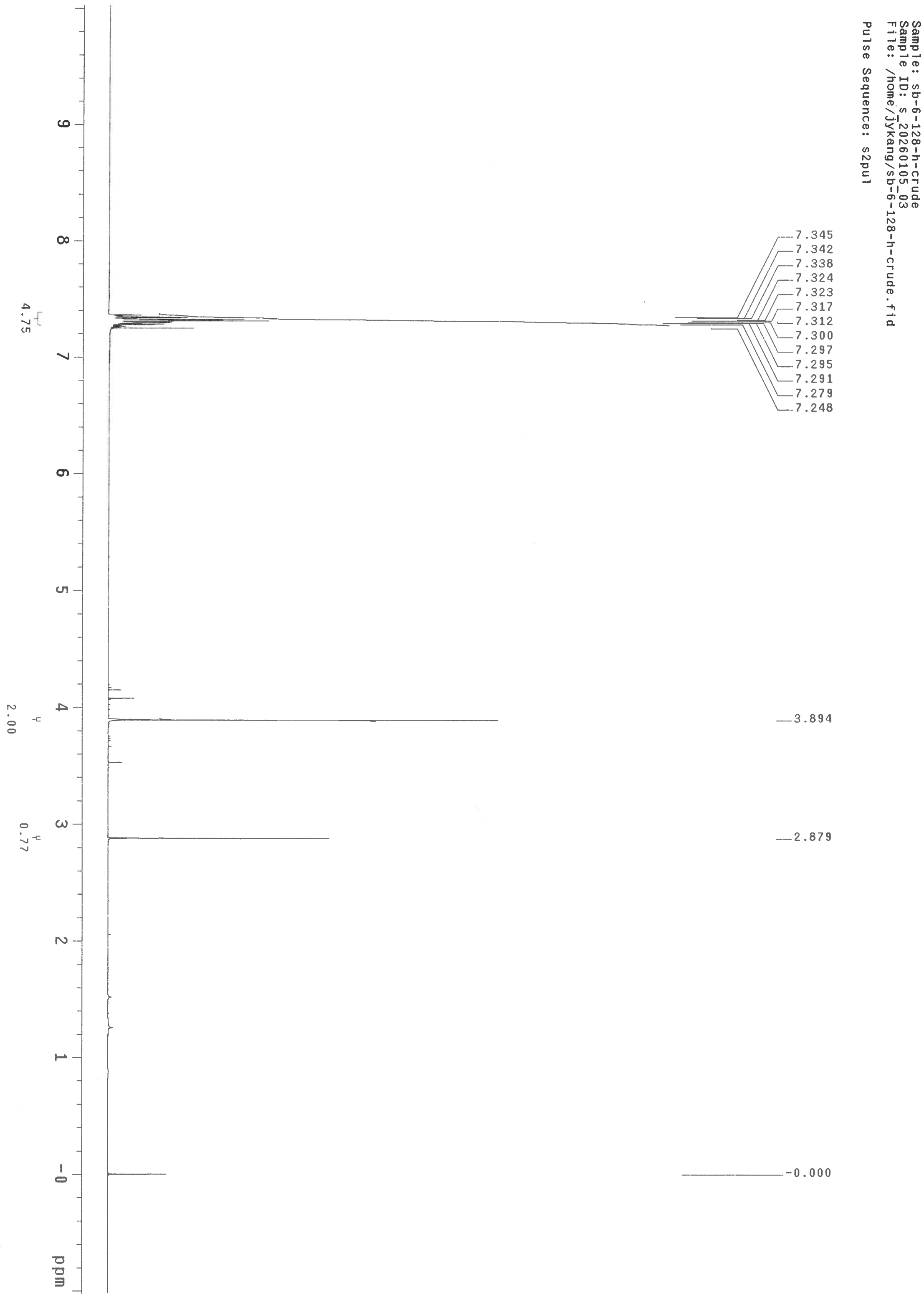

^{13}C NMR (100.5 MHz) in $CDCl_3$

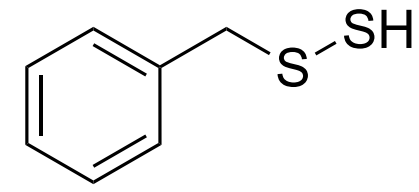

4a

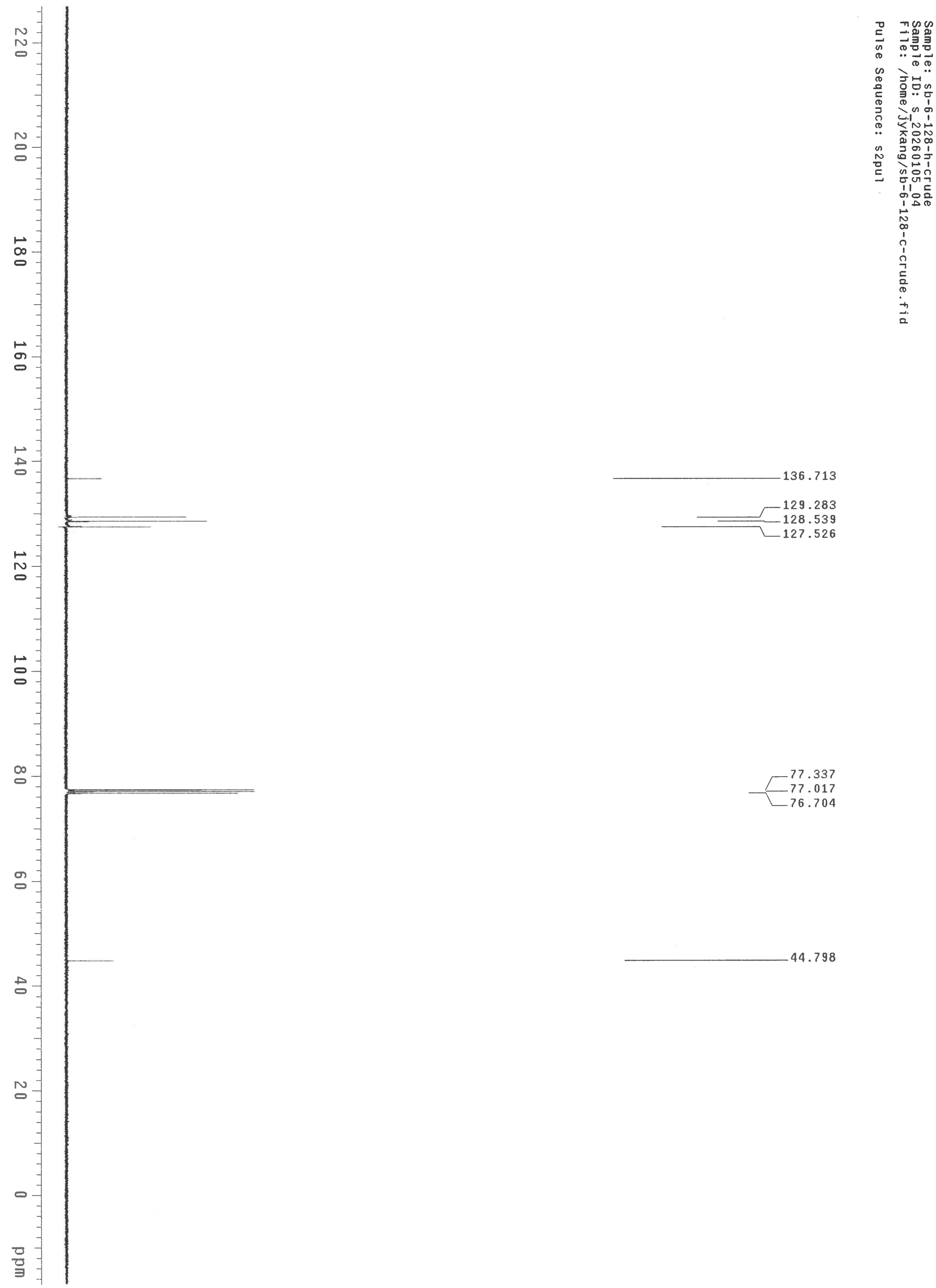

^{1}H NMR (400 MHz) in $CDCl_3$

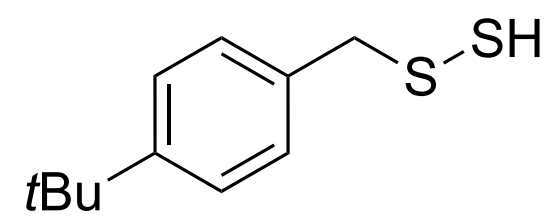

4b

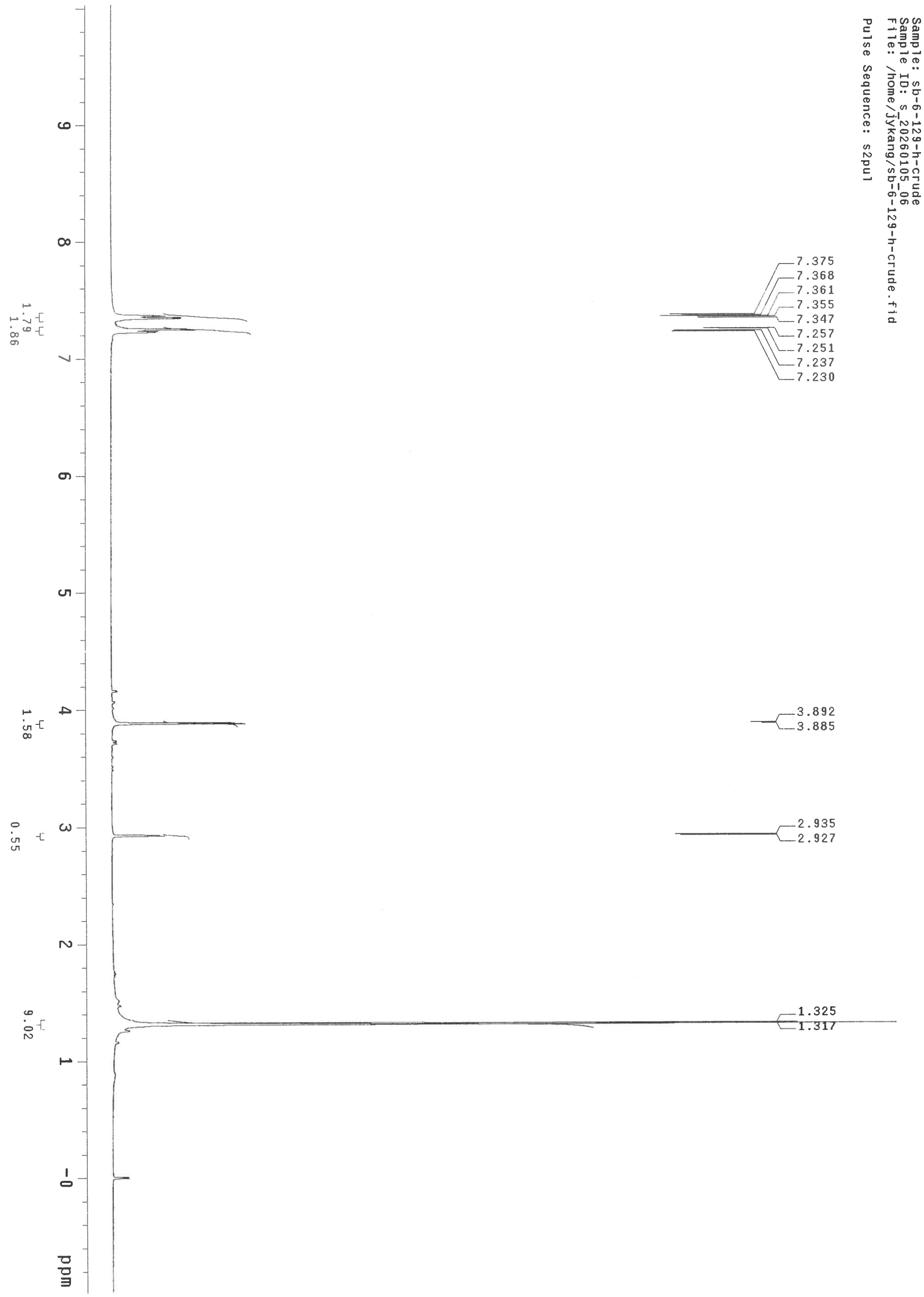

^{13}C NMR (100.5 MHz) in $CDCl_3$

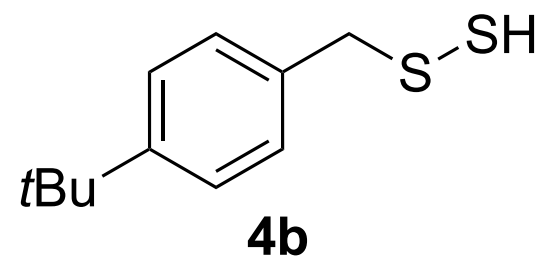

4b

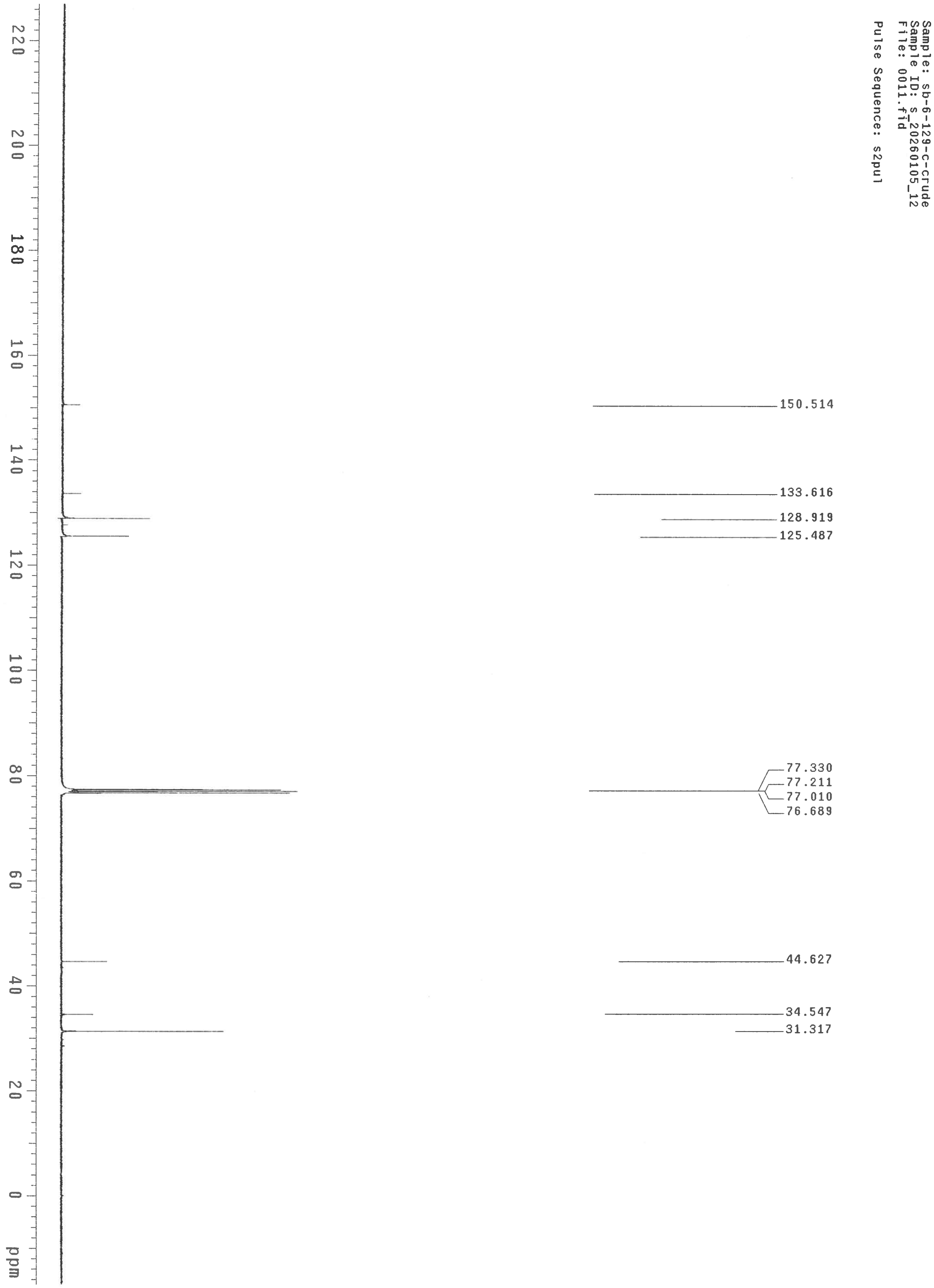

^{1}H NMR (400 MHz) in $CDCl_3$

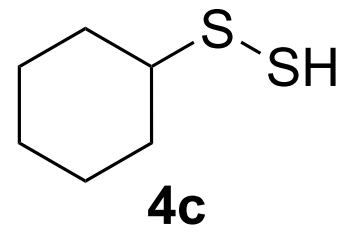

4c

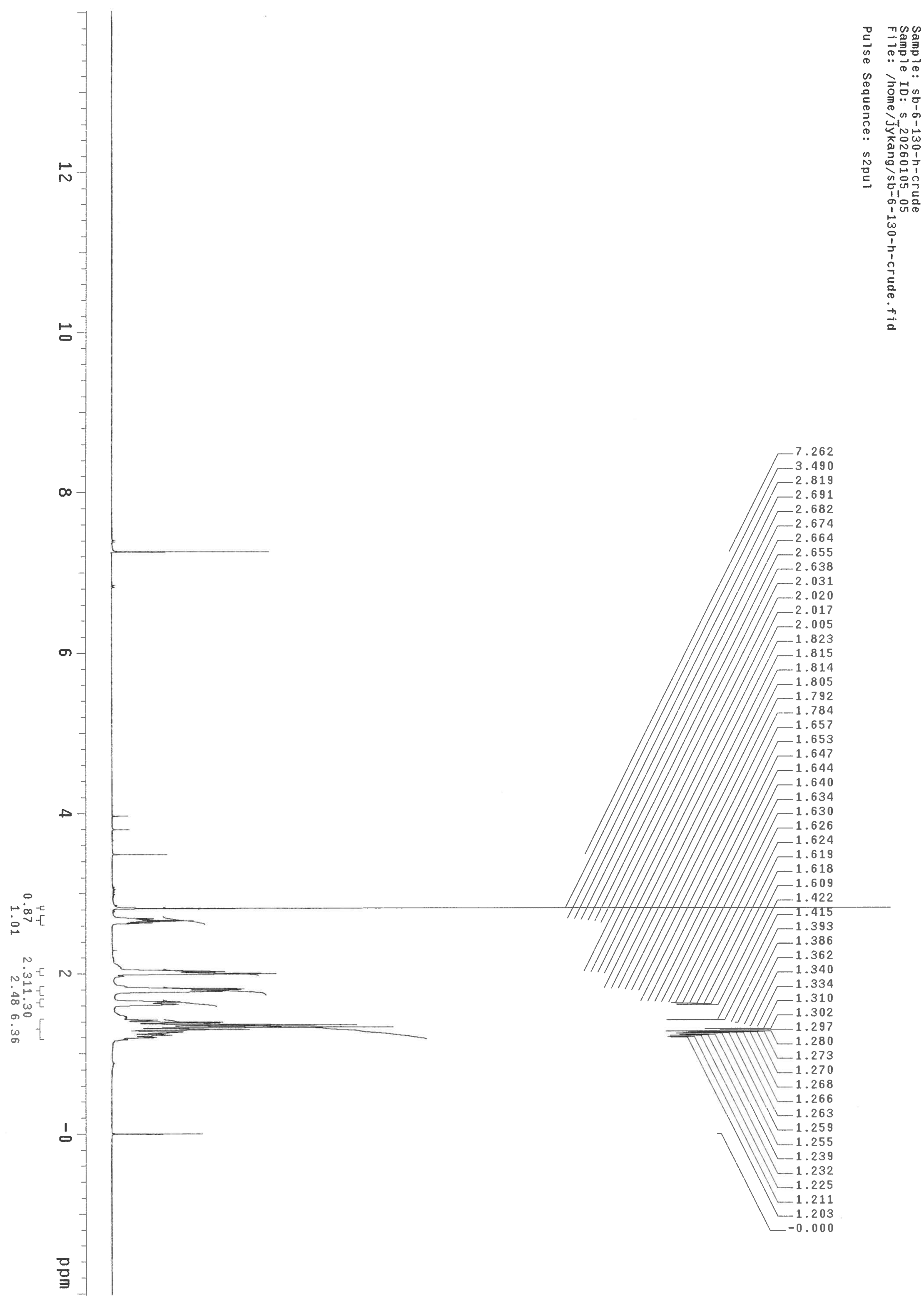

^{13}C NMR (100.5 MHz) in $CDCl_3$

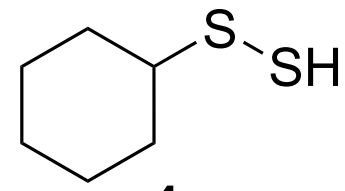

4c

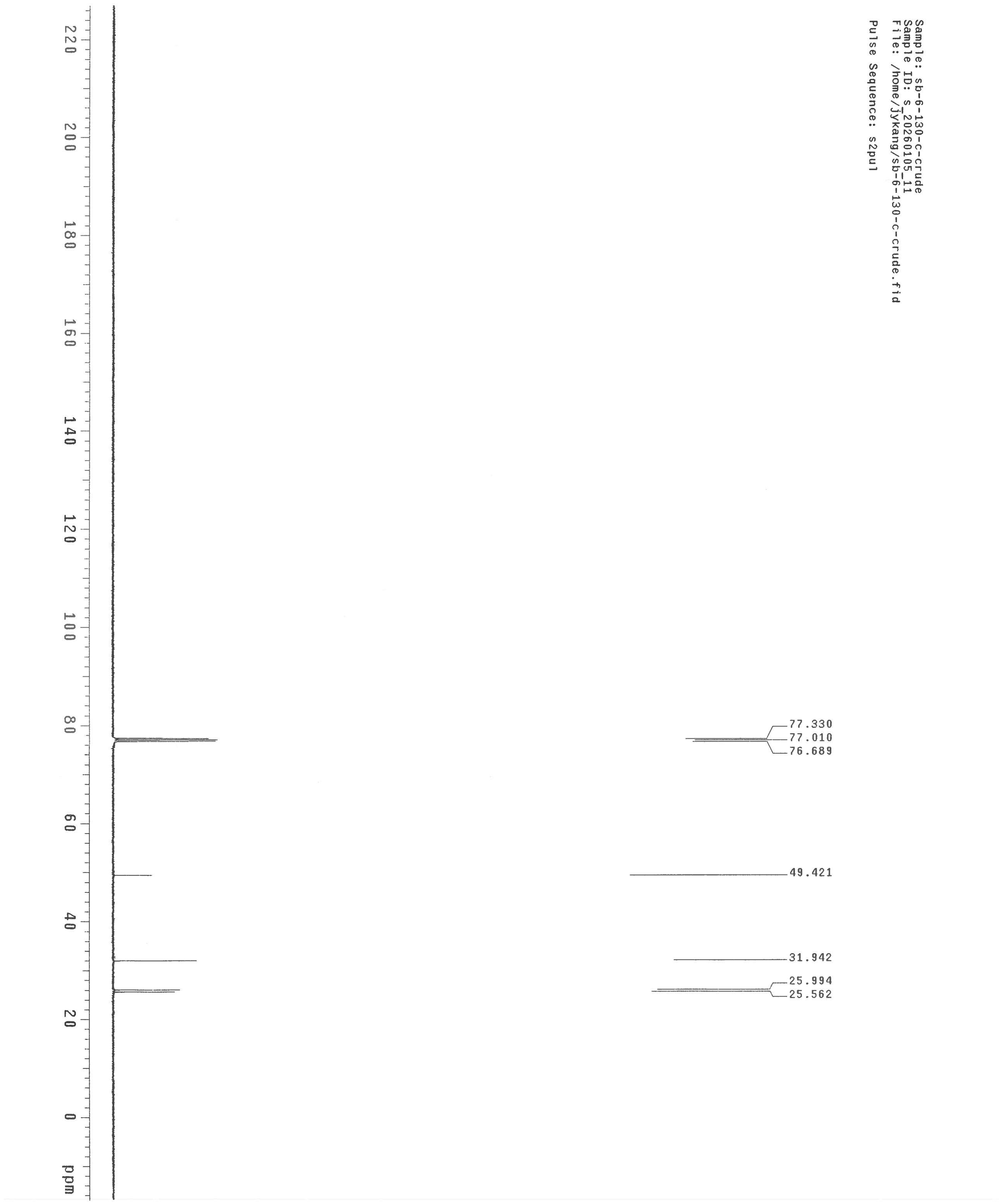

^{1}H NMR (400 MHz) in $CDCl_3$

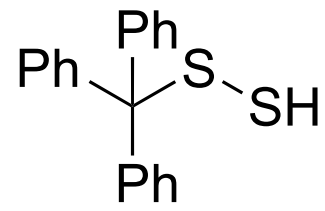

4d

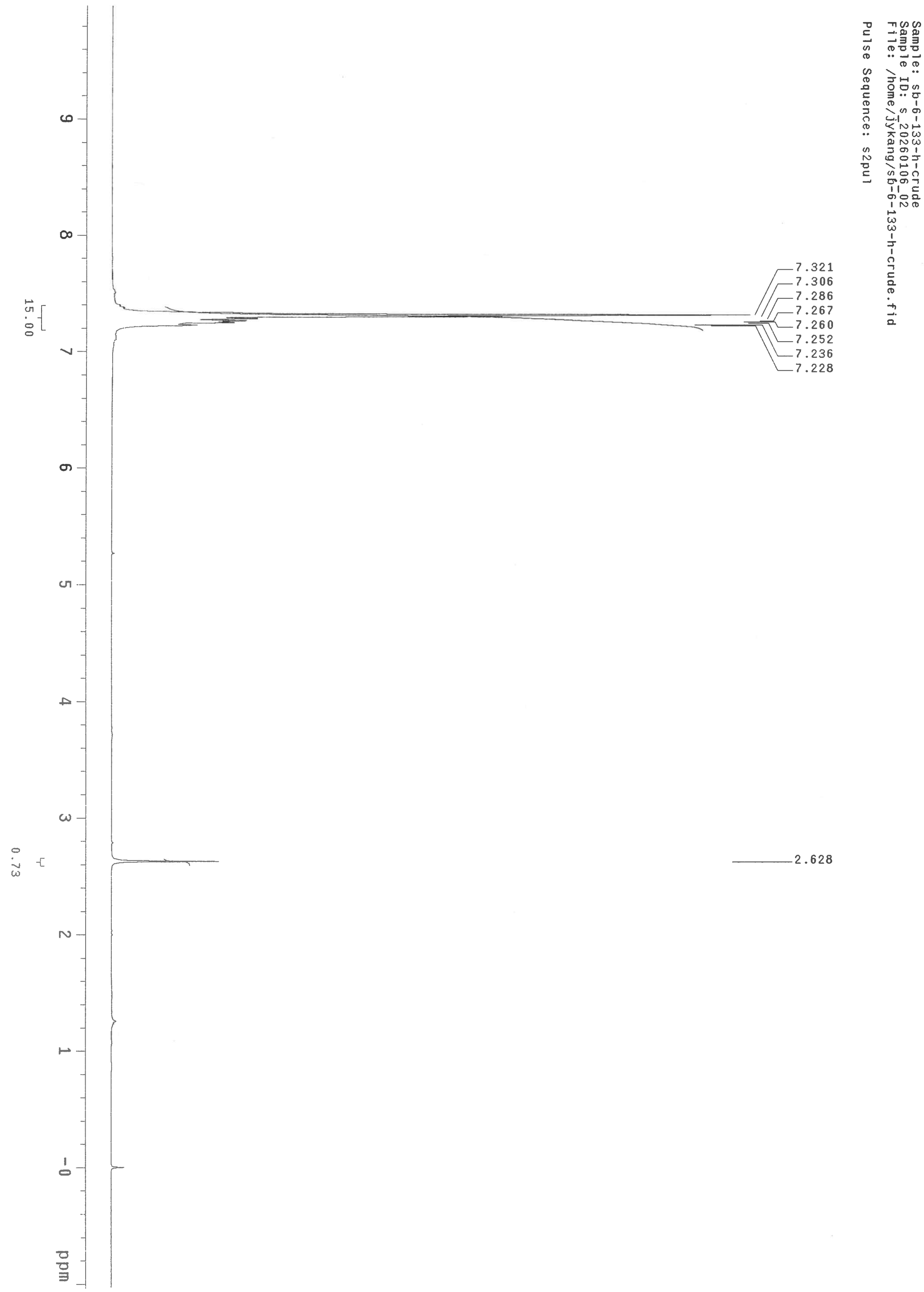

^{13}C NMR (100.5 MHz) in $CDCl_3$

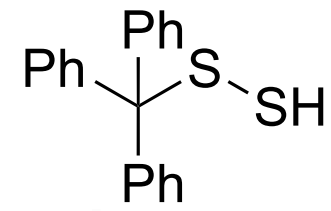

4d

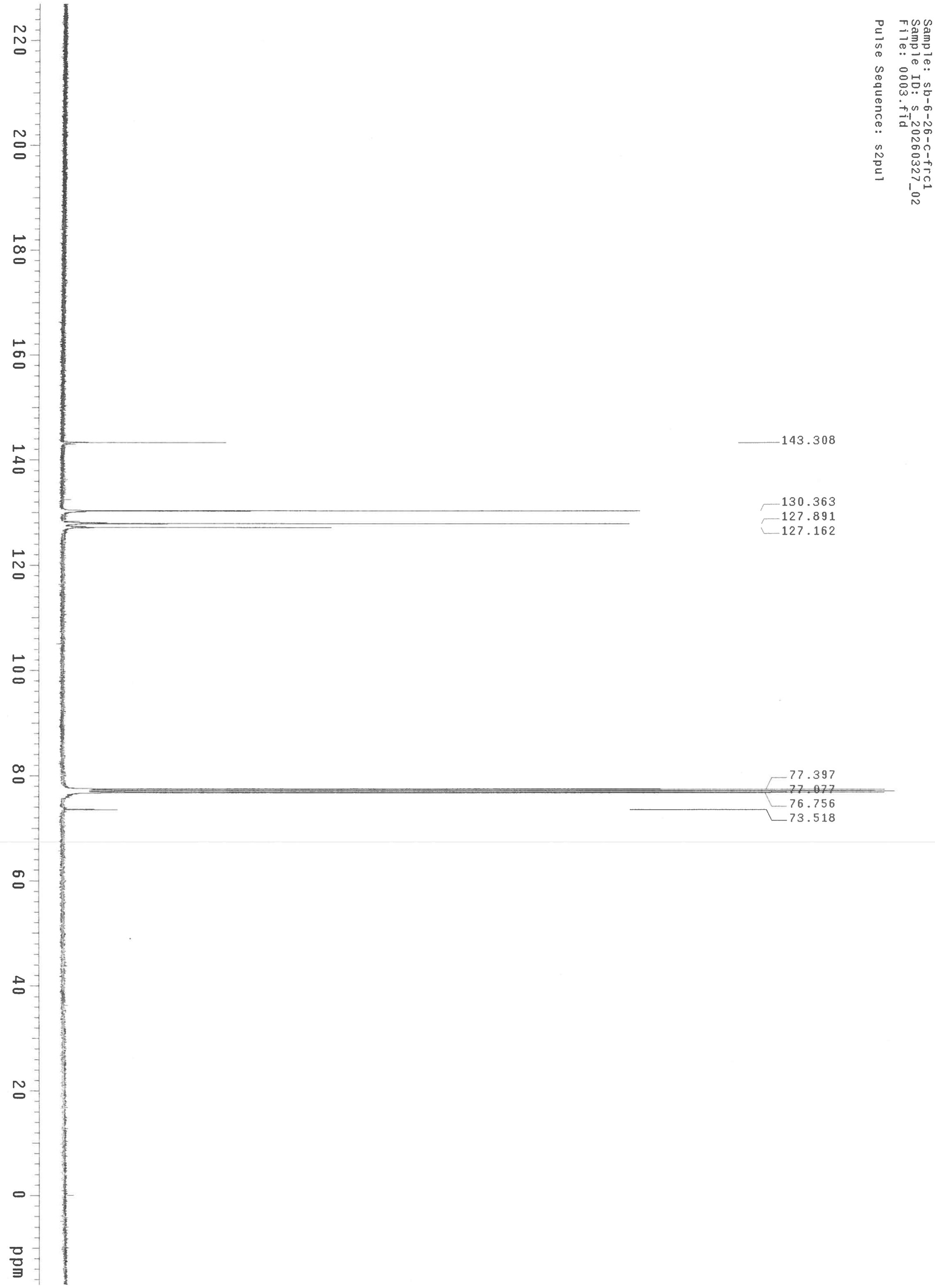

^{1}H NMR (400 MHz) in $CDCl_3$

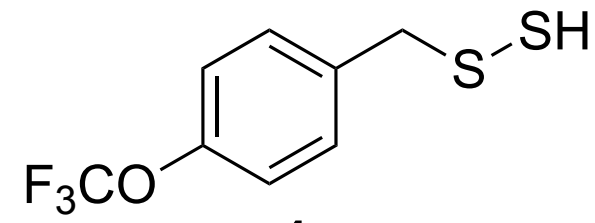

4e

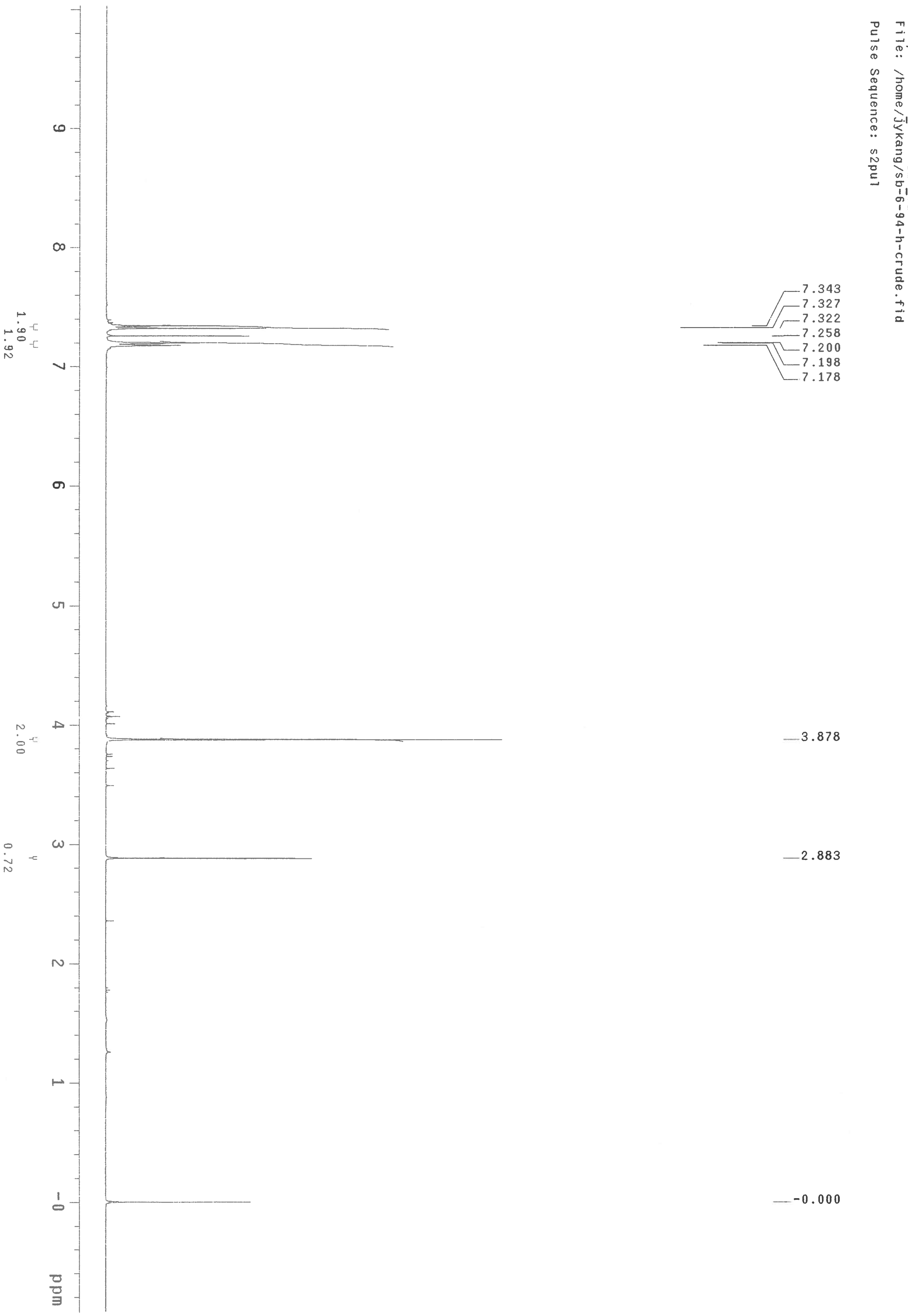

^{13}C NMR (100.5 MHz) in $CDCl_3$

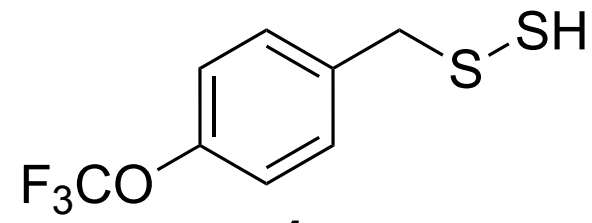

4e

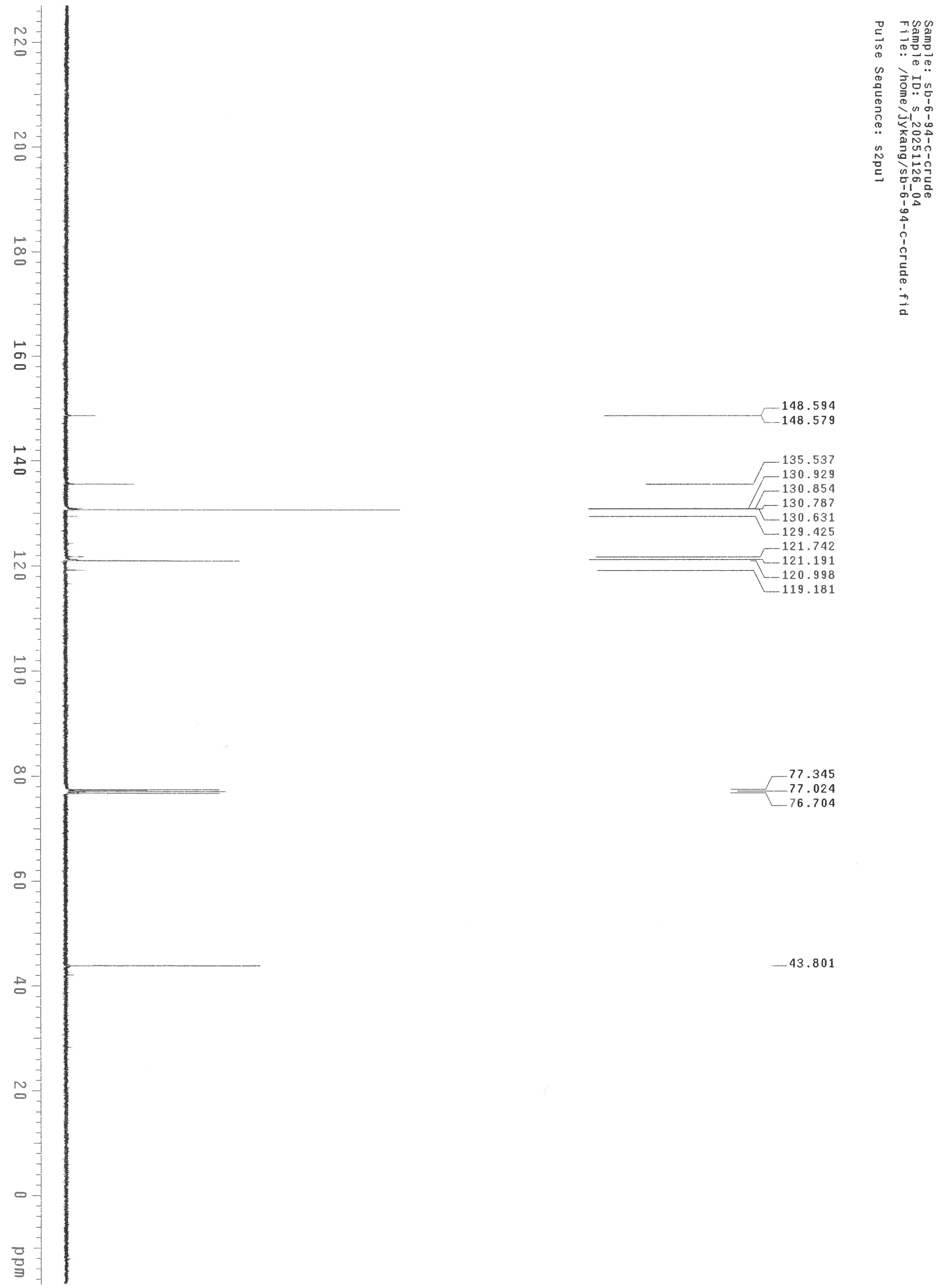

^{19}F NMR (375.9 MHz) in $CDCl_3$

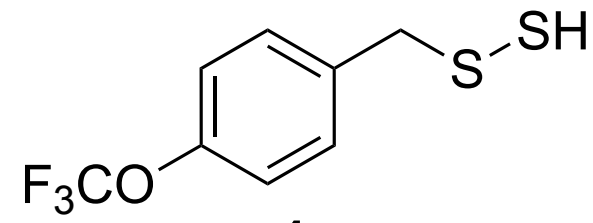

4e

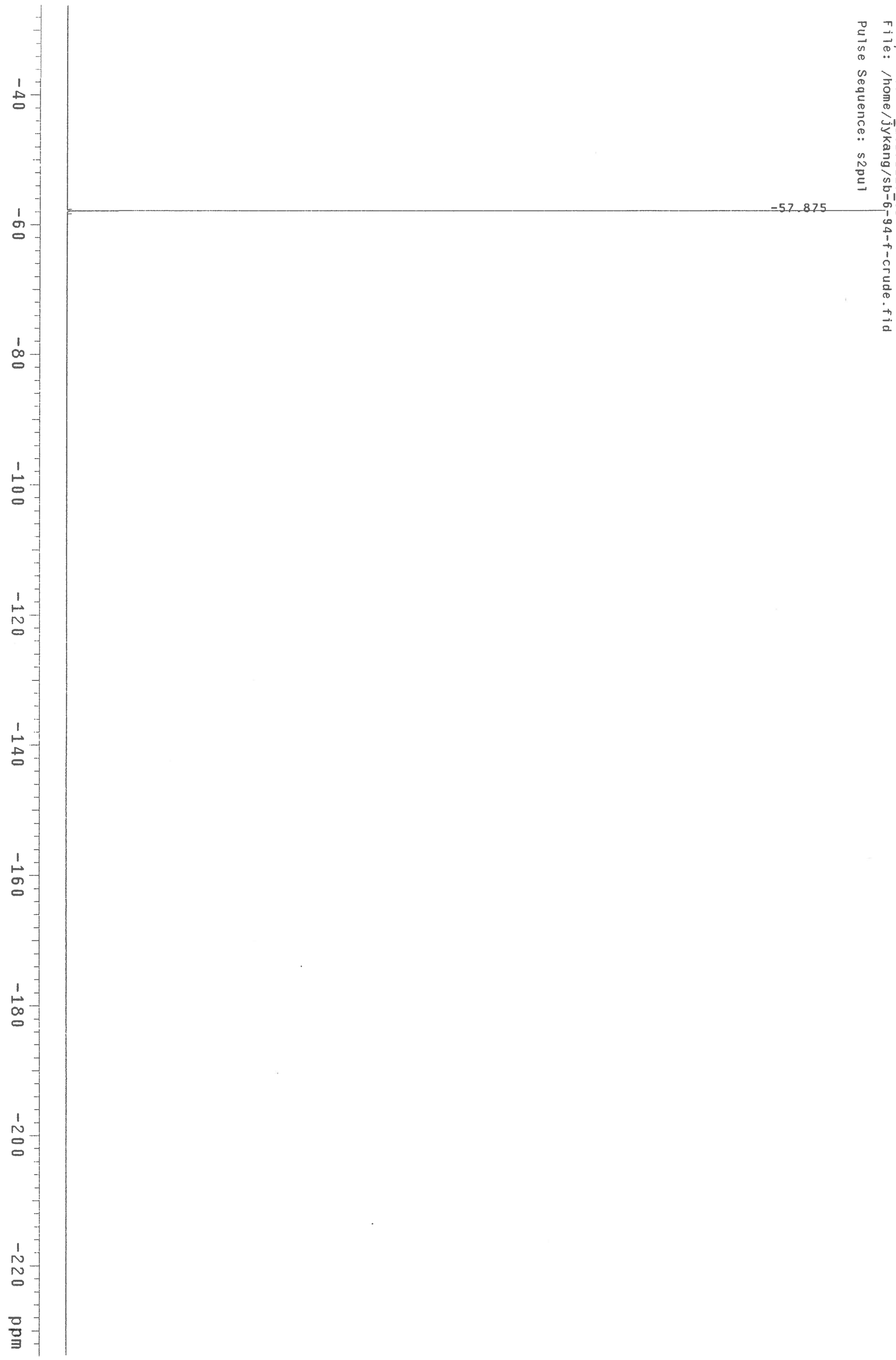

^{1}H NMR (400 MHz) in $CDCl_3$

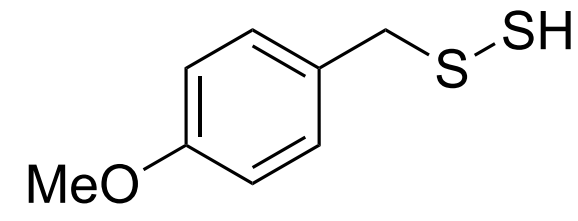

4f

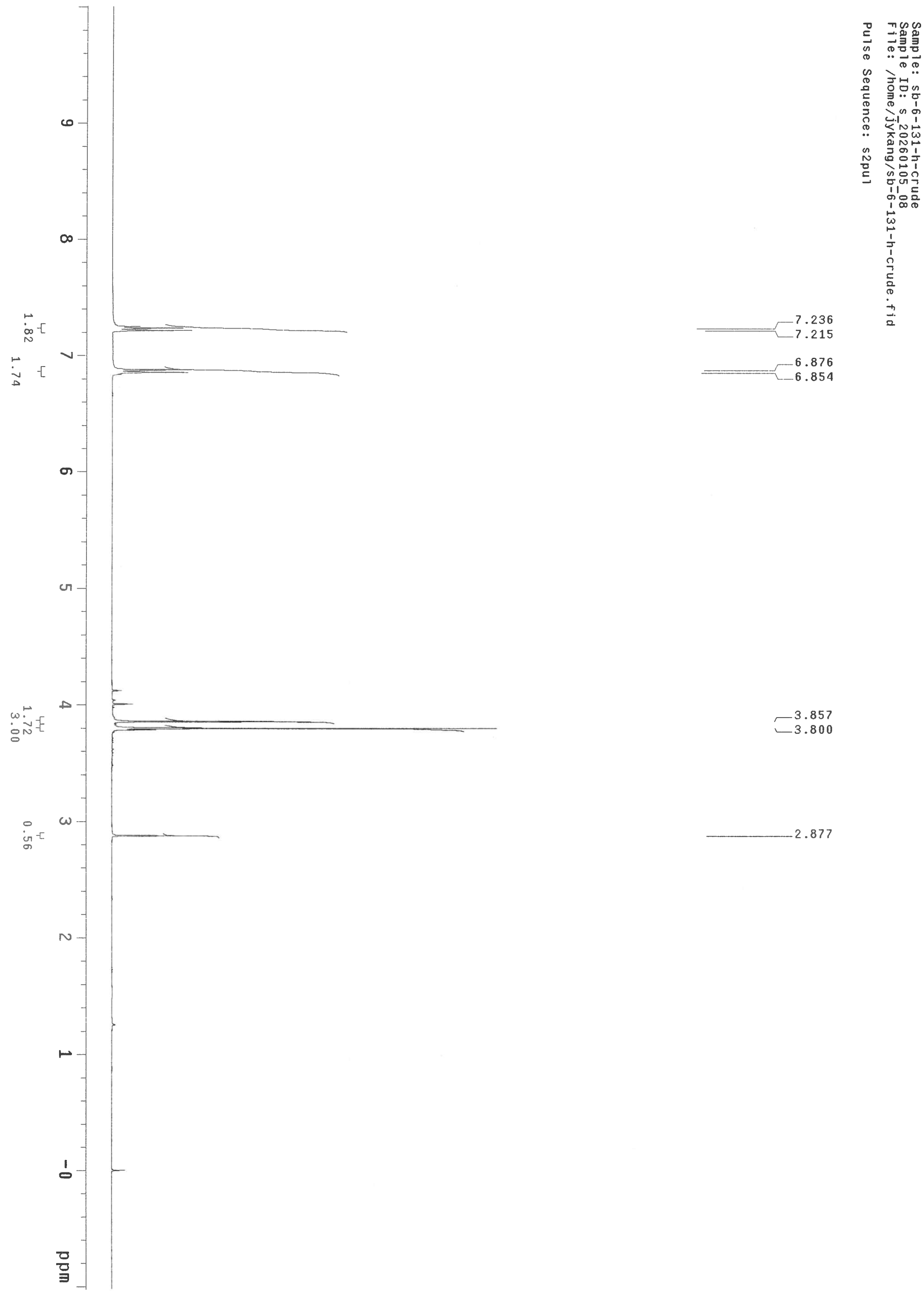

^{13}C NMR (100.5 MHz) in $CDCl_3$

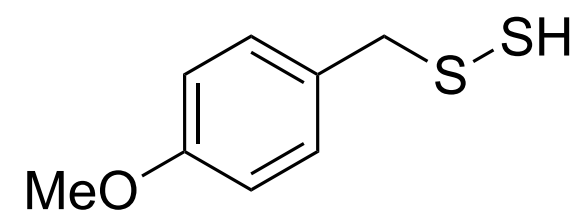

4f

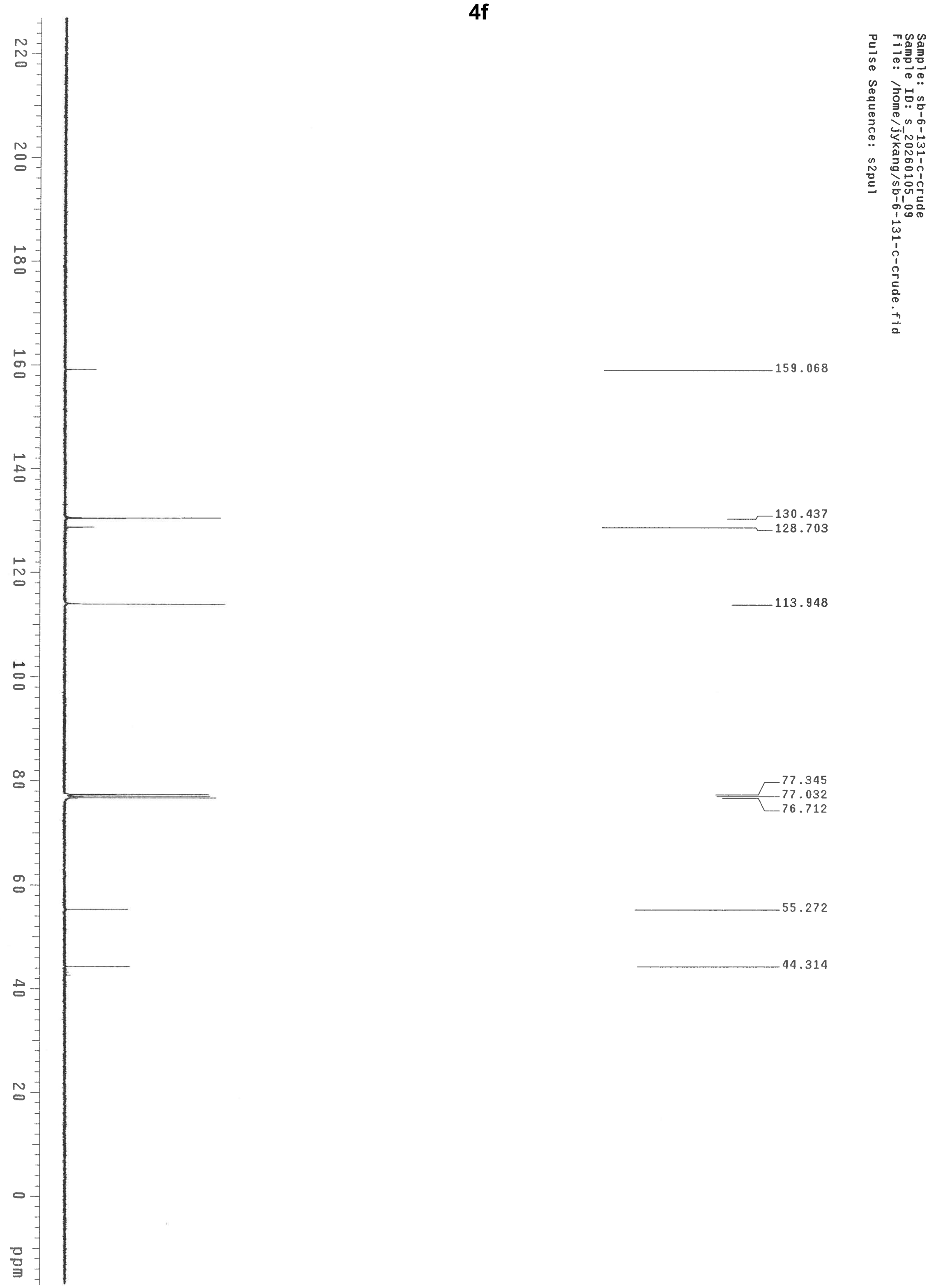

^{1}H NMR (400 MHz) in $CDCl_3$

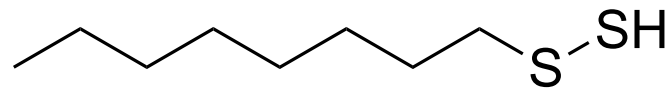

4h

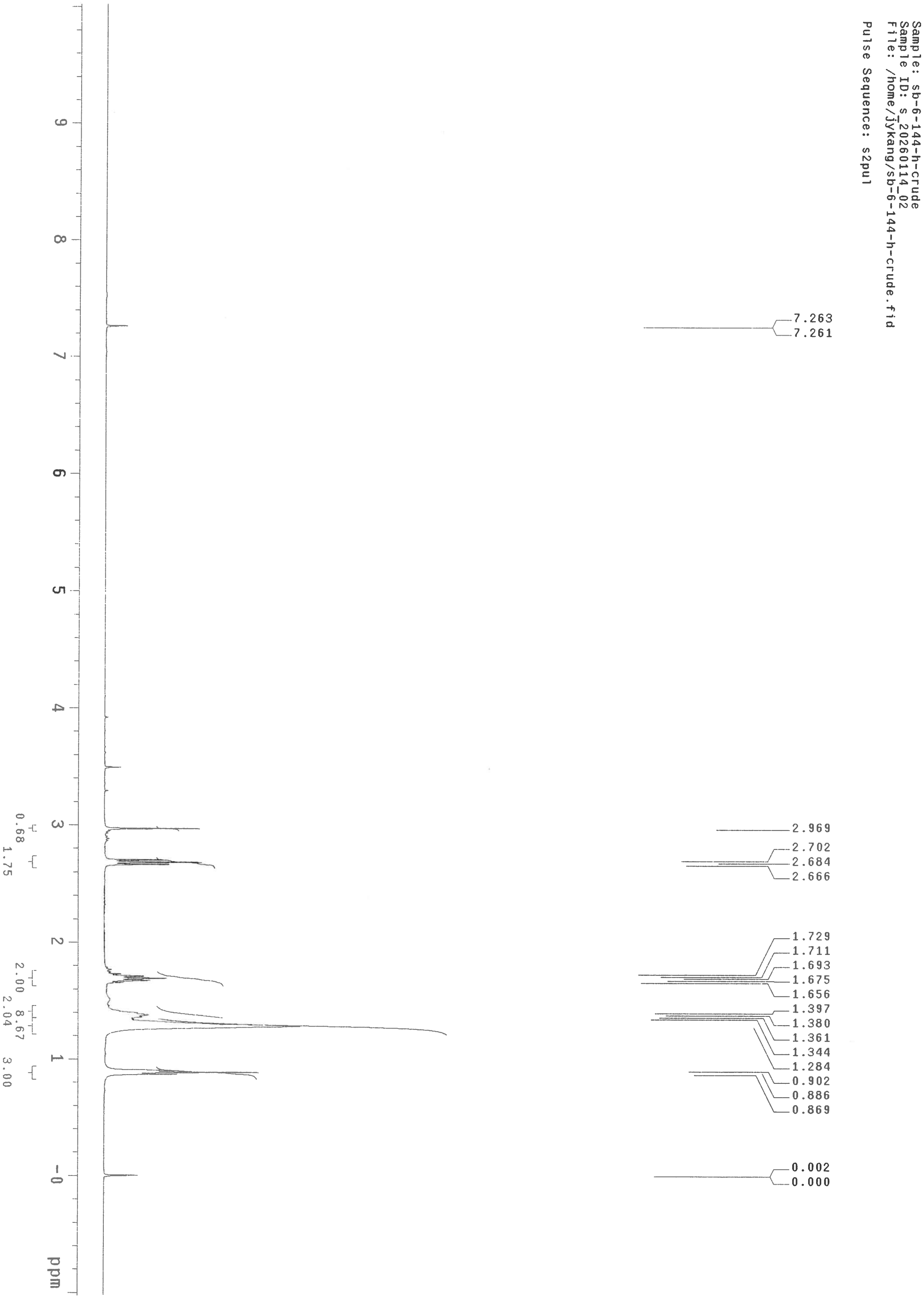

^{13}C NMR (100.5 MHz) in $CDCl_3$

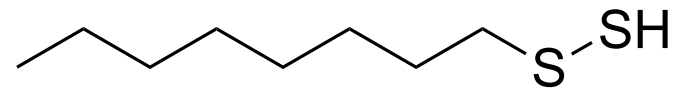

4h

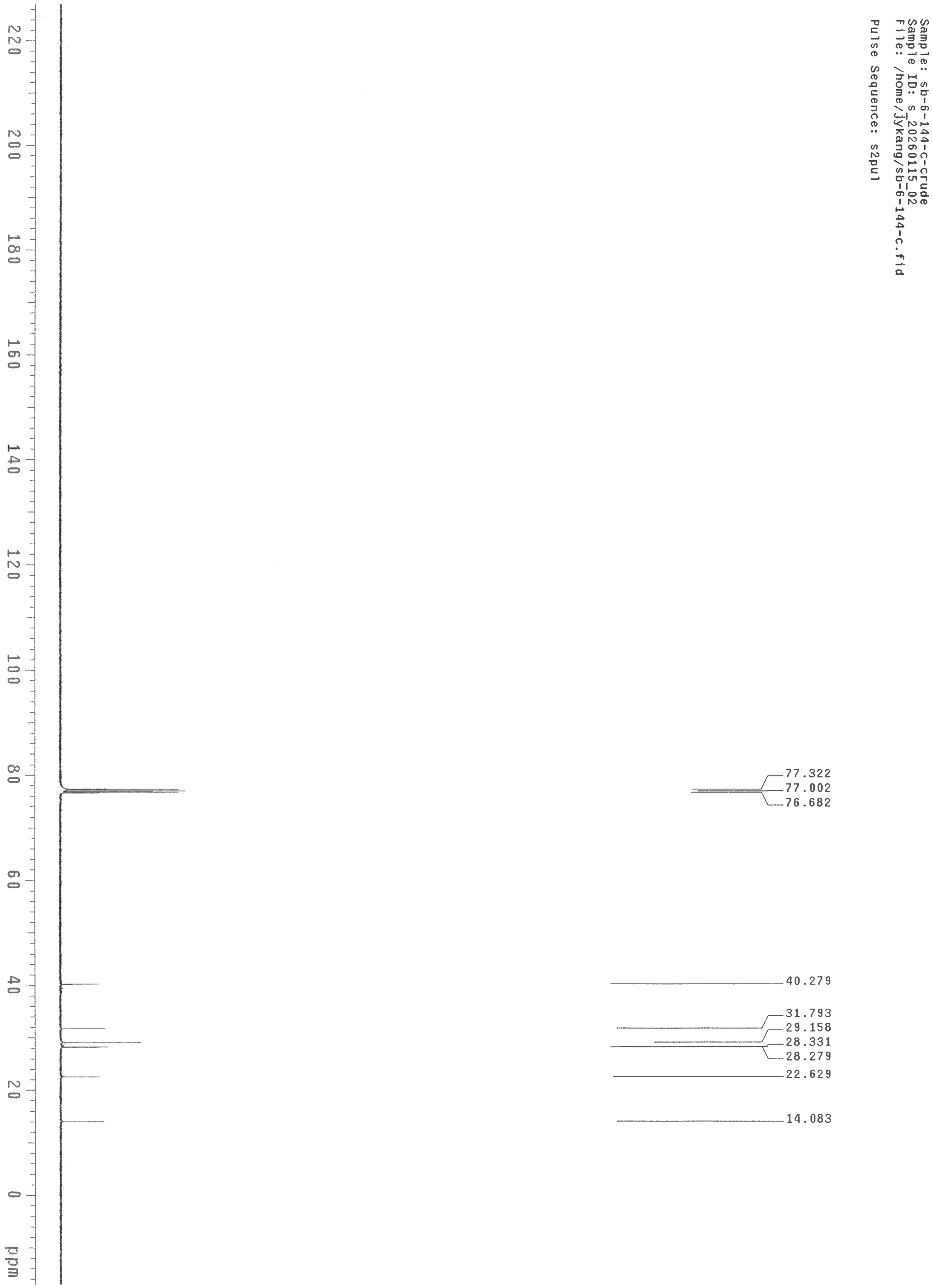

^{1}H NMR (400 MHz) in $CDCl_3$

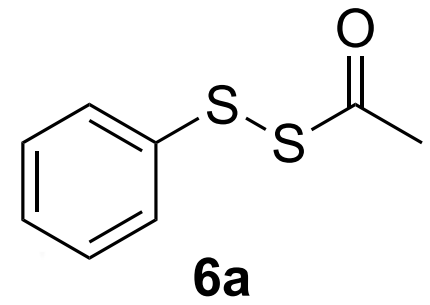

6a

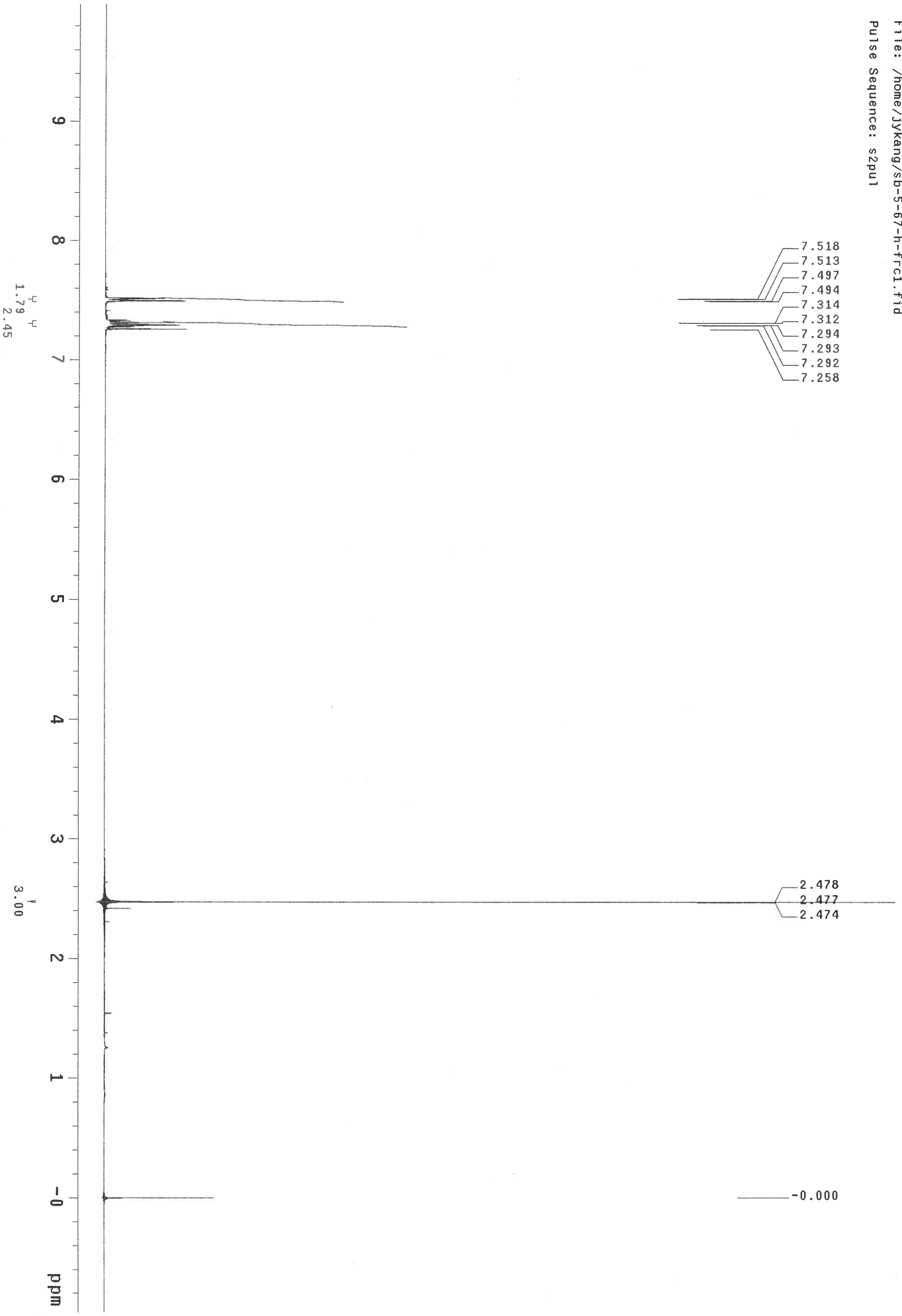

^{13}C NMR (100.5 MHz) in $CDCl_3$

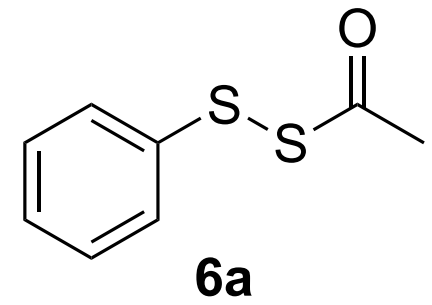

6a

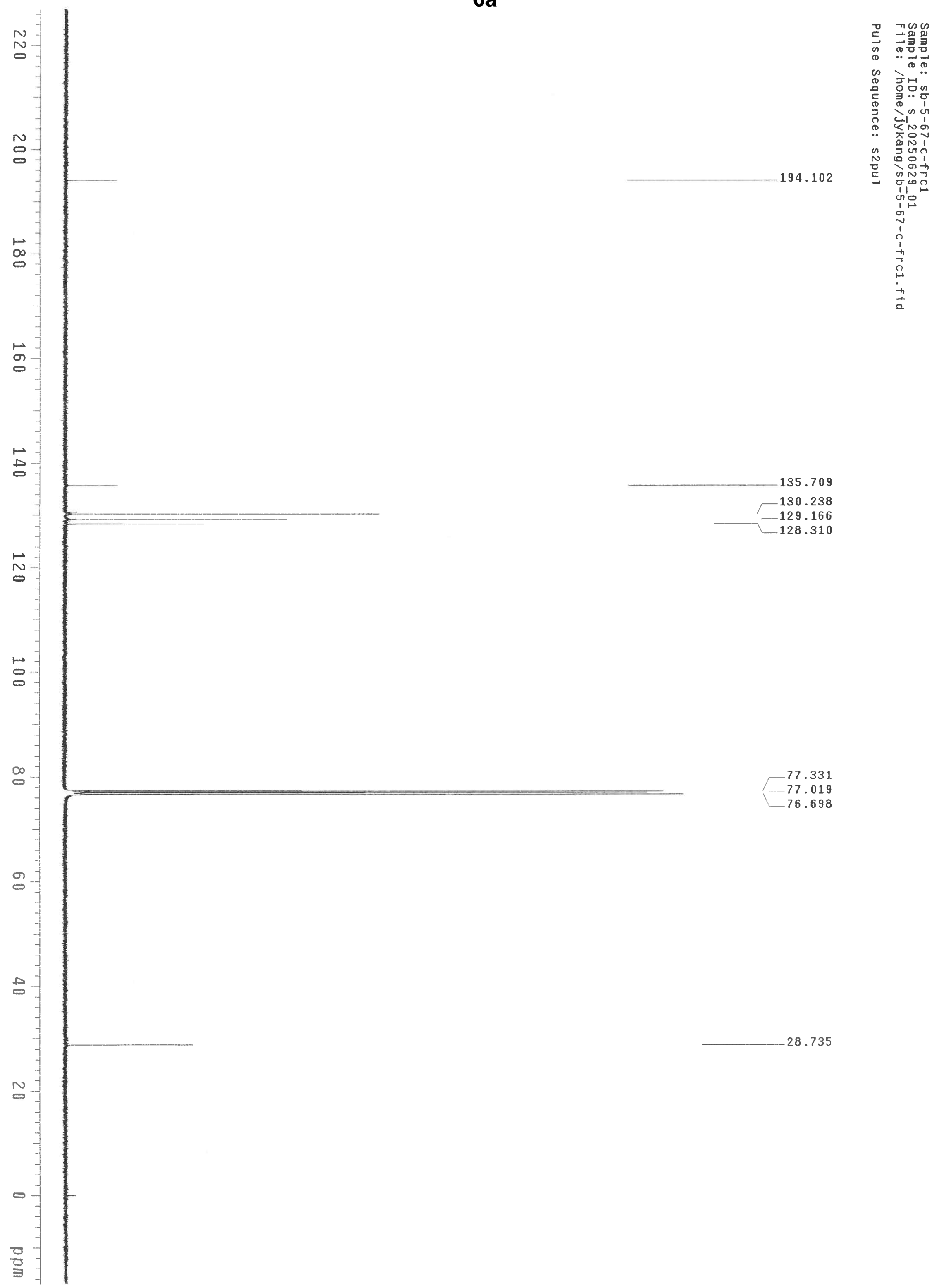

^{1}H NMR (400 MHz) in $CDCl_3$

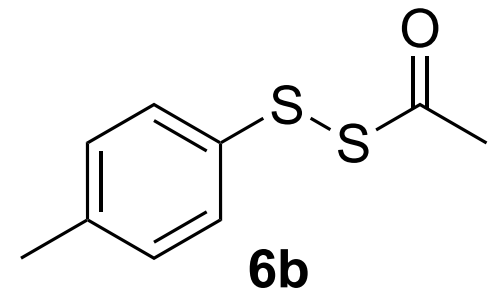

6b

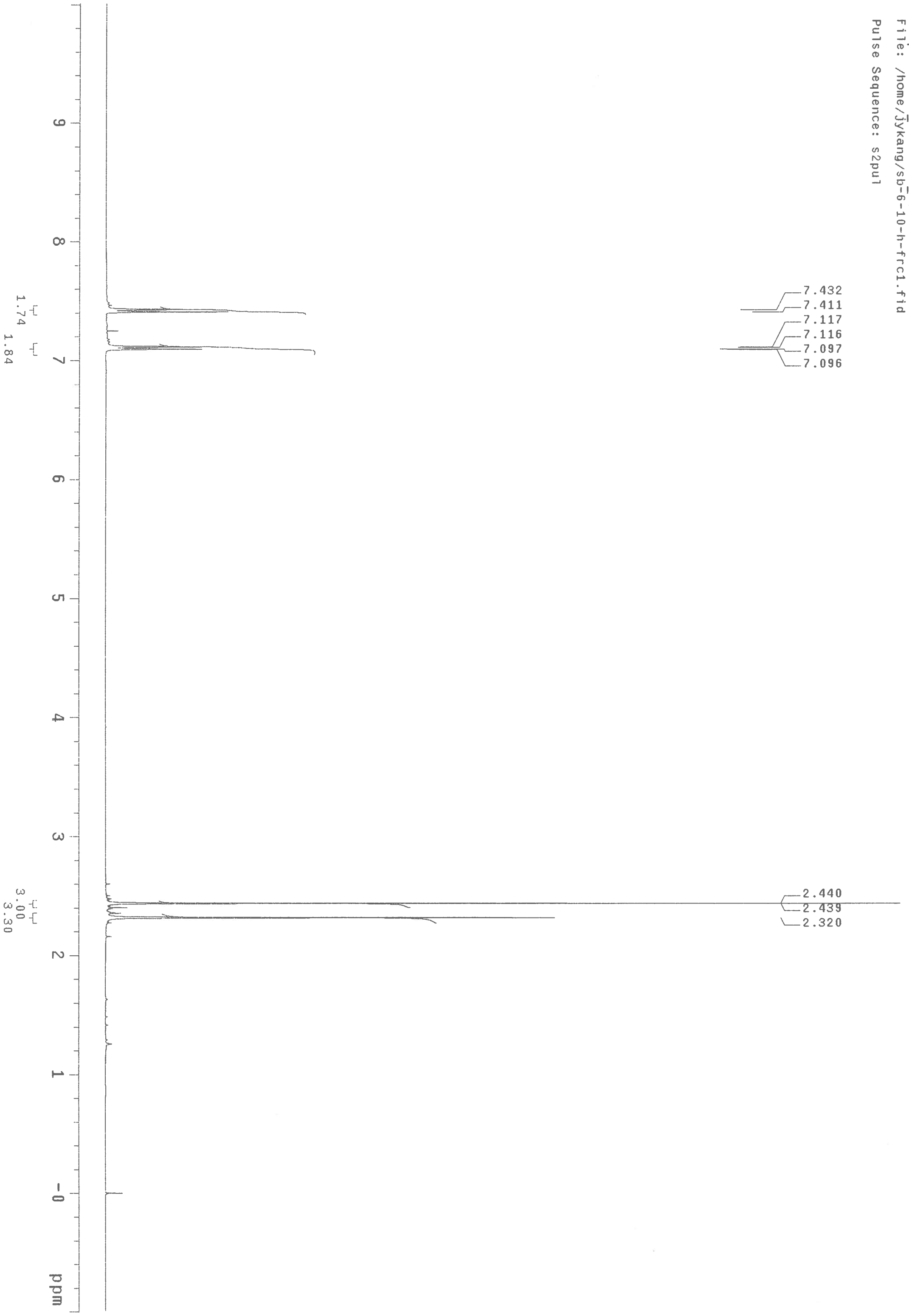

^{13}C NMR (100.5 MHz) in $CDCl_3$

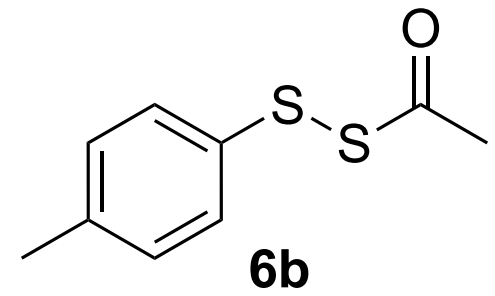

6b

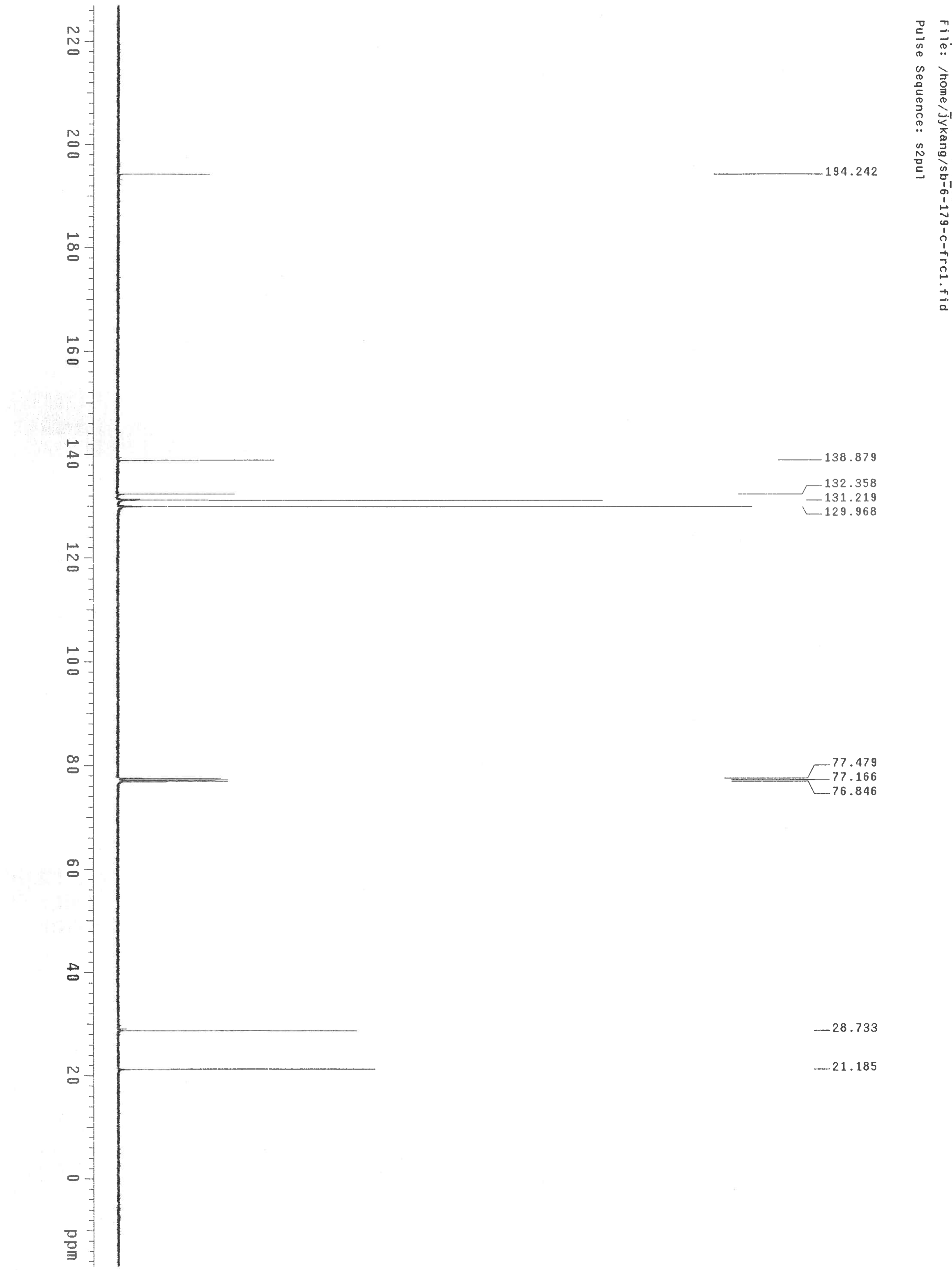

^{1}H NMR (400 MHz) in $CDCl_3$

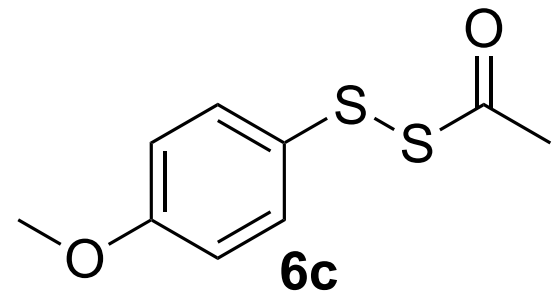

6c

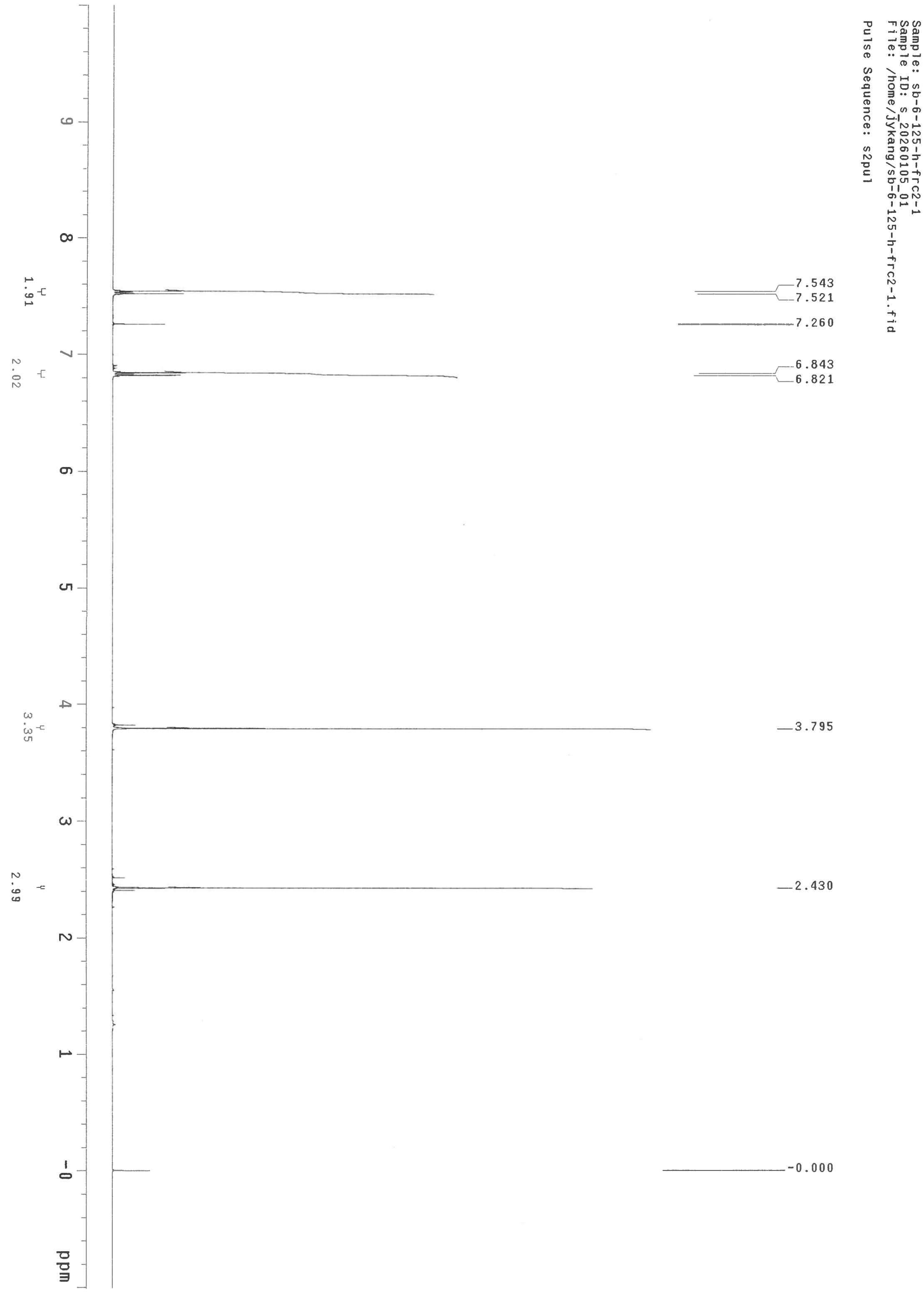

^{13}C NMR (100.5 MHz) in $CDCl_3$

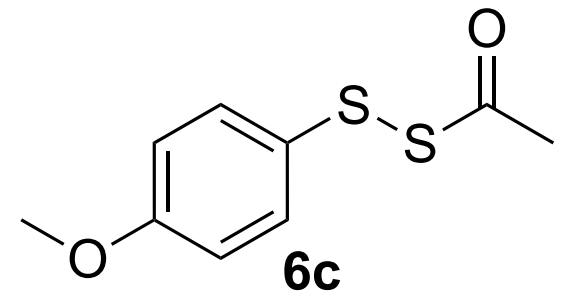

6c

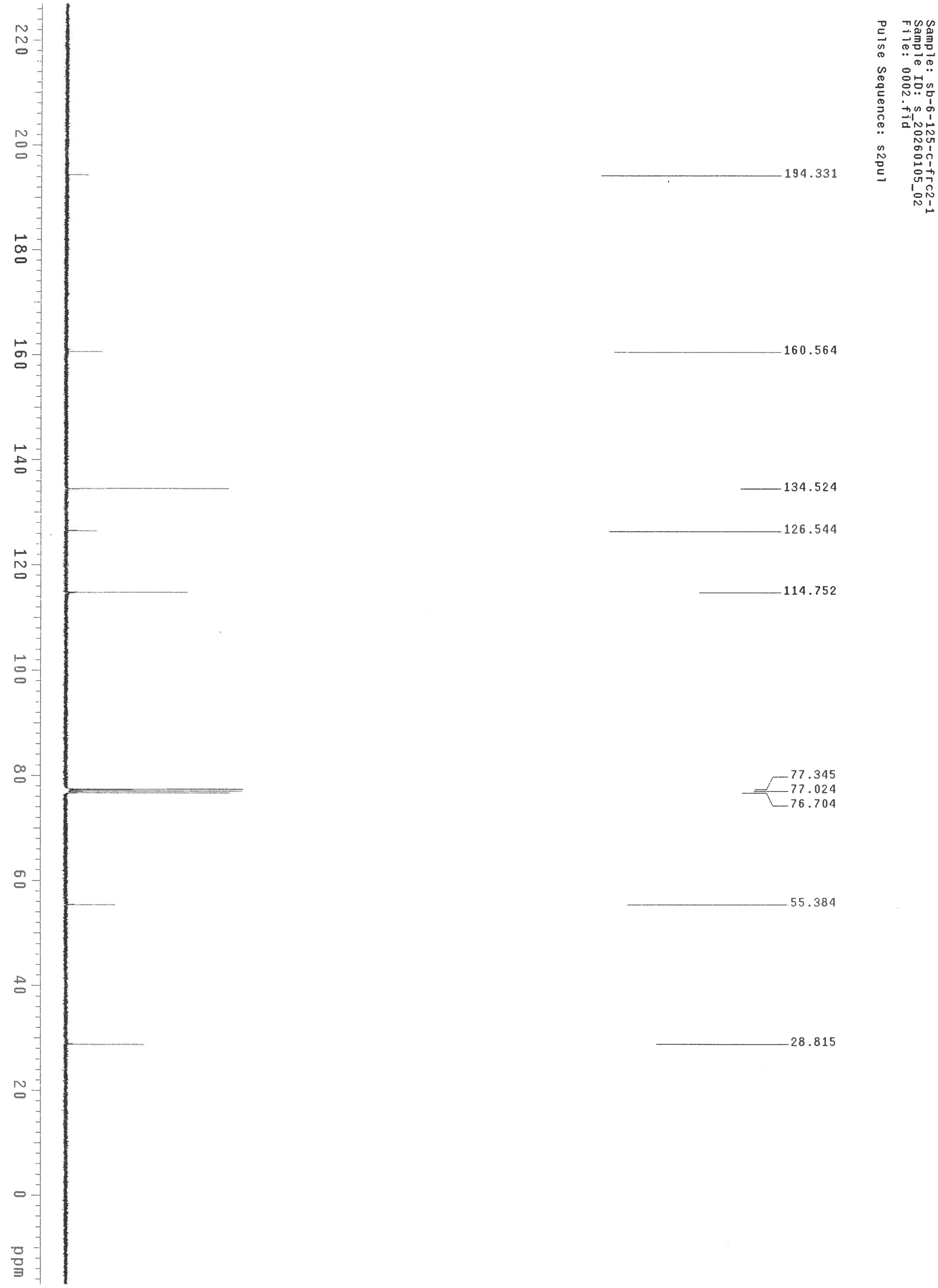

^{1}H NMR (400 MHz) in $CDCl_3$

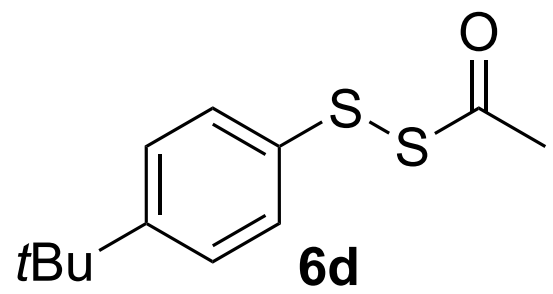

6d

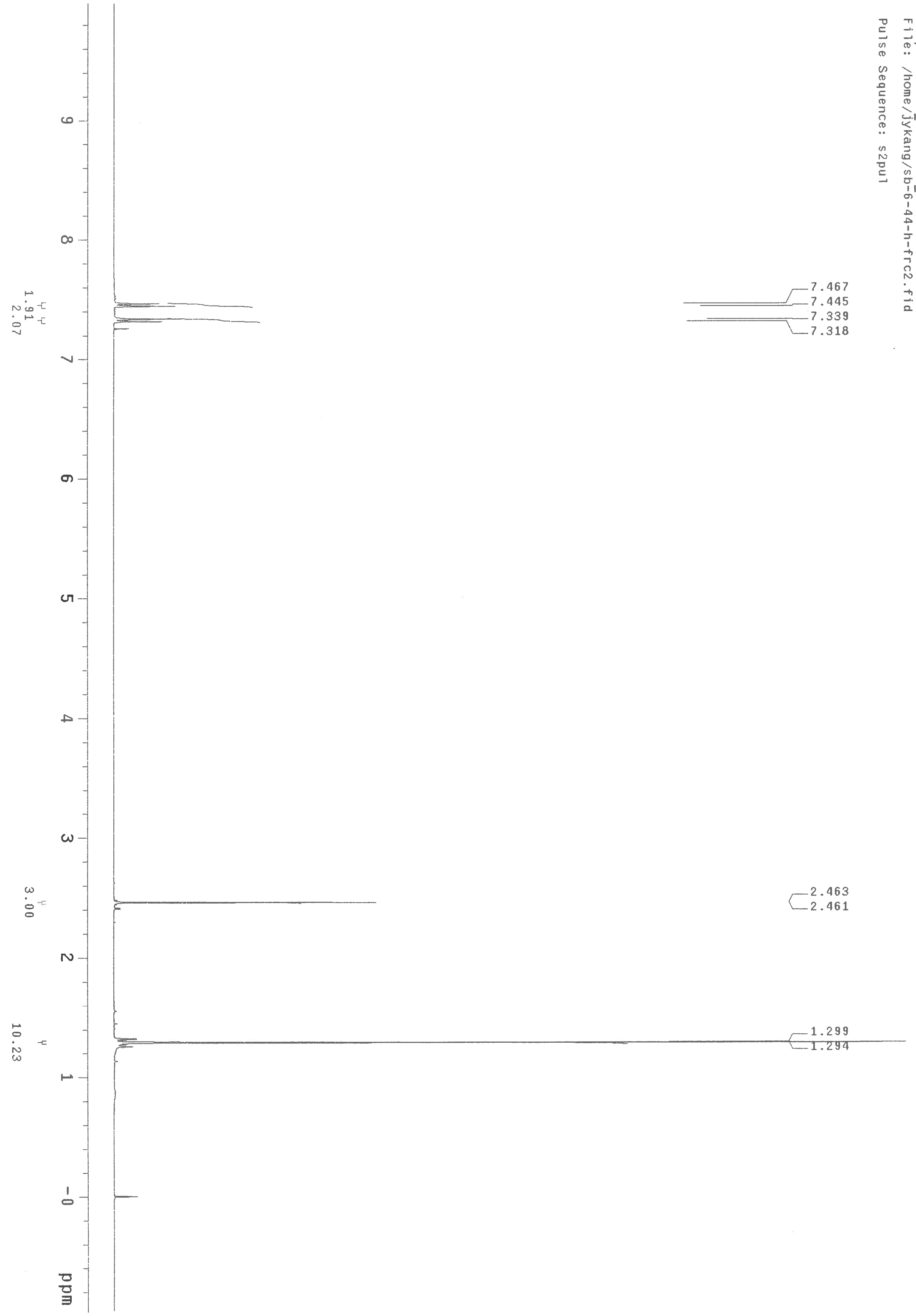

^{13}C NMR (100.5 MHz) in $CDCl_3$

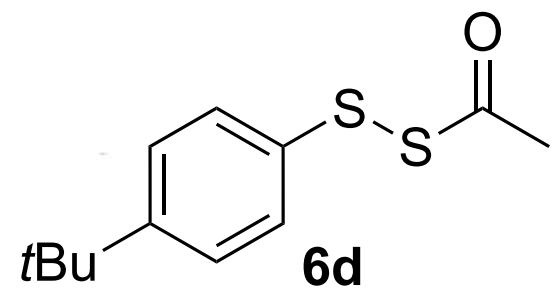

6d

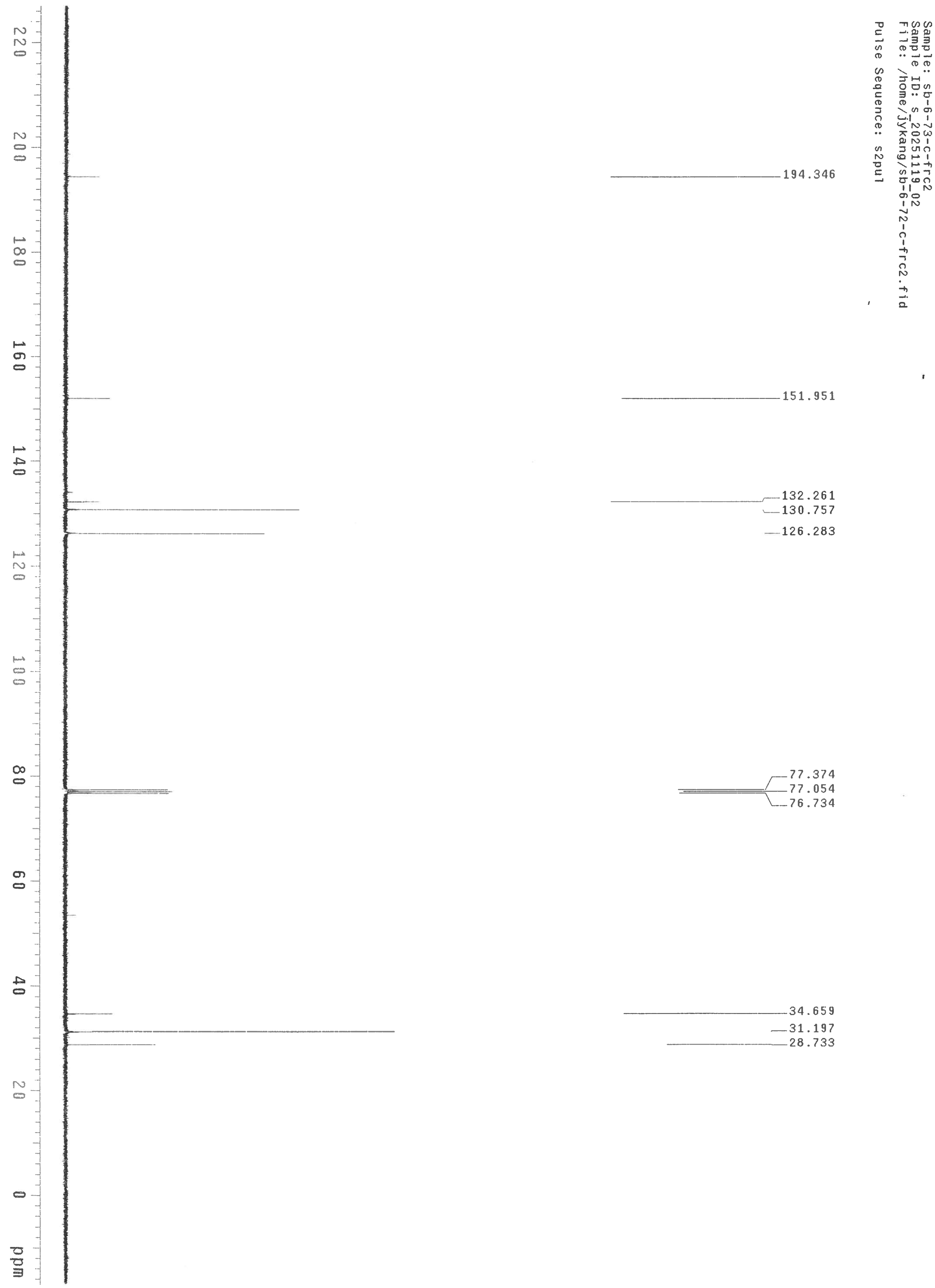

^{1}H NMR (400 MHz) in $CDCl_3$

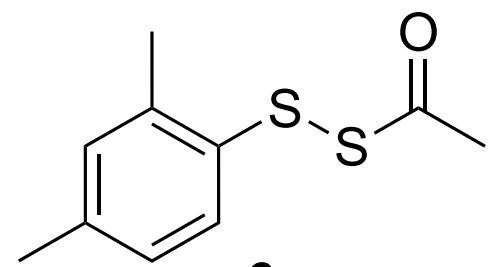

6e

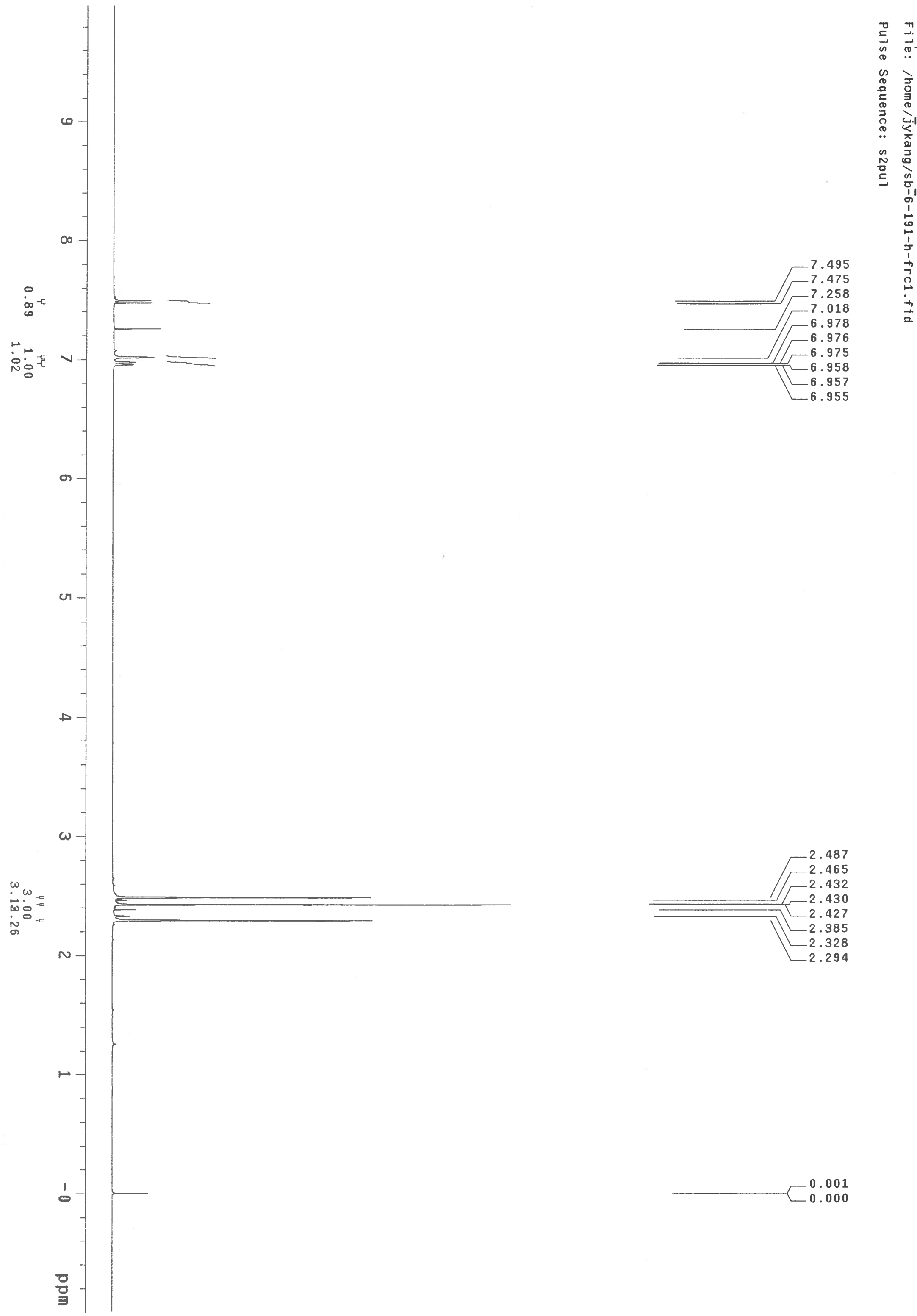

^{13}C NMR (100.5 MHz) in $CDCl_3$

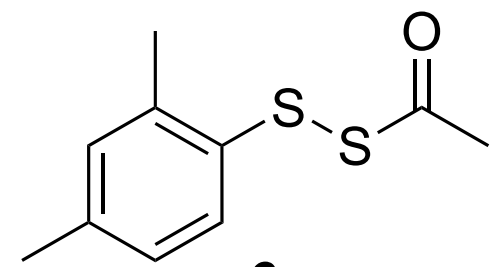

6e

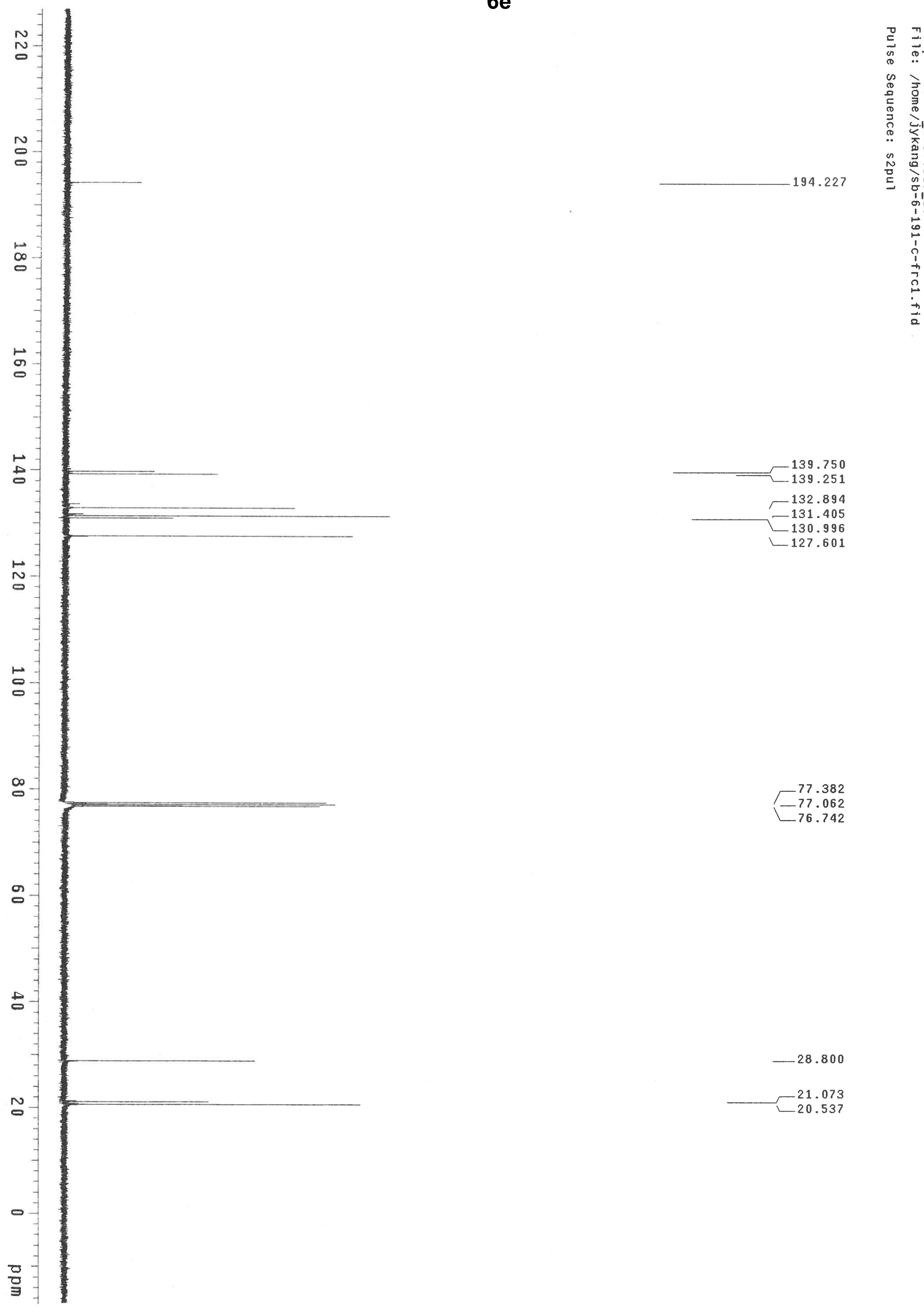

^{1}H NMR (400 MHz) in $CDCl_3$

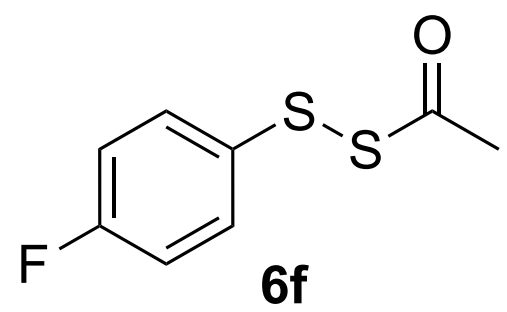

6f

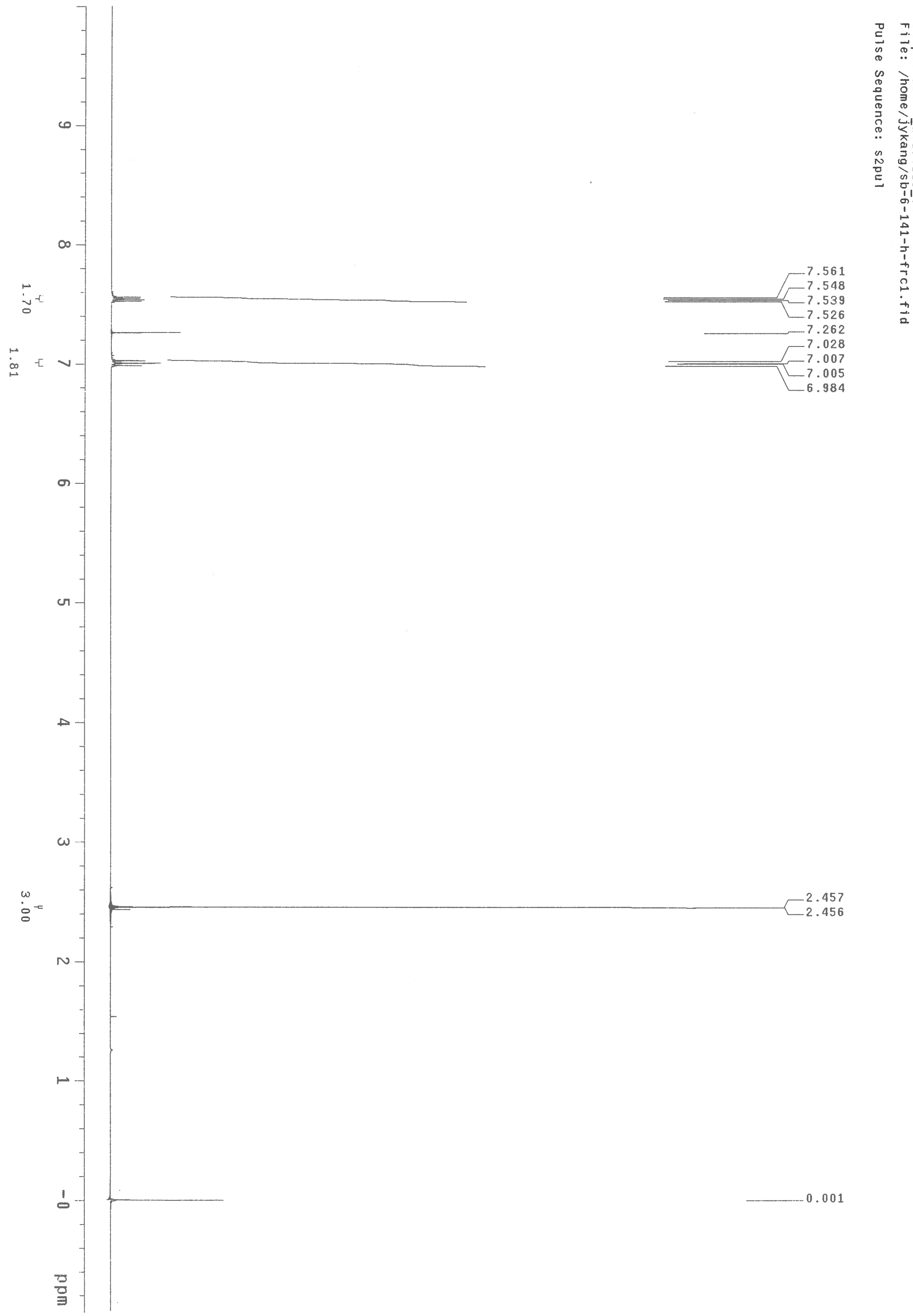

^{13}C NMR (100.5 MHz) in $CDCl_3$

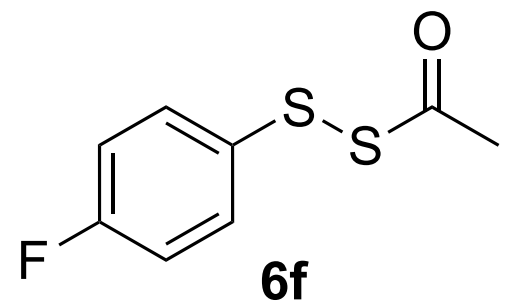

6f

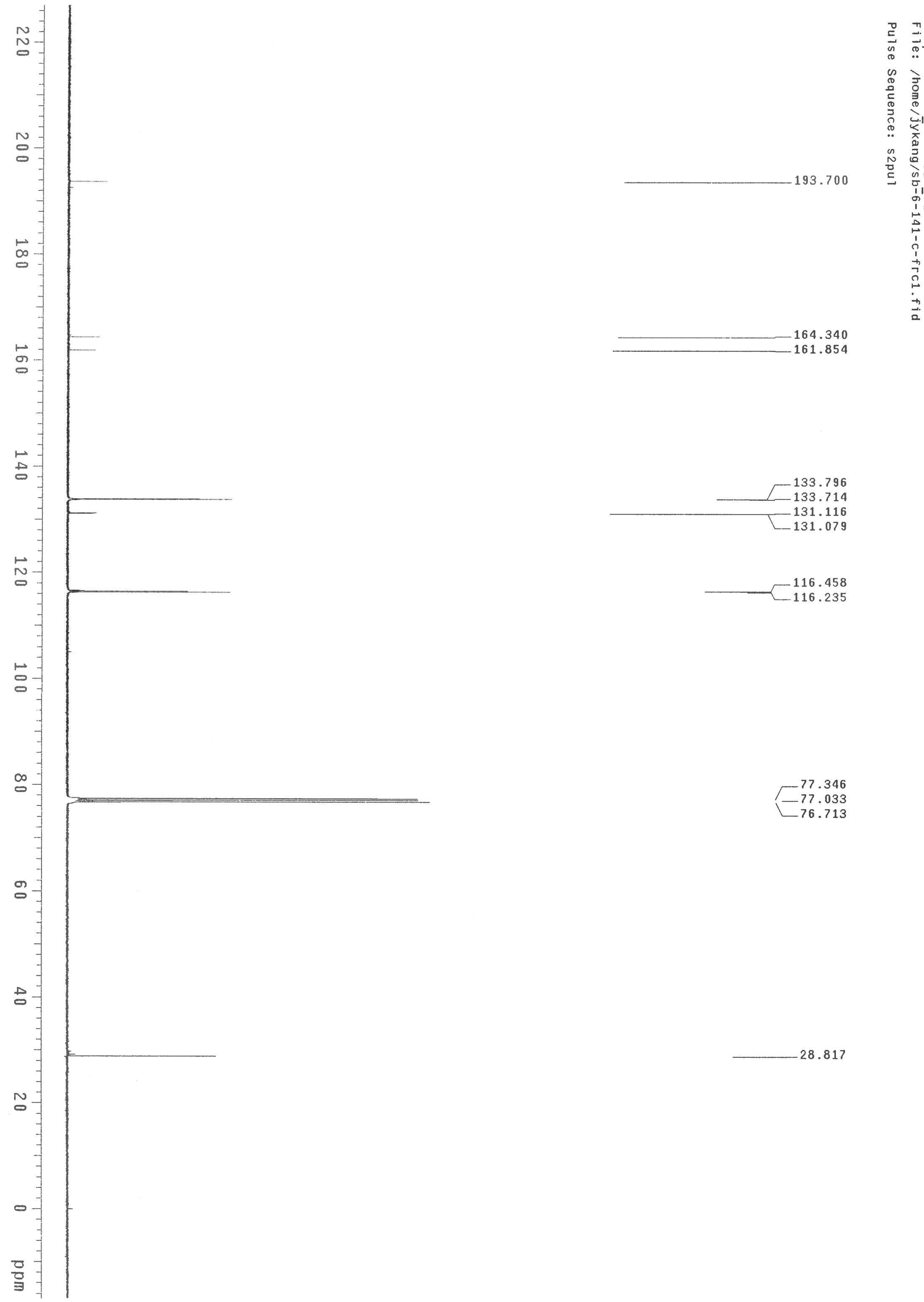

^{19}F NMR (375.9 MHz) in $CDCl_3$

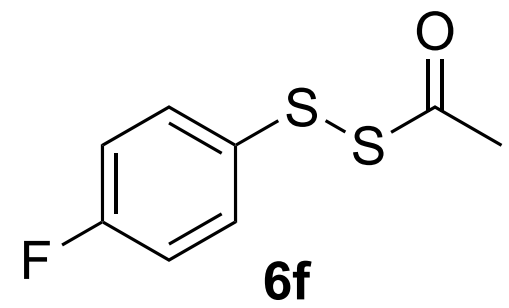

6f

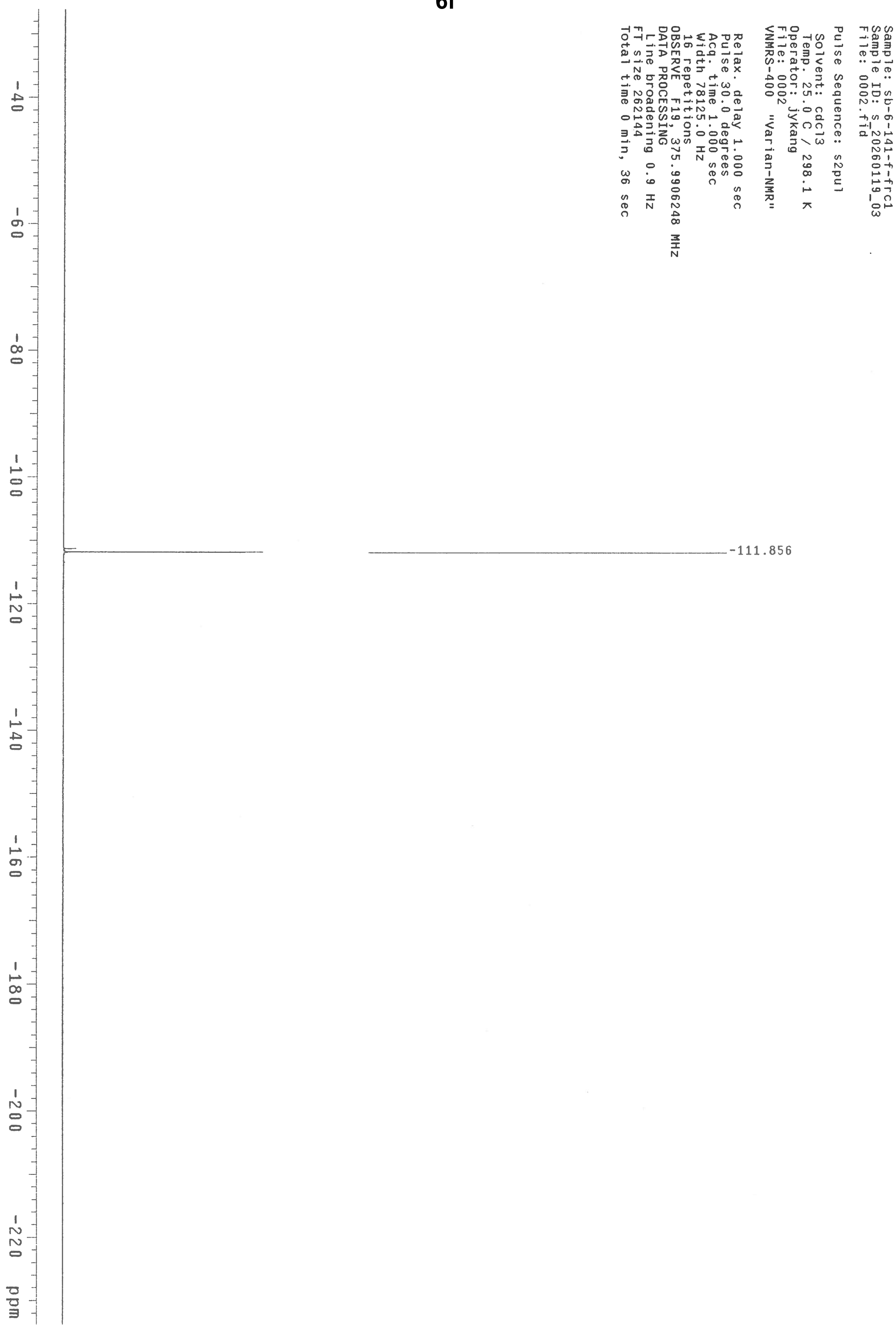

^{1}H NMR (400 MHz) in $CDCl_3$

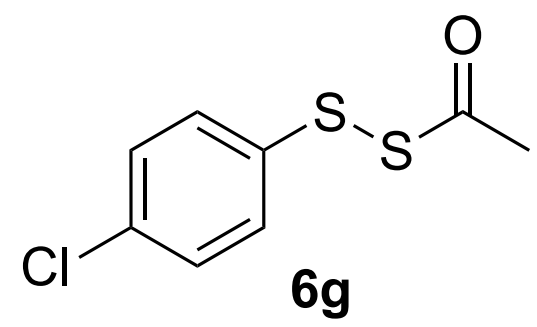

6g

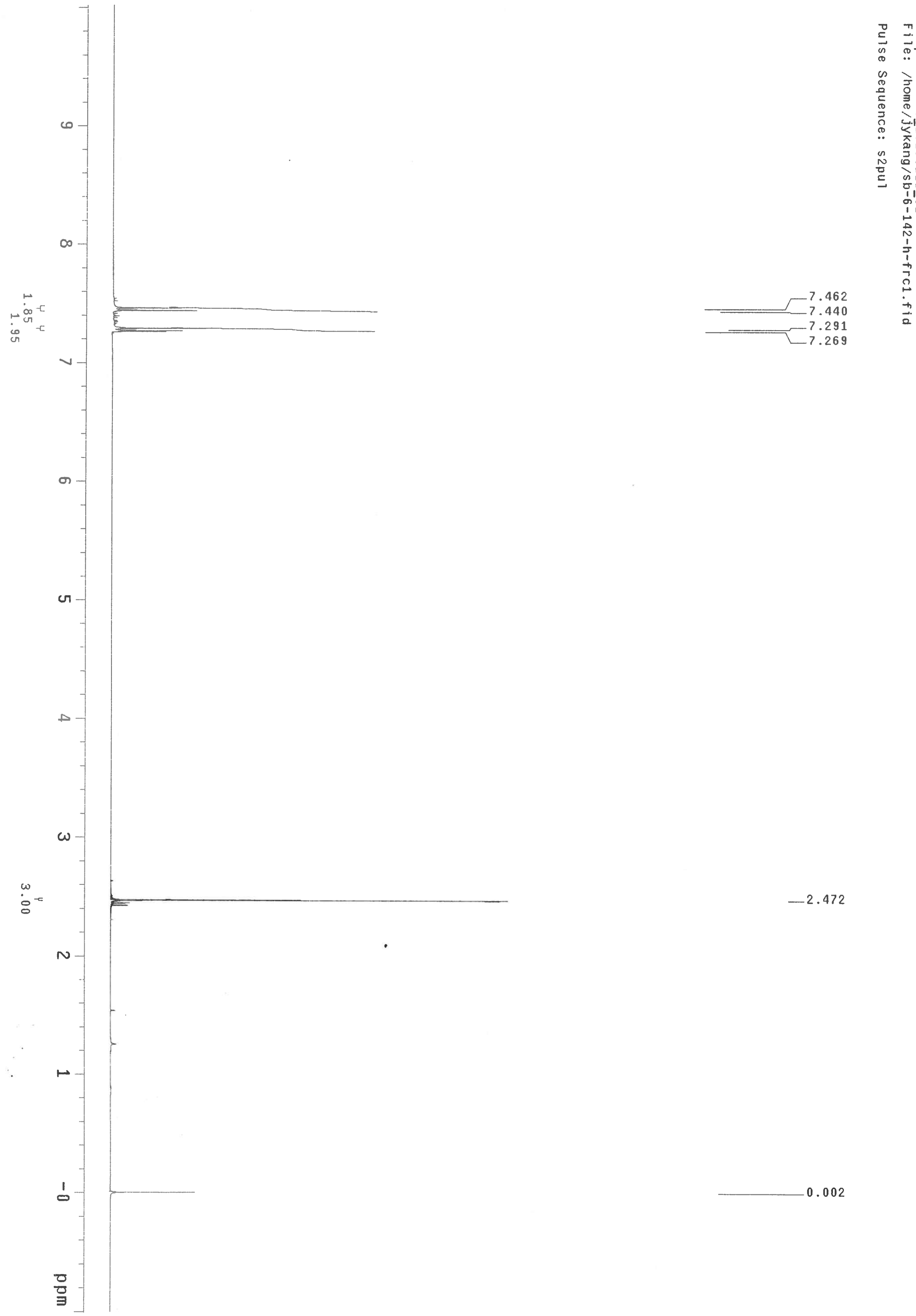

^{13}C NMR (100.5 MHz) in $CDCl_3$

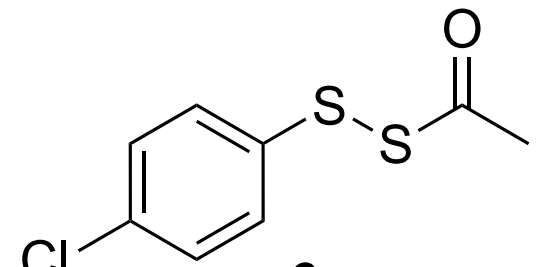

6g

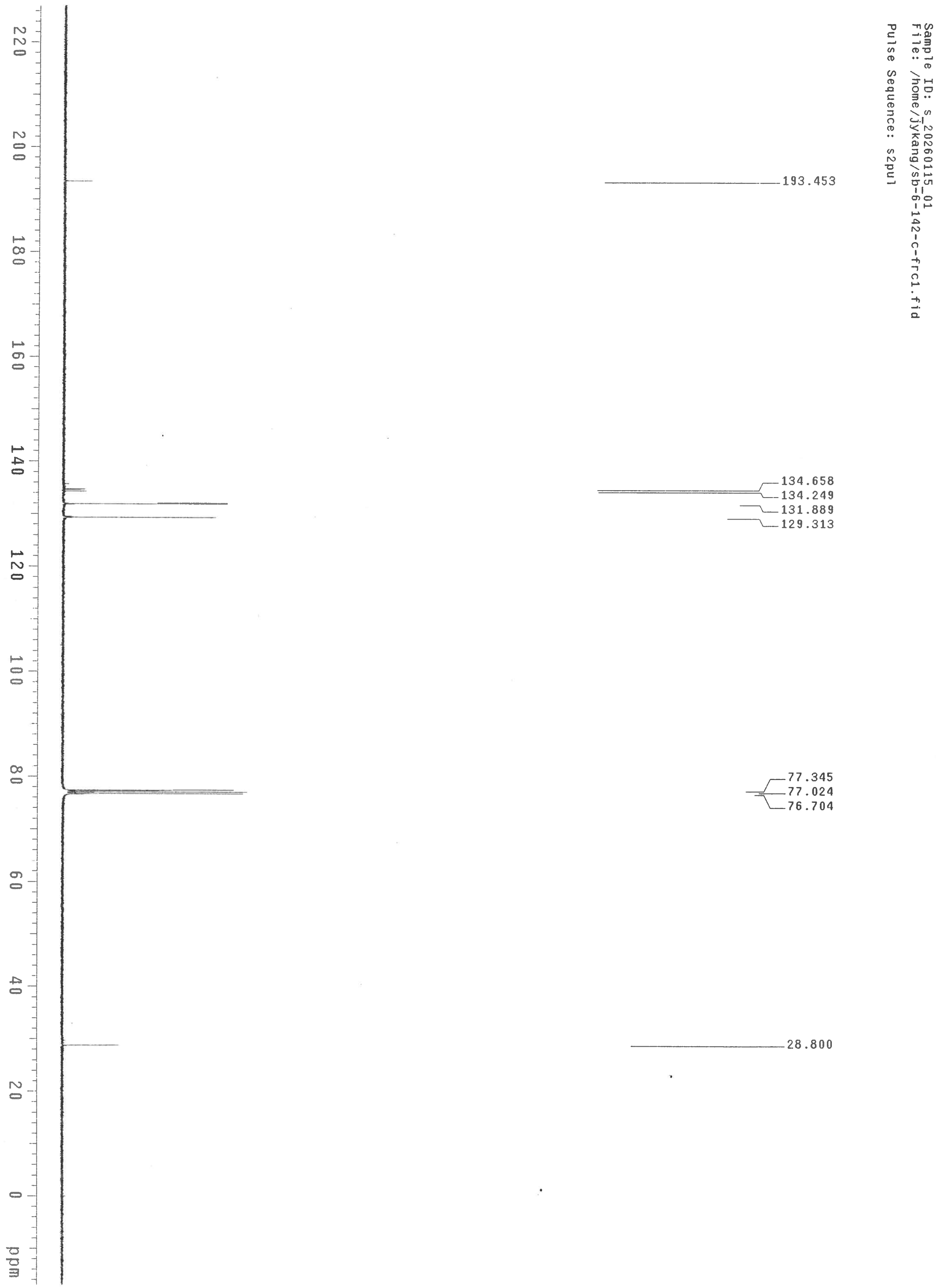

^{1}H NMR (400 MHz) in $CDCl_3$

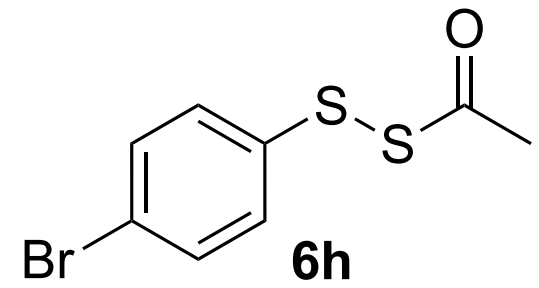

6h

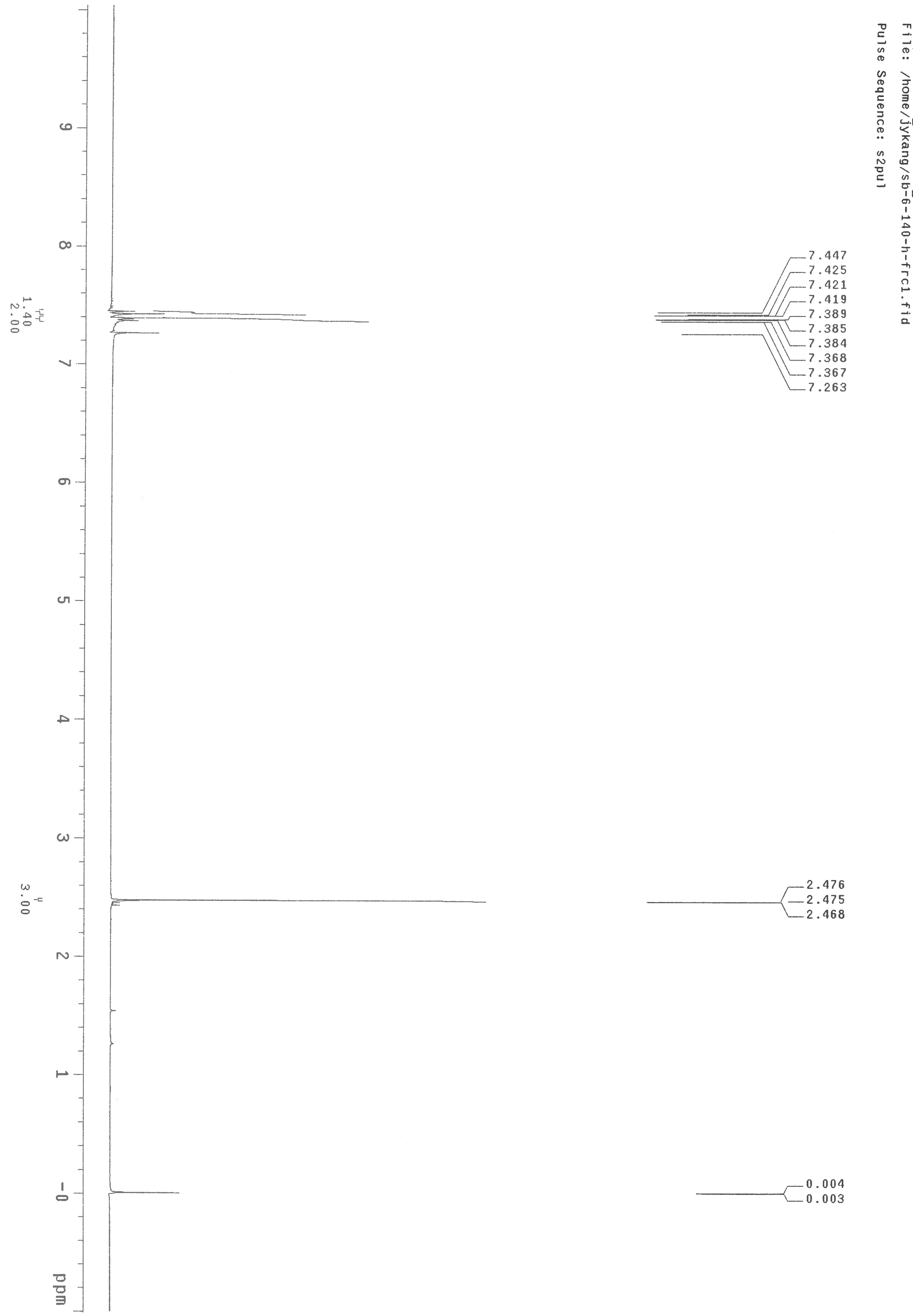

^{13}C NMR (100.5 MHz) in $CDCl_3$

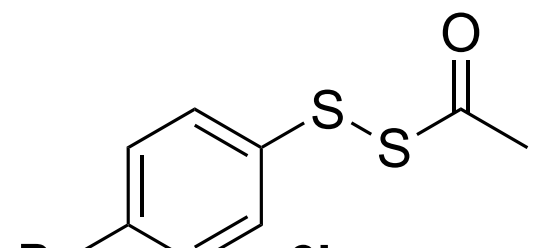

6h

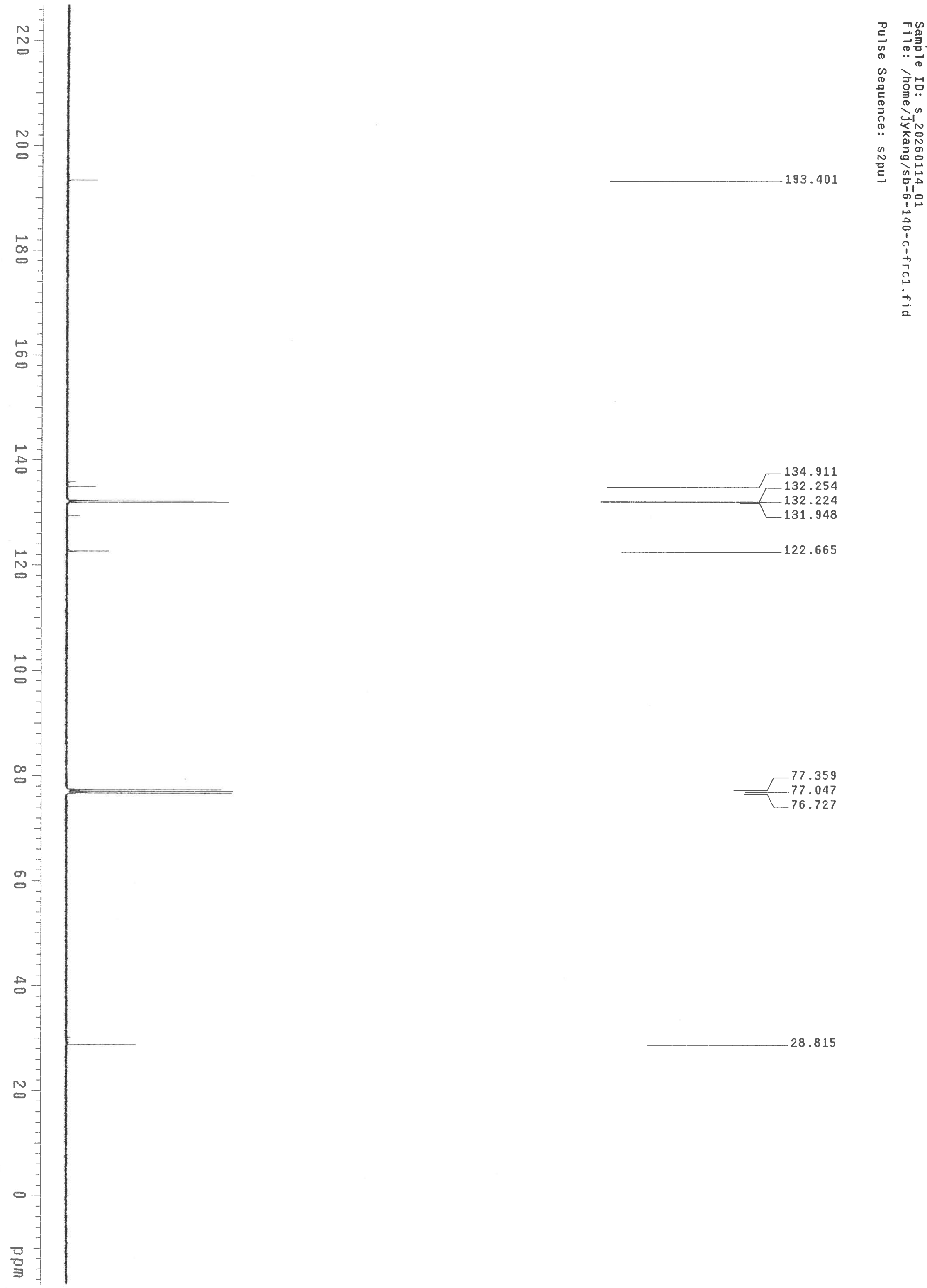

^{1}H NMR (400 MHz) in $CDCl_3$

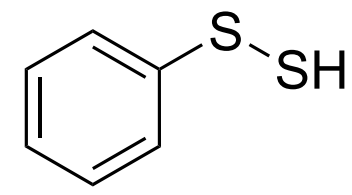

7a

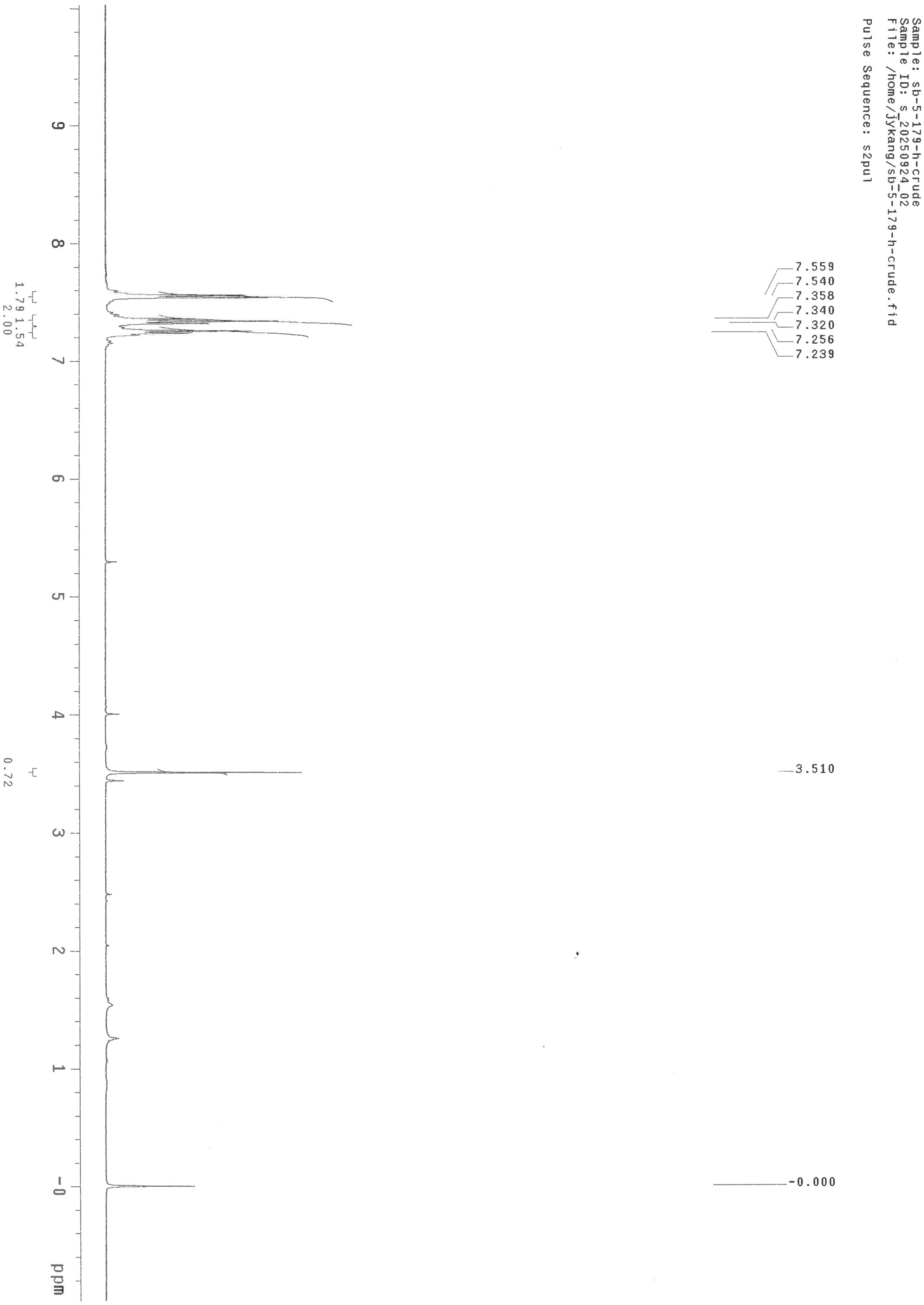

^{13}C NMR (100.5 MHz) in $CDCl_3$

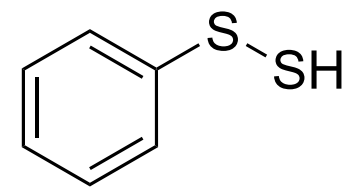

7a

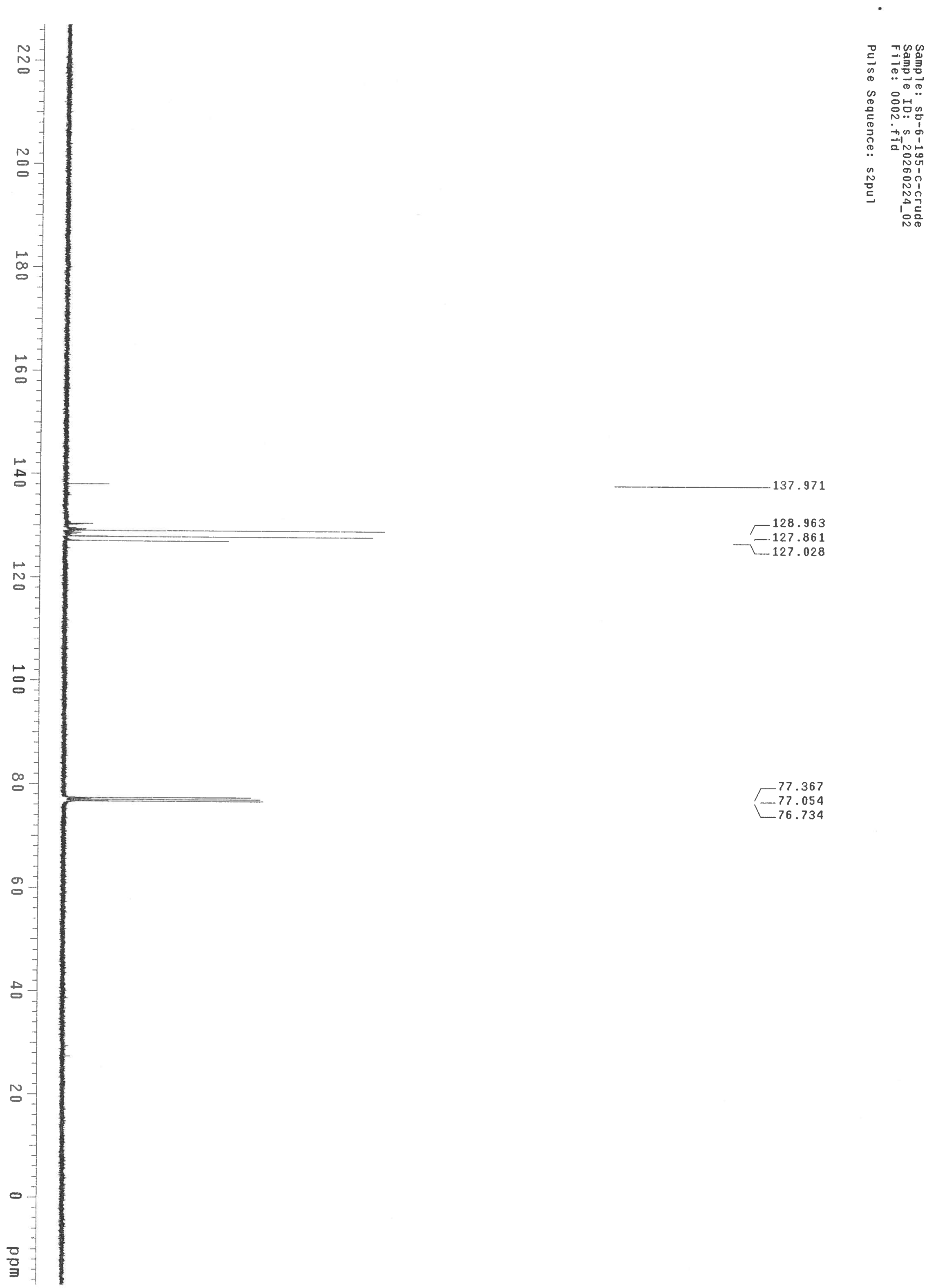

^{1}H NMR (400 MHz) in $CDCl_3$

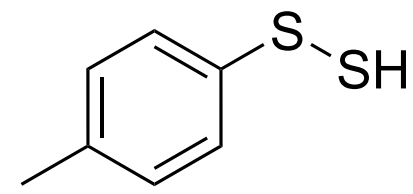

7b

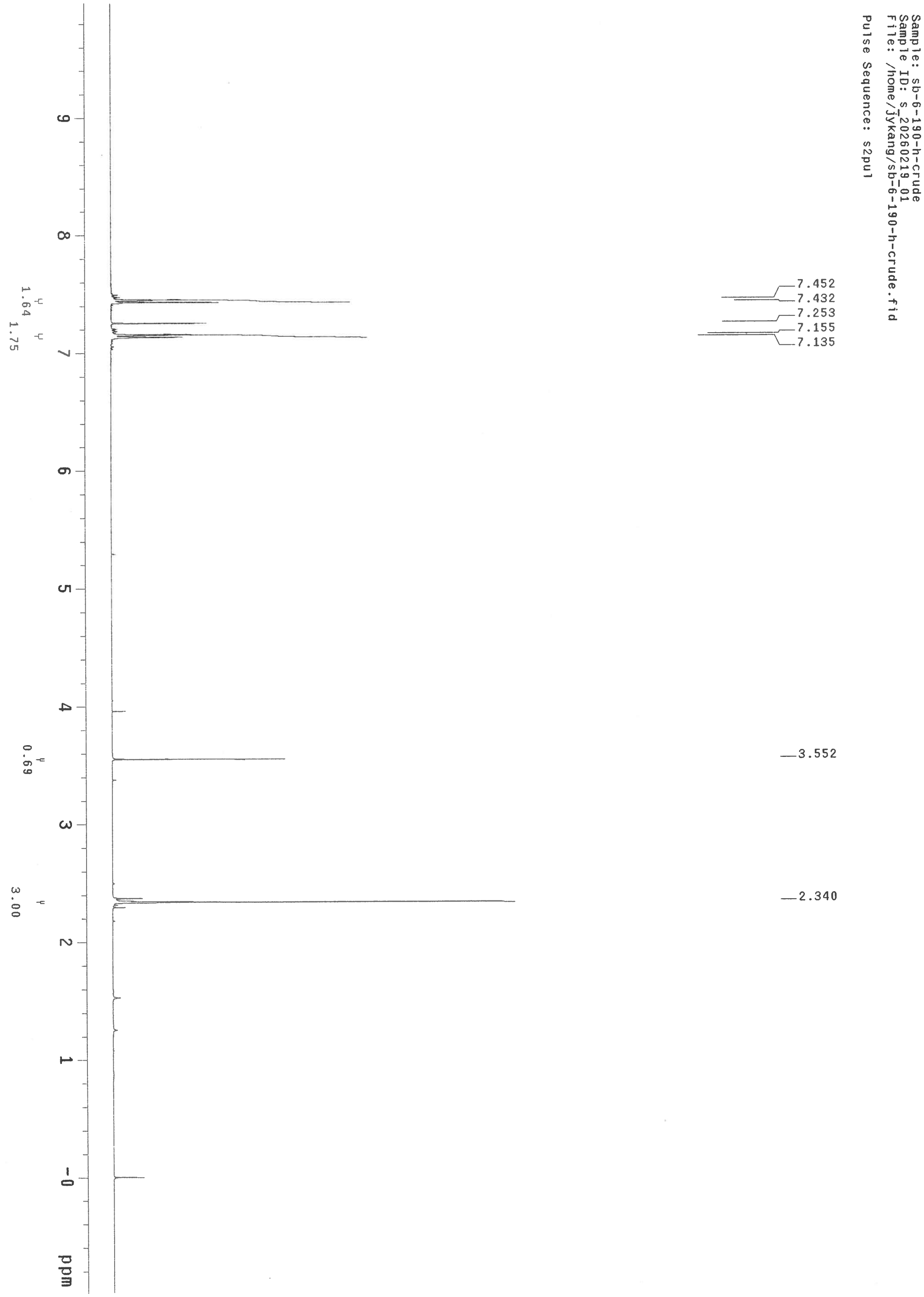

^{13}C NMR (100.5 MHz) in $CDCl_3$

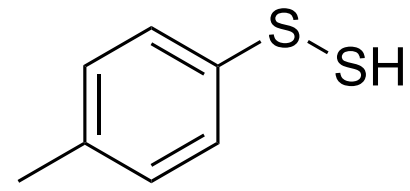

7b

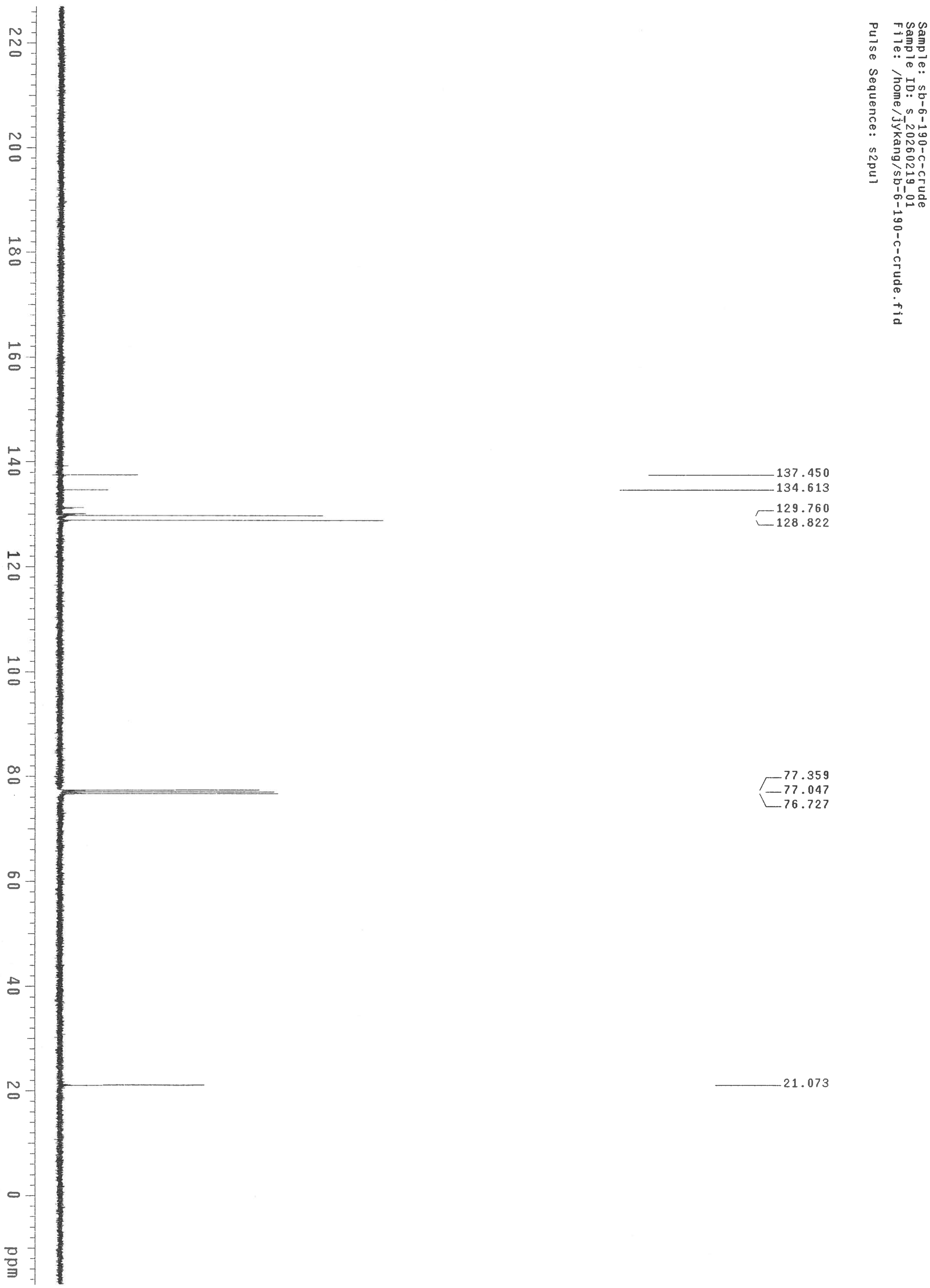

^{1}H NMR (400 MHz) in $CDCl_3$

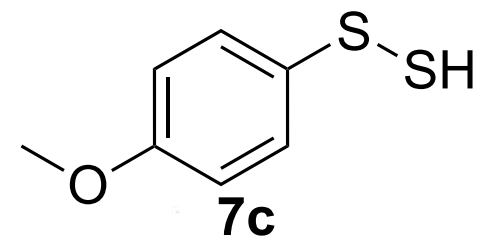

7c

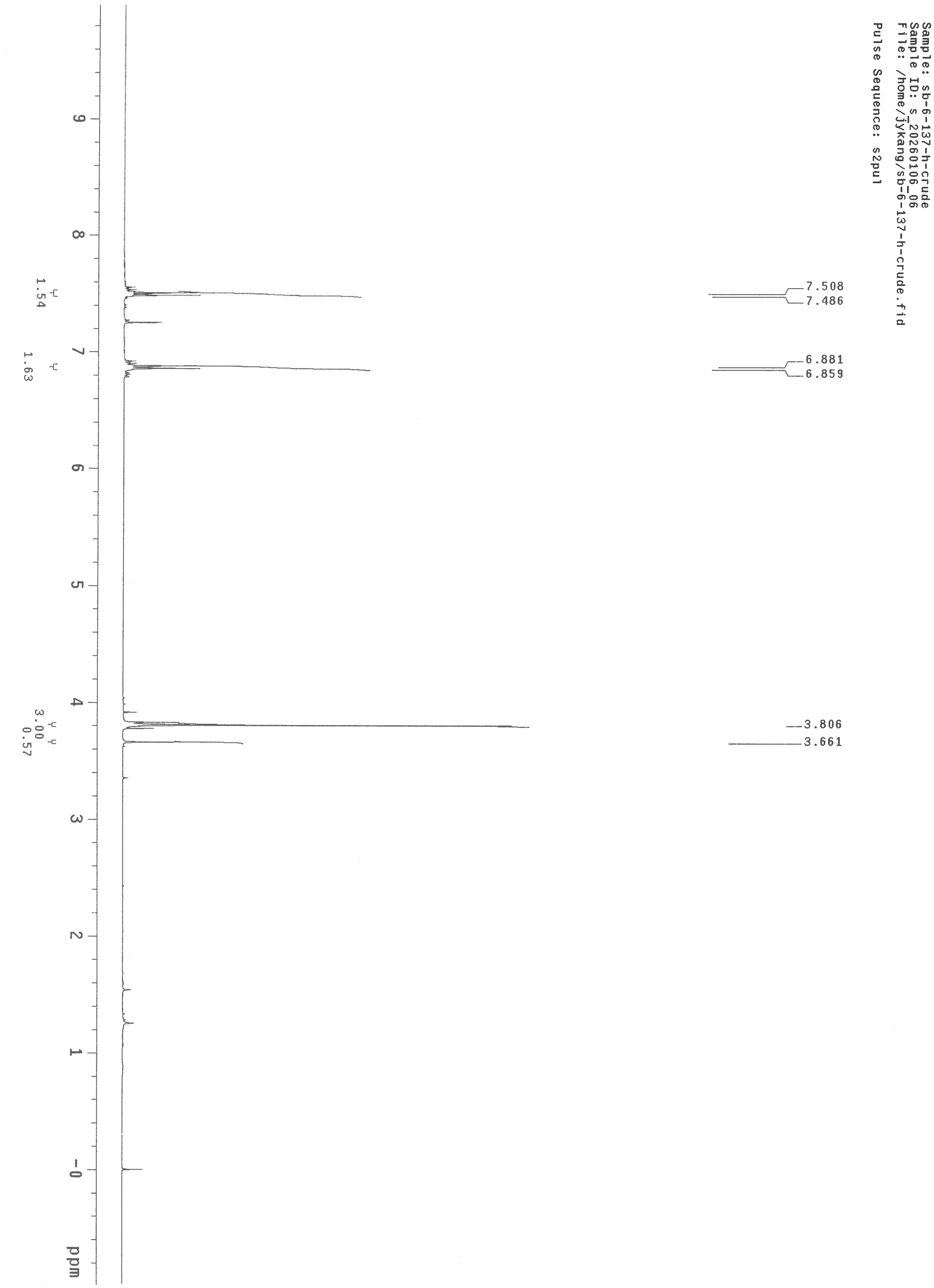

^{13}C NMR (100.5 MHz) in $CDCl_3$

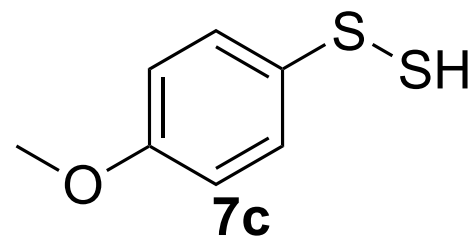

7c

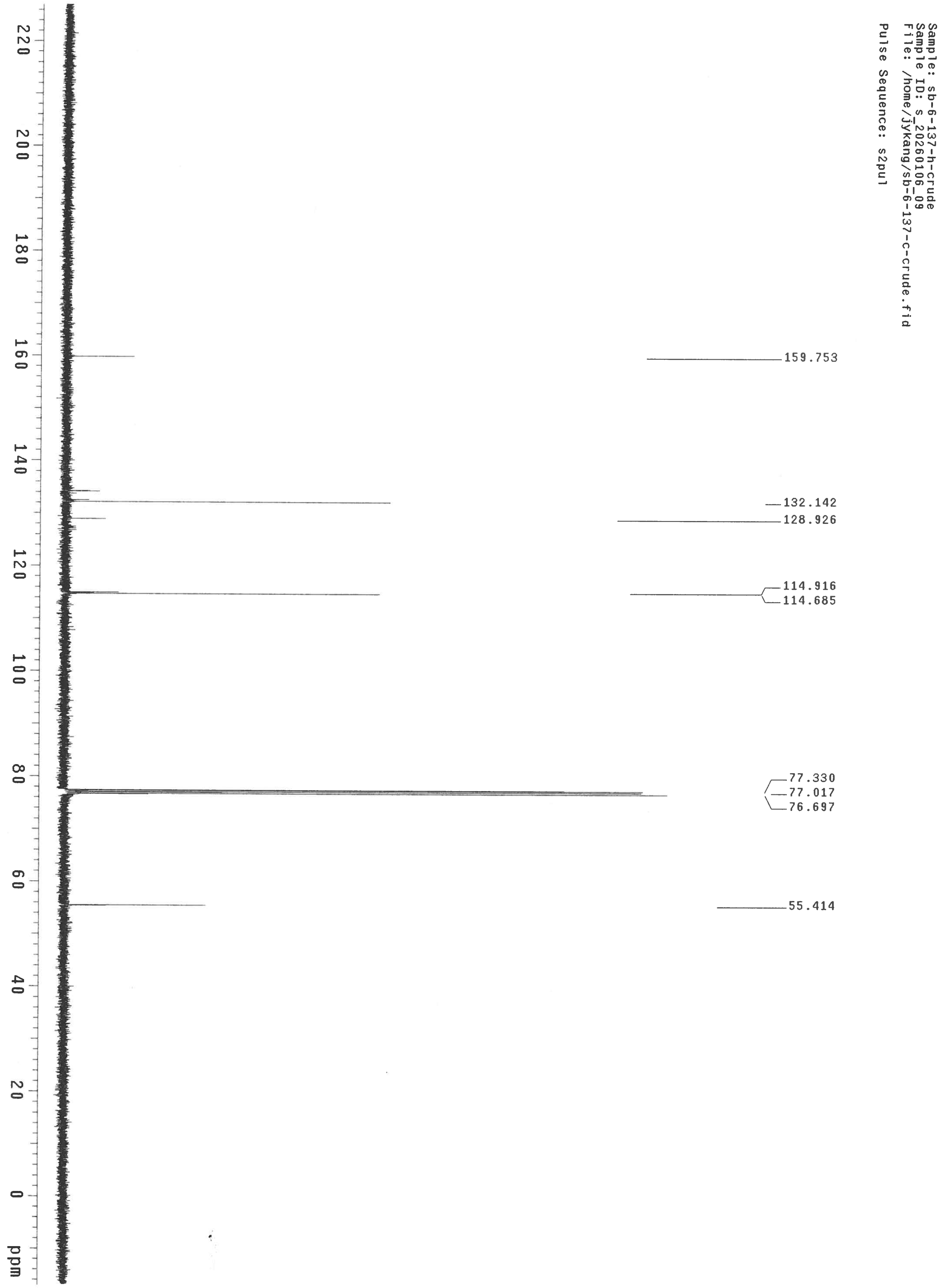

^{1}H NMR (400 MHz) in $CDCl_3$

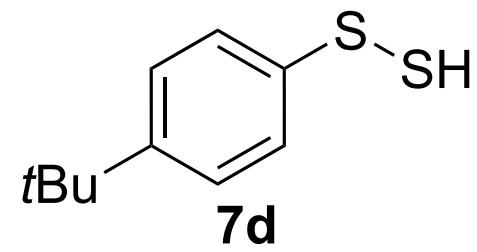

7d

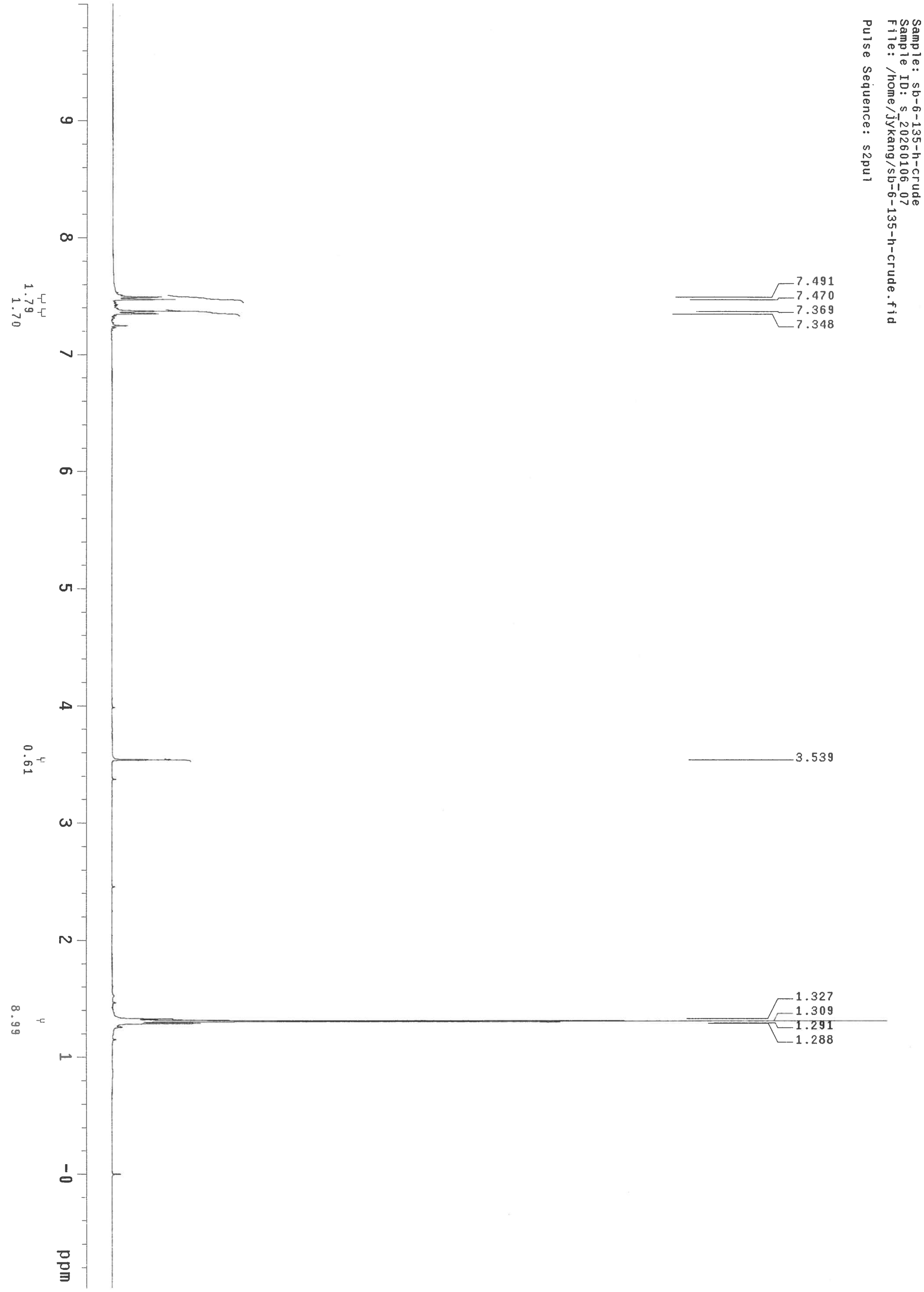

^{13}C NMR (100.5 MHz) in $CDCl_3$

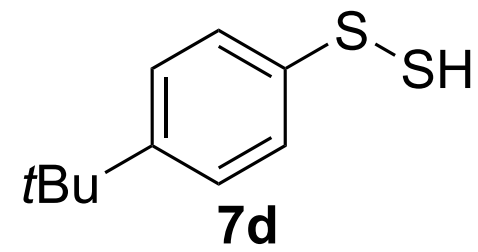

7d

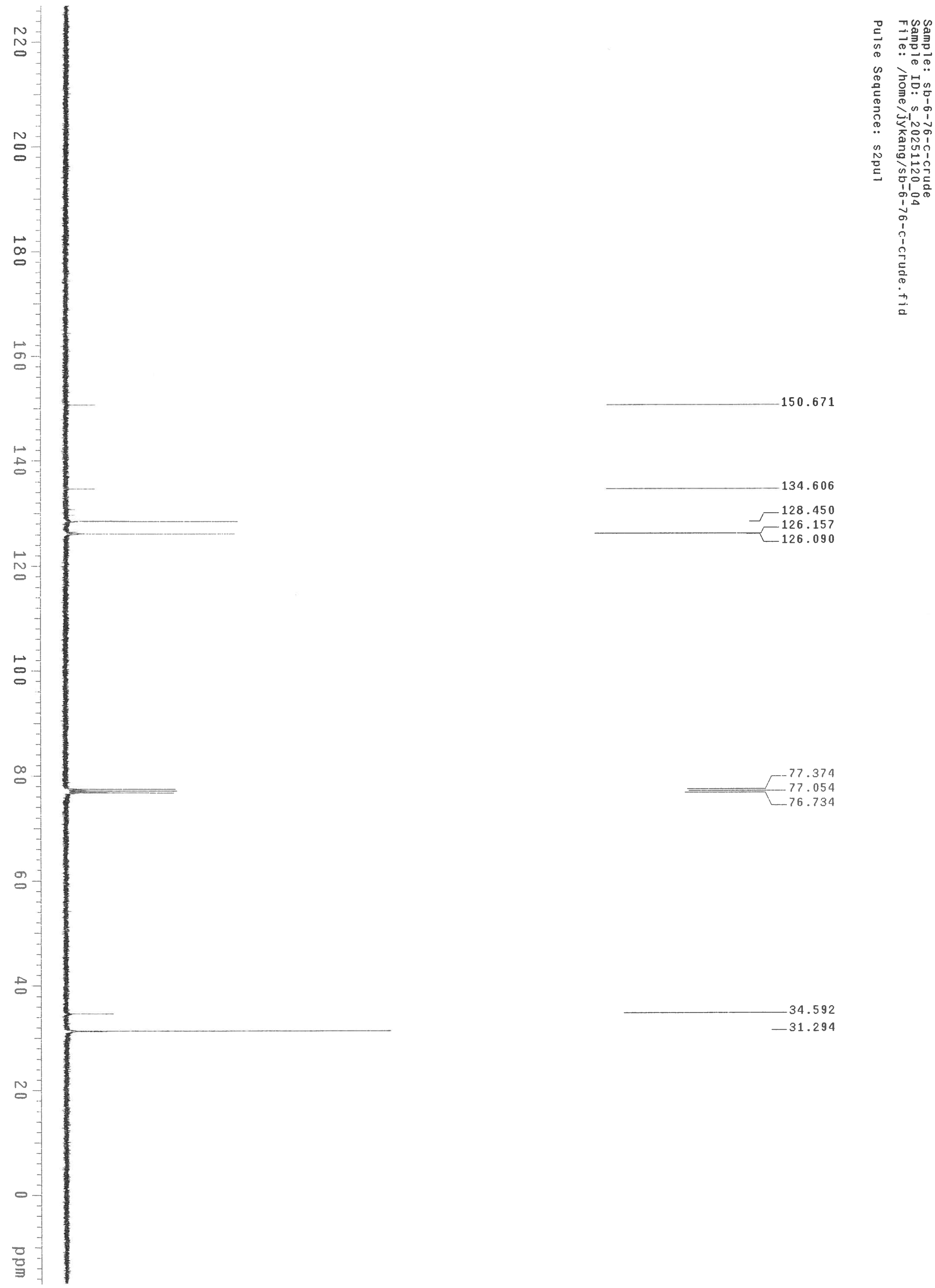

^{1}H NMR (400 MHz) in $CDCl_3$

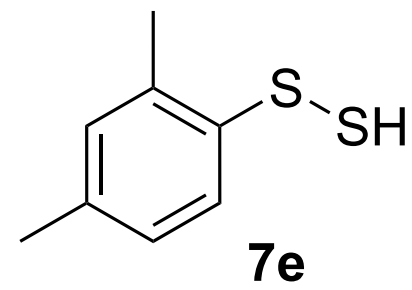

7e

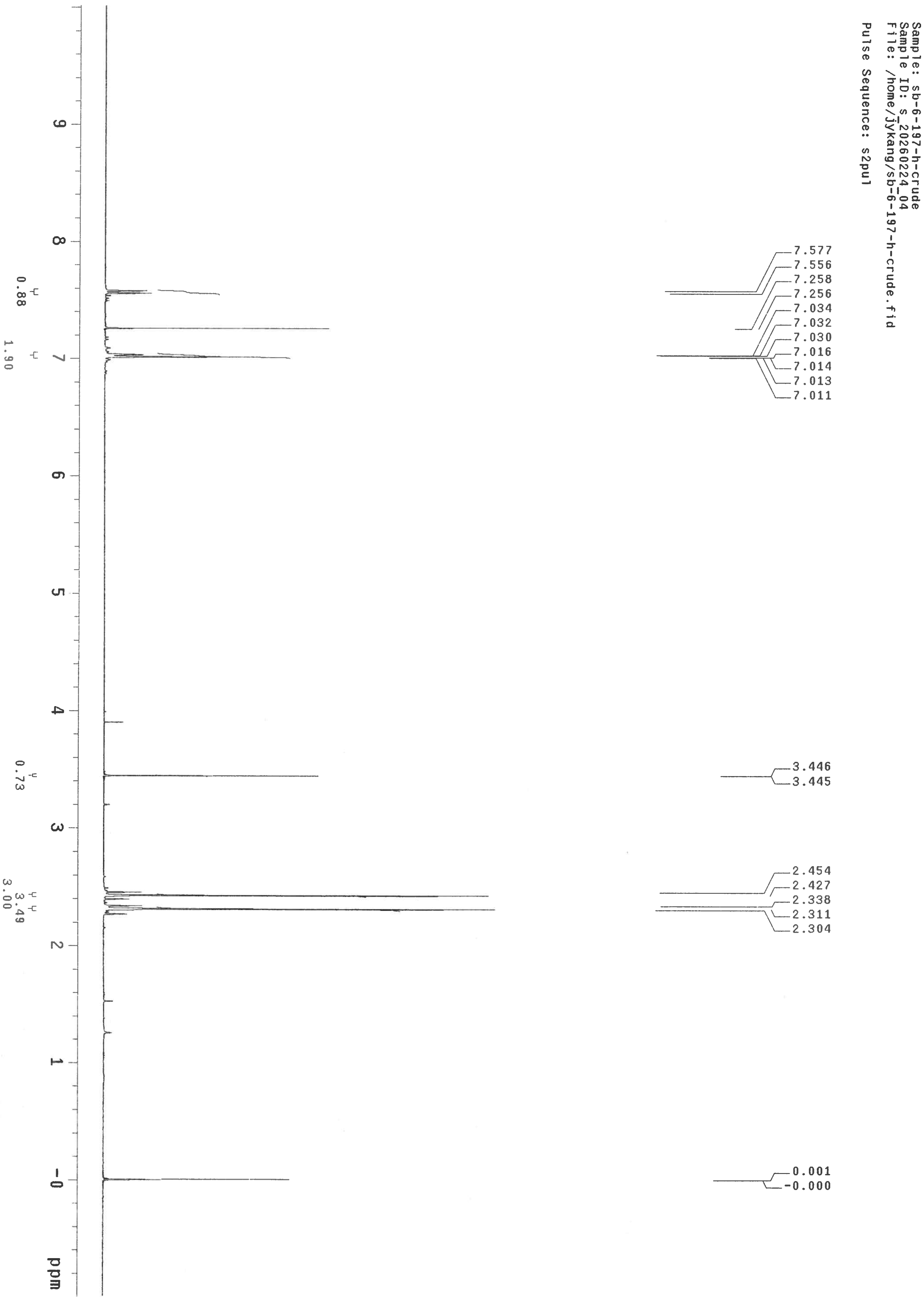

^{13}C NMR (100.5 MHz) in $CDCl_3$

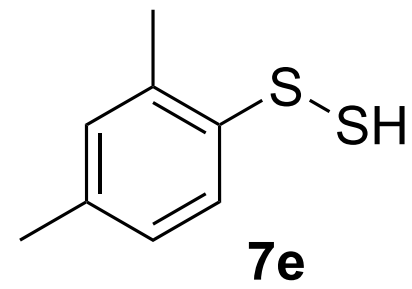

7e

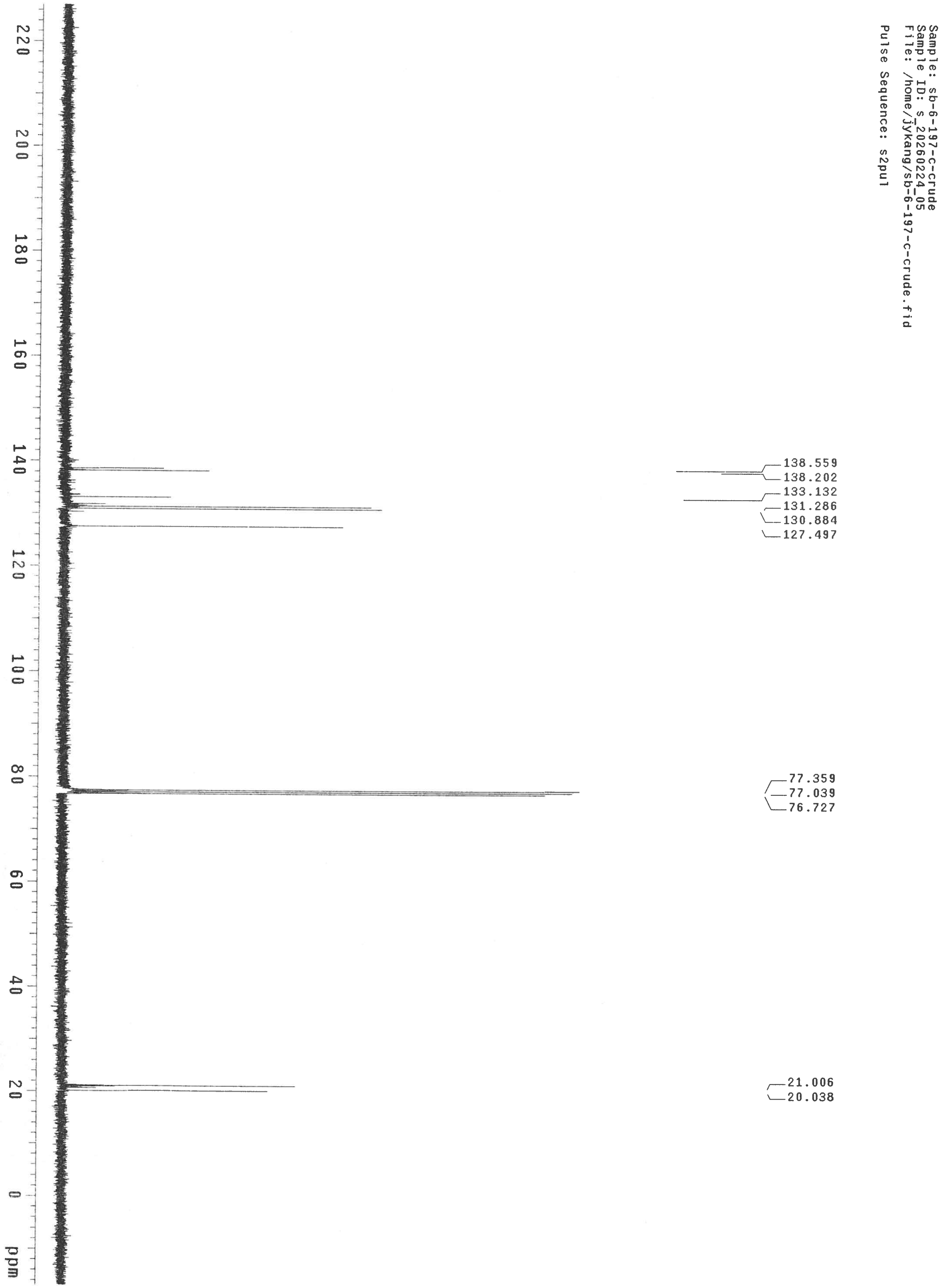

Supplement: Supplementary file 2 [file RA-016-D6RA03773A-s002.pdf]
