# Supplementary material for: A concise synthesis of alkyl, aryl hydropersulfides
Source: RSC Adv. 2026 Jul 6. Online ahead of print. doi: 10.1039/d6ra03773a (PMC13334445; doi:10.1039/d6ra03773a)
Supplement: RA-OLF-D6RA03773A-s001 [file RA-OLF-D6RA03773A-s001.pdf]

Supplementary Information (SI) for RSC Advances.
This journal is © The Royal Society of Chemistry 2026

A Concise Synthesis of Alkyl, Aryl Hydropersulfides

Shishir Bhowmik, Jun Yong Kang*

junyong.kang@unlv.edu

Department of Chemistry and Biochemistry, University of Nevada Las Vegas,

4505 S. Maryland Parkway, Las Vegas, NV 89154-4003

Supporting Information

1. General information……………………………………………………………………………..……**S2**
2. General procedure for the synthesis of acyl thiosuccinimide **1a**………….…………………..……...**S2**
3. General procedure for the synthesis of alkyl acyl persulfide **3**……………….............................**S3**
4. General procedure for the synthesis of alkyl hydropersulfide **4**...**S5**
5. General procedure for the synthesis of aryl acyl persulfide **6**……………..………..............……**S8**
6 General procedure for the synthesis of aryl hydropersulfide **7**…...**S10**
7 Large-scale synthesis of **3a** and **4a**…..**S12**

1. General information

All reactions were carried out under atmospheric conditions in oven-dried glassware with a magnetic stirring bar. Dry solvents (THF, toluene, and DCM) were obtained by a solvent purification system under argon. All commercially available reagents were used as received without further purification. Purification of reaction products was carried out by flash column chromatography using silica gel 60 (230-400 mesh). Analytical thin-layer chromatography was performed on 0.25 mm aluminum-backed silica gel 60-F plates. Visualization was accompanied by UV light and $KMnO_4$ solution. Concentration under reduced pressure refers to the removal of volatiles using a rotary evaporator attached to a dry diaphragm pump (10-15 mm Hg), followed by pumping to a constant weight with an oil pump (<300 mTorr). Infrared (IR) spectra were recorded on an IR spectrometer with KBr wafers or a film on a KBr plate. High-resolution mass spectra (HRMS) were recorded on an LC-MS/IT-TOF mass spectrometer using ESI (electrospray ionization). ^{1}H NMR spectra were recorded in $CDCl_3$ on 400 MHz NMR spectrometer. The ^{1}H chemical shifts are referenced to residual solvent signals at δ 7.26 ($CHCl_3$) or δ 0.00 (TMS). ^{1}H NMR coupling constants (*J*) are reported in Hertz (Hz), and multiplicities are indicated as follows: s (singlet), bs (broad singlet), d (doublet), t (triplet), m (multiplet), dd (doublet of doublet), dt (doublet of triplet). ^{13}C NMR spectra were proton decoupled and recorded in $CDCl_3$ on a 100.5 MHz NMR spectrometer. The ^{13}C chemical shifts are referenced to solvent signals at δ 77.16 ($CDCl_3$). ^{31}P NMR spectra were proton decoupled and recorded in $CDCl_3$ on a 162 MHz NMR spectrometer. ^{31}P chemical shifts are reported relative to 85% H_3PO_4 (0.00 ppm) as an external standard. ^{19}F chemical shifts are reported relative to the external standard (contained in a coaxial capillary), trifluoroacetic acid in $CDCl_3$: δ -76.55 ppm.

2. General procedure for the synthesis of acyl thiosuccinimide (1a)

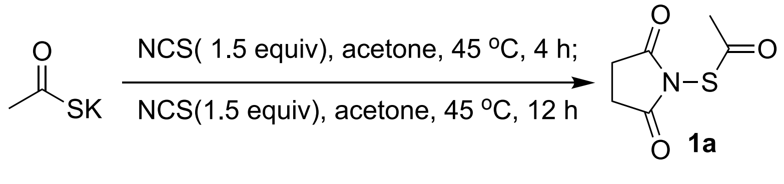

1a

To a solution of N-chlorosuccinimide (NCS) (2992 mg, 22.5 mmol) in acetone (60 mL) was added potassium thioacetate (1710 mg, 15 mmol). The reaction mixture was stirred at 45 °C for 4 h. After stirring for 4 h, the consumption of N-chlorosuccinimide (NCS) was checked with TLC, and another portion of N-chlorosuccinimide (NCS) (2992 mg, 22.5 mmol) was added. The reaction mixture was stirred at 45 °C for 12 h. The crude mixture was filtered, concentrated under reduced

pressure, and purified by flash column chromatography on silica gel eluting with hexane/ethyl acetate [2:1 (v/v)] to afford **1a** as a brown solid.

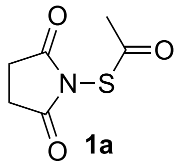

1a

***S*-(2,5-dioxopyrrolidin-1-yl) ethanethioate (1a)**[1]: 1726 mg; 67%; brown solid; R_f = 0.4 (V_{Hexane} / V_{EtOAc} = 1:1)

IR *v* (KBr, cm^{-1}) 2981, 1717, 1304, 1154, 1131, 821, 614

^{1}H NMR (400 MHz, $CDCl_3$) δ 2.97 (s, 4H), 2.39 (s, 3H)

^{13}C NMR (100.5 MHz, $CDCl_3$) δ 189.7, 175.1, 28.8, 26.5

3. General procedure for the synthesis of alkyl acyl persulfide 3

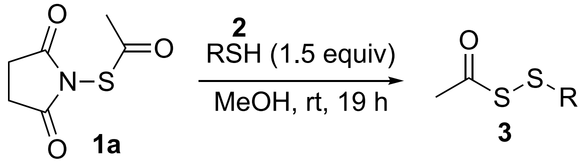

To a solution of acyl-thiosuccinimide **1a** (2 mmol) in methanol (10 mL) was added thiol **2** (3 mmol). The reaction mixture was stirred at room temperature for 19 h. After stirring for 19 h, the crude mixture was concentrated under reduced pressure. The residue was purified by flash column chromatography on silica gel to afford the corresponding product **3**

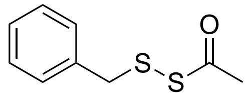

SS-benzyl ethane(dithioperoxoate) (3a)[2]: 316 mg; 80%; white solid; R_f = 0.35 (V_{Hexane} / V_{DCM} = 4:1)

IR *v* (KBr, cm^{-1}) 3027, 2924, 2361, 1729, 1453, 1241, 1108, 953, 597

^{1}H NMR (400 MHz, $CDCl_3$) δ 7.35-7.25(m, 5H), 3.93 (s, 2H), 2.33 (s, 3H)

^{13}C NMR (100.5 MHz, $CDCl_3$) δ 195.0, 136.0, 129.4, 128.5, 127.7, 42.9, 28.6

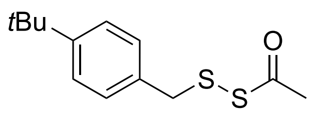

***SS*-(4-(*tert*-butyl)benzyl) ethane(dithioperoxoate) (3b)**[3]: 304 mg; 60%; colorless oil; R_f = 0.7 (V_{Hexane} / V_{EtOAc} = 9:1)

IR *v* (KBr, cm^{-1}) 2958, 2361, 1729, 1700, 1108, 942, 591

^{1}H NMR (400 MHz, $CDCl_3$) δ 7.35 (d, J= 8.0 Hz, 2H), 7.24 (d, J= 7.2 Hz, 2H) 3.91 (s, 2H), 2.34 (s, 3H), 1.30(s, 9H)

^{13}C NMR (100.5 MHz, $CDCl_3$) δ 195.2, 150.8, 132.9, 129.1, 125.5, 42.7, 34.5, 31.2, 28.5.

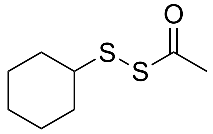

***SS*-cyclohexyl ethane(dithioperoxoate) (3c)**[3]: 277 mg; 73%; colorless oil; R_f = 0.75 (V_{Hexane} / V_{EtOAc} = 9:1)

IR *v* (KBr, cm^{-1}) 2930, 2855, 1729, 1700, 1447, 1350, 1108, 942, 597

^{1}H NMR (400 MHz, $CDCl_3$) δ 2.81-2.77 (m, 1H), 2.45 (s, 3H), 2.01-1.98 (m, 2H), 1.79-1.77 (m, 2H), 1.39-1.22 (m, 6H)

^{13}C NMR (100.5 MHz, $CDCl_3$) δ 195.5, 49.6, 32.5, 28.7, 25.9, 25.4

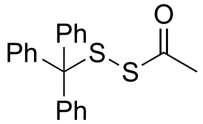

***SS*-trityl ethane(dithioperoxoate) (3d)**[4]: 455 mg; 65%; white solid; R_f = 0.15 (V_{Hexane} / V_{DCM} = 5:1)

IR *v* (KBr, cm^{-1}) 3056, 2361, 1735, 1700, 1488, 1442, 1108, 936, 735, 700, 591

^{1}H NMR (400 MHz, $CDCl_3$) δ 7.36-7.33 (m, 6H), 7.30-7.24 (m, 9H), 2.01 (s, 3H)

^{13}C NMR (100.5 MHz, $CDCl_3$) δ 194.0, 143.2,130.2, 130.1, 127.9, 127.3, 73.2, 28.2

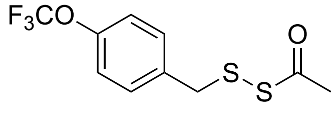

***SS*-(4-(trifluoromethoxy)benzyl) ethane(dithioperoxoate) (3e)**: 219 mg; 39%; colorless oil; R_f = 0.25 (V_{Hexane} / V_{DCM} = 5:1)

IR *v* (KBr, cm^{-1}) 2924, 2361, 1729, 1106, 1511, 1263, 1218, 1166, 1108, 942, 597

^{1}H NMR (400 MHz, $CDCl_3$) δ 7.33 (d, J= 8.8 Hz, 2H), 7.18 (d, J= 8.8 Hz, 2H), 3.91 (s, 2H), 2.36 (s, 3H)

^{13}C NMR (100.5 MHz, $CDCl_3$) δ 194.3, 148.7, 134.8, 130.8, 121.4 (q, J=256 Hz), 41.9, 28.7

^{19}F NMR (375.9 MHz, $CDCl_3$) δ -57.8

Elemental analysis: calcd (%) for $C_{10}H_9F_3O_2S_2$ (282.29): C 42.55, H 3.21; found: C 42.76, H 3.19

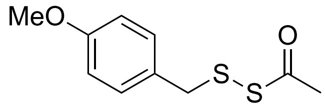

***SS*-(4-methoxybenzyl) ethane(dithioperoxoate) (3f)**[2]: 147 mg; 32%; colorless oil; R_f = 0.35 (V_{Hexane} / V_{DCM} = 4:1)

IR *v* (KBr, cm^{-1}) 2942, 1729, 1700, 1608, 1511, 1459, 1246, 1108, 833

^{1}H NMR (400 MHz, $CDCl_3$) δ 7.24 (d, J= 8.8 Hz, 2H), 6.85 (d, J= 8.8 Hz, 2H), 3.89 (s, 2H), 3.80 (s, 3H), 2.35 (s, 3H)

^{13}C NMR (100.5 MHz, $CDCl_3$) 195.1, 159.2, 130.6, 128.0, 113.9, 55.2, 42.9, 28.6

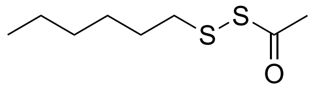

SS-hexyl ethane(dithioperoxoate) (3g)[3]: 146 mg; 38%, colorless liquid, R_f = 0.30 (V_{Hexane} / V_{DCM} = 5:1)

IR *v* (KBr, cm^{-1}) 2958, 2930, 2855, 1729, 1706, 1459, 1350, 1114, 942, 597

^{1}H NMR (400 MHz, $CDCl_3$) δ 2.73 (t, J= 7.6 Hz, 2H), 2.45 (s, 3H), 1.66-1.59 (m, 2H), 1.40-1.35 (m, 2H), 1.31-1.25 (m, 4H), 0.90 (t, J=6.8 Hz, 3H)

^{13}C NMR (100.5 MHz, $CDCl_3$) δ 195.1, 38.9, 31.3, 28.9, 28.8, 28.0, 22.4, 14.0

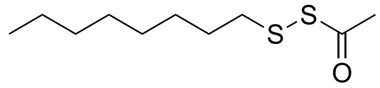

SS-octyl ethane(dithioperoxoate) (3h)[5]: 176 mg; 40%, colorless liquid, R_f = 0.30 (V_{Hexane} / V_{DCM} = 5:1)

IR *v* (KBr, cm^{-1}) 2924, 2855, 2361, 2338, 1729, 1700, 1459, 1108, 936, 597

^{1}H NMR (400 MHz, $CDCl_3$) δ 2.73 (t, J= 7.2 Hz, 2H), 2.45 (s, 3H), 1.64-1.58 (m, 2H), 1.39 (t, J=6.4 Hz, 2H), 1.30-1.27 (m, 8H), 0.89 (t, J=6.8 Hz, 3H)

^{13}C NMR (100.5 MHz, $CDCl_3$) δ 195.2, 38.9, 31.7, 29.1, 29.0, 28.9, 28.8, 28.4, 22.6, 14.0

General procedure for the synthesis of alkyl hydropersulfide 4

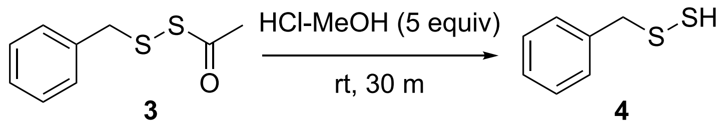

To a solution of **3** (0.5 mmol) in MeOH (1 mL) was added methanolic HCl (5 equiv, 2.5 mmol). The reaction mixture was stirred at room temperature for 30 minutes. After stirring for 30 minutes, the crude mixture was concentrated under reduced pressure to afford alkyl hydropersulfide **4**.

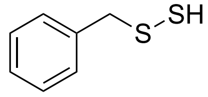

Benzyldisulfane (4a)[1]: 77 mg; 99%; colorless oil, R_f =0.7 (V_{Hexane} / V_{EtOAc} = 9:1)
IR *v* (KBr, cm^{-1}) 3027, 2361, 1493, 1453, 1068, 764, 696
^{1}H NMR (400 MHz, $CDCl_3$) δ 7.34–7.27 (m, 5H), 3.89 (s, 2H), 2.87 (s, 1H)
^{13}C NMR (100.5 MHz, $CDCl_3$) 136.7, 129.2, 128.5, 127.5, 44.7

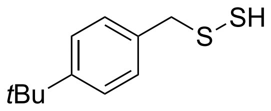

(4-(tert-butyl)benzyl)disulfane (4b): 100 mg; 95%; colorless oil; R_f =0.8 (V_{Hexane} / V_{EtOAc} = 9:1)
IR *v* (KBr, cm^{-1}) 2958, 2361, 1511, 1465, 1413, 1361, 1108, 833
^{1}H NMR (400 MHz, $CDCl_3$) δ 7.36 (d, J= 8.4 Hz, 2H), 7.25 (d, J= 8.4 Hz, 2H), 3.88 (s, 3H), 2.92 (s, 1H), 1.31 (s, 9H)
^{13}C NMR (100.5 MHz, $CDCl_3$) δ 150.5, 133.6, 128.9, 125.5, 44.6, 34.5, 31.3
Elemental analysis: calcd (%) for $C_{11}H_{16}S_2$ (212.36): C 62.21, H 7.59; found: C 62.69, H 7.88

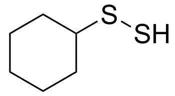

Cyclohexyldisulfane (4c)[6]: 70 mg; 95%; colorless oil; R_f = 0.9 (V_{Hexane} / V_{EtOAc} = 9:1)
IR *v* (KBr, cm^{-1}) 2930, 2849, 2361, 1447, 1263, 993, 884
^{1}H NMR (400 MHz, $CDCl_3$) δ 2.81 (s, 1H), 2.69-2.62 (m, 1H), 2.03-2.00 (m, 2H), 1.82-1.78 (m, 2H), 1.65-1.60 (m, 1H), 1.42-1.20 (m, 5H)
^{13}C NMR (100.5 MHz, $CDCl_3$) δ 49.4, 31.9, 25.9, 25.5

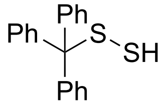

Trityldisulfane (4d)[4]: 143 mg; 93%; white solid; R_f =0.6 (V_{Hexane} / V_{EtOAc} = 9:1)

IR *v* (KBr, cm^{-1}) 3056, 2361, 1488, 1442, 1080, 1034, 758, 735, 700

^{1}H NMR (400 MHz, $CDCl_3$) δ 7.33-7.21 (m, 15H), 2.62 (s, 1H)

^{13}C NMR (100.5 MHz, $CDCl_3$) δ 143.5,129.9, 128.0, 127.1, 70.3

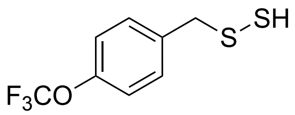

(4-(trifluoromethoxy)benzyl)disulfane (4e): 115 mg; 96%; colorless oil; R_f =0.3 (V_{Hexane} / V_{EtOAc} = 99:1)

IR *v* (KBr, cm^{-1}) 2924, 2361, 1511, 1263, 1218, 1166, 838, 677

^{1}H NMR (400 MHz, $CDCl_3$) δ 7.34 (d, J= 8.4 Hz, 2H), 7.19 (d, J= 8 Hz, 2H) 3.87 (s, 3H), 2.88 (s, 1H)

^{13}C NMR (100.5 MHz, $CDCl_3$) δ 148.5, 135.5, 130.6, 121.0 (q, J= 256.6 Hz), 120.99, 43.8

^{19}F NMR (375.9 MHz, $CDCl_3$) δ -57.8

HRMS(ESI): found $[M+H]^+$ values corresponding to 4-(trifluoromethoxy)phenyl)methanethiol; m/z calcd. for $C_8H_7F_3OS$ ($[M+H]^+$): 209.0248; found 209.0858.

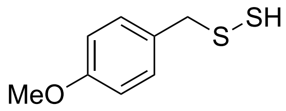

(4-Methoxybenzyl)disulfane (4f)[1]: 89 mg; 96%; colorless oil; R_f = 0.6 (V_{Hexane} / V_{EtOAc} = 9:1)

IR *v* (KBr, cm^{-1}) 2953, 2832, 1608, 1511, 1298, 1246, 1177, 1034, 833

^{1}H NMR (400 MHz, $CDCl_3$) δ 7.23 (d, J= 8.4 Hz, 2H), 6.87 (d, J= 8.8 Hz, 2H), 3.85 (s, 2H), 3.80 (s, 3H), 2.87 (s, 1H)

^{13}C NMR (100.5 MHz, $CDCl_3$) 159.0, 130.4, 128.7, 113.9, 55.2, 44.3

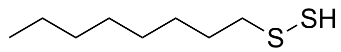

Octyldisulfane (4h): 64 mg; 72%; colorless liquid; R_f =0.9 (V_{Hexane} / V_{EtOAc} = 9:1)

IR *v* (KBr, cm^{-1}) 2953, 2924, 2855, 2361, 1459, 867, 723

^{1}H NMR (400 MHz, $CDCl_3$) δ 2.96 (s, 1H), 2.70 (t, J=7.2 Hz, 2H), 1.72-1.65 (m, 2H), 1.39-1.36 (m, 2H), 1.34-1.28 (m, 8H), 0.90 (t, J= 6.4 Hz, 3H)

^{13}C NMR (100.5 MHz, $CDCl_3$) δ 40.2, 31.7, 29.1, 28.3, 28.2, 22.6, 14.0

Elemental analysis: calcd (%) for $C_8H_{18}S_2$ (178.35): C 53.88, H 10.17; found: C 53.62, H 10.09

General procedure for the synthesis of aryl acyl persulfide 6

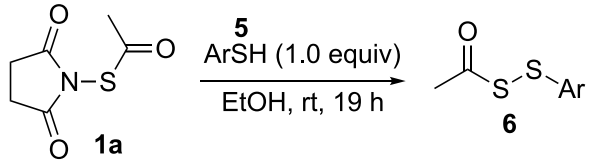

To a solution of acyl-thiosuccinimide **1a** (2 mmol) in ethanol (10 mL) was added thiophenol **5** (2mmol). The reaction mixture was stirred at room temperature for 19 h. After stirring for 19 hr the crude mixture was concentrated under reduced pressure. The residue was purified by flash column chromatography on silica gel to afford the corresponding product **6**

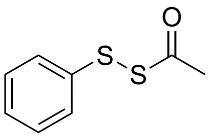

***SS*-phenyl ethane(dithioperoxoate) (6a)**[7]: 213 mg; 58%; colorless oil; R_f =0.6 (V_{Hexane} / V_{EtOAc} = 9:1)

IR *v* (KBr, cm^{-1}) 3056, 2361, 1735, 1706, 1476, 1436, 1108,942, 741, 689, 591

^{1}H NMR (400 MHz, $CDCl_3$) δ 7.51-7.49 (m, 2H), 7.31-7.28 (m, 3H), 2.47 (s, 3H)

^{13}C NMR (100.5 MHz, $CDCl_3$) δ 194.1, 135.7, 130.2, 129.1, 128.3, 28.7

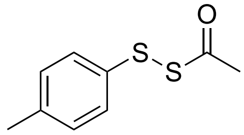

***SS*-(p-tolyl) ethane(dithioperoxoate) (6b)**[8]: 182 mg; 46%; colorless oil; R_f =0.6 (V_{Hexane} / V_{EtOAc} = 9:1)

IR *v* (KBr, cm^{-1}) 3430, 2361, 1729, 1700, 1648, 1488, 1108, 942, 804, 591

^{1}H NMR (400 MHz, $CDCl_3$) δ 7.42 (d, J= 8.4 Hz, 2H), 7.10 (d, J= 8.4 Hz, 2H), 2.44 (s, 3H), 2.32 (s, 3H)

^{13}C NMR (100.5 MHz, $CDCl_3$) δ 194.2, 138.8, 132.3, 131.2, 129.9, 28.7, 21.1

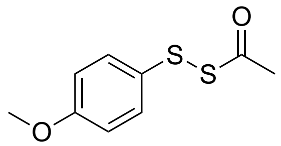

SS-(4-methoxyphenyl) ethane(dithioperoxoate) (6c)[8]: 235 mg; 55%; colorless oil; R_f =0.5 (V_{Hexane} / V_{EtOAc} = 9:1)

IR *v* (KBr, cm^{-1}) 3004, 2361, 1729, 1591, 1493, 1246, 1108, 1028, 942, 827

^{1}H NMR (400 MHz, $CDCl_3$) δ 7.53 (d, J= 8.8 Hz, 2H), 6.83 (d, J= 8.8 Hz, 2H), 3.79 (s, 3H), 2.43(s, 3H)

^{13}C NMR (100.5 MHz, $CDCl_3$) δ 194.3, 160.5, 134.5, 126.5, 114.7, 55.3, 28.8

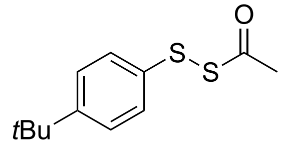

SS-(4-(tert-butyl)phenyl) ethane(dithioperoxoate) (6d)[8]: 220 mg; 46%; colorless oil; R_f = 0.3 (V_{Hexane} / V_{DCM} = 4:1)

IR *v* (KBr, cm^{-1}) 2964, 2907, 2866, 1735, 1706, 1488, 1396, 1355, 1269, 1108, 1011, 942, 827

^{1}H NMR (400 MHz, $CDCl_3$) δ 7.45 (d, J= 8.8 Hz, 2H), 7.32 (d, J= 8.4 Hz, 2H), 2.46 (s, 3H), 1.29 (s, 9H)

^{13}C NMR (100.5 MHz, $CDCl_3$) δ 194.3, 151.9, 132.2, 130.7, 126.2, 34.6, 31.1, 28.7

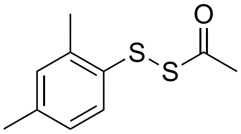

***SS*-(2,4-dimethylphenyl) ethane(dithioperoxoate) (6e)**[9]: 106 mg; 25%; colorless oil; R_f = 0.3 (V_{Hexane} / V_{DCM} = 5:1)

IR *v* (KBr, cm^{-1}) 2918, 2183, 1739, 1706, 1602, 1467, 1442, 1108, 1045, 942, 810

^{1}H NMR (400 MHz, $CDCl_3$) δ7.48 (d, J= 8 Hz, 1H), 7.01 (s, 1H), 6.96 (d, J= 8 Hz, 1H), 2.48 (s, 3H), 2.42 (s, 3H), 2.29 (s, 2H)

^{13}C NMR (100.5 MHz, $CDCl_3$) δ 194.2, 139.7, 139.2, 132.8, 131.4, 130.9, 127.6, 28.8, 21.0, 20.5

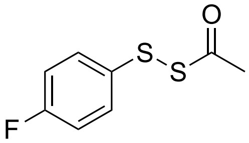

SS-(4-fluorophenyl) ethane(dithioperoxoate) (6f)[8]: 88 mg; 22%, colorless liquid, R_f = 0.25 (V_{Hexane} / V_{DCM} = 5:1)

IR *v* (KBr, cm^{-1}) 2942, 1735, 1585, 1488, 1350, 1229, 1154, 1108, 942, 827, 591

^{1}H NMR (400 MHz, $CDCl_3$) δ 7.54 (dd, J=8.8 Hz, 5.2 Hz, 2H), 7.00 (dd, J=9.2 Hz, 8.4 Hz, 2H), 2.45 (s, 3H)

^{13}C NMR (100.5 MHz, $CDCl_3$) δ 193.7, 163.1 (d, J =249.8 Hz), 133.7 (d, J= 8.2 Hz), 131.1 (d, J= 3.71 Hz), 116. 4 (d, J= 22.4 Hz), 28.8
^{19}F NMR (375.9 MHz, $CDCl_3$) δ -111.8

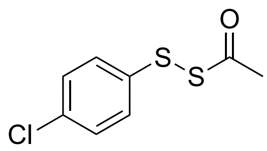

SS-(4-chlorophenyl) ethane(dithioperoxoate) (6g)[8]: 87 mg; 20%, colorless liquid, R_f = 0.3 (V_{Hexane} / V_{DCM} = 5:1)
IR *v* (KBr, cm^{-1}) 2924, 1735, 1706, 1470, 1390, 1350, 1108, 1091, 1011, 942, 815, 591
^{1}H NMR (400 MHz, $CDCl_3$) δ 7.45 (d, J= 8.8 Hz, 2H), 7.28 (d, J= 8.8 Hz, 2H), 2.47 (s, 3H)
^{13}C NMR (100.5 MHz, $CDCl_3$) δ 193.4, 134.6, 134.2, 131.8, 129.3, 28.8

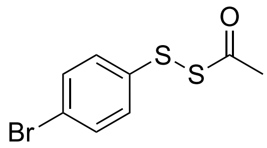

SS-(4-bromophenyl) ethane(dithioperoxoate) (6h)[8]: 126 mg; 24%, colorless liquid, R_f = 0.3 (V_{Hexane} / V_{DCM} = 5:1)
IR *v* (KBr, cm^{-1}) 2918, 2361, 2338, 1735, 1706, 1470, 1384, 1350, 1108, 1068, 1005, 942, 810, 591
^{1}H NMR (400 MHz, $CDCl_3$) δ 7.43 (d, J= 11.2 Hz, 2H), 7.37 (d, J= 8.8 Hz, 2H), 2.47 (s, 3H)
^{13}C NMR (100.5 MHz, $CDCl_3$) δ 193.4, 134.9, 132.2, 131.9, 122.6, 28.8

General procedure for the synthesis of aryl hydropersulfide 7

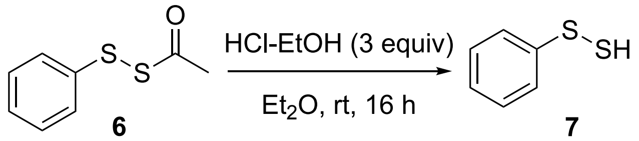

To a solution of **6** (0.5 mmol) in Et_2O (1 mL) was added ehanolic HCl (3 equiv, 1.5 mmol). The reaction mixture was stirred at room temperature for 16 h. After stirring for 16 h, the crude mixture was concentrated under reduced pressure to afford aryl hydropersulfide **7**.

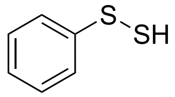

phenyldisulfane (7a)[8]: 70 mg; 99%; colorless liquid; R_f =0.6 (V_{Hexane} / V_{EtOAc} = 9:1)

IR *v* (KBr, cm^{-1}) 2361, 2338, 1648, 741

^{1}H NMR (400 MHz, $CDCl_3$) δ 7.55 (d, J= 7.6 Hz, 2H), 7.34 (t, J = 7.2 Hz, 2H), 7.24 (d, J=6.8 Hz, 1H), 3.51 (s, 1H)

^{13}C NMR (100.5 MHz, $CDCl_3$) δ 137.9, 128.9, 127.8, 127.0

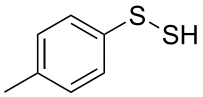

p-tolyldisulfane (7b)[8]: 75 mg; 96%; colorless liquid; R_f =0.65 (V_{Hexane} / V_{EtOAc} = 9:1)

IR *v* (KBr, cm^{-1}) 2918, 2361, 2338, 1488, 1103, 1016, 804

^{1}H NMR (400 MHz, $CDCl_3$) δ 7.44 (d, J= 8.0 Hz, 2H), 7.14 (d, J= 8Hz, 2H), 3.55 (s, 1H), 2.34 (s, 3H)

^{13}C NMR (100.5 MHz, $CDCl_3$) δ 137.4, 134.6, 129.7, 128.8, 21.0

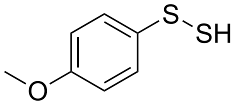

(4-methoxyphenyl)disulfane (7c)[8]: 86 mg; >99%; colorless oil; R_f = 0.7 (V_{Hexane} / V_{EtOAc} = 9:1);

IR *v* (KBr, cm^{-1})2935, 2361, 2338, 1591, 1493,1459, 1286, 1246, 1177, 1028, 821

^{1}H NMR (400 MHz, $CDCl_3$) δ 7.49 (d, J= 8.8 Hz, 2H), 6.87 (d, J= 8.8 Hz, 2H), 3.80 (s, 3H), 3.66 (s, 1H)

^{13}C NMR (100.5 MHz, $CDCl_3$) δ 159.7, 132.1, 128.9, 114.9, 55.4

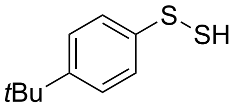

(4-(*tert*-butyl)phenyl)disulfane (7d)[8]: 96 mg; 97%; colorless liquid; R_f= 0.8 (V_{Hexane} / V_{DCM} = 5:1)

IR *v* (KBr, cm^{-1}) 2958, 2361, 2338, 1488, 1459, 1396, 1269, 1114, 1011, 821

^{1}H NMR (400 MHz, $CDCl_3$) δ 7.49 (d, J= 8.4 Hz, 2H), 7.36 (d, J= 8.4 Hz, 2H), 3.54 (s, 1H), 1.30 (s, 9H)

^{13}C NMR (100.5 MHz, $CDCl_3$) δ 150.6, 134.6, 128.4, 126.0, 34.5, 31.2

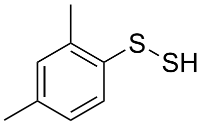

(2,4-dimethylphenyl)disulfane (7e): 84 mg; 99%; colorless liquid; R_f = 0.8 (V_{Hexane} / V_{EA} = 9:1)

IR *v* (KBr, cm^{-1}) 3430, 2981, 2183, 1729, 1637, 1258, 1108, 1022, 965

^{1}H NMR (400 MHz, $CDCl_3$) δ 7.56 (d, J= 8.4 Hz, 1H), 7.03-7.01 (m, 2H), 3.44 (s, 1H), 2.42 (s, 3H), 2.31 (s, 3H)

^{13}C NMR (100.5 MHz, $CDCl_3$) δ 138.5, 138.2, 133.1, 131.2, 130.8, 127.4, 21.0, 20.0

Elemental analysis: calcd (%) for $C_8H_{10}S_2$ (170.00): C 56.43, H 5.92; found: C 56.84, H 5.66

Large-scale synthesis of 3a

To a solution of acyl thiosuccinimide **1a** (10 mmol) in methanol (50 mL) was added benzyl mercaptan **2a** (15 mmol). The reaction mixture was stirred at room temperature for 19 h. After stirring for 19 h, the crude mixture was concentrated under reduced pressure. The residue was purified by flash column chromatography on silica gel eluting with hexane/DCM [5:1 (v/v)] to afford the corresponding product **3a** (1.23 g, 62%)

Large-scale synthesis of 4a

To a solution of benzyl acyl persulfide **3a** (2.5 mmol) in MeOH (5 mL) was added methanolic HCl (5 equiv, 12.5 mmol). The reaction mixture was stirred at room temperature for 30 minutes. After stirring for 30 minutes, the crude mixture was concentrated under reduced pressure to afford benzyl hydropersulfide **4a** (390 mg, >99%)

References

1. J. Ash, K. Curtis, S. Bhowmik, S. O. Odoh and J. Y. Kang, *The Journal of Organic Chemistry*, 2025, **90**, 1167-1174.
2. X. Xiao, M. Feng and X. Jiang, *Angewandte Chemie International Edition*, 2016, **55**, 14121-14125.
3. X. Zhou, Y. Jiang, J. Li, J. Wang, J. Chen, Y. Yu and H. Cao, *The Journal of Organic Chemistry*, 2024, **89**, 6684-6693.
4. T. S. Bailey, L. N. Zakharov and M. D. Pluth, *Journal of the American Chemical Society*, 2014, **136**, 10573-10576.
5. X. Xiao, J. Xue and X. Jiang, *Nature Communications*, 2018, **9**, 2191.
6. L. Field, J. V. Ravan, J. D. Dunkel, J. A. Waites, D. W. White, N. E. Heimer and R. A. Neal, *The Journal of Organic Chemistry*, 1982, **47**, 4651-4654.

7. Y. Yu, X. Zhou, J. Wang, Y. Jiang and H. Cao, *Organic Letters*, 2023, **25**, 8937-8941.
8. J. Tsurugi, S. Kawamura and T. Horii, *The Journal of Organic Chemistry*, 1971, **36**, 3677-3680.
9. T. Fujisawa and N. Kobayashi, *The Journal of Organic Chemistry*, 1971, **36**, 3546-3549.
